# Human Cerebrospinal Fluid Promotes Neuronal Circuit Maturation of Human Induced Pluripotent Stem Cell-Derived 3D Neural Aggregates

**DOI:** 10.1016/j.stemcr.2020.05.006

**Published:** 2020-06-09

**Authors:** Julia Izsak, Henrik Seth, Stephan Theiss, Eric Hanse, Sebastian Illes

**Affiliations:** 1 Institute of Neuroscience and Physiology, Sahlgrenska Academy at University of Gothenburg, Gothenburg, Sweden; 2 Institute of Clinical Neuroscience and Medical Psychology, Medical Faculty, Heinrich Heine University, Düsseldorf, Germany; 3 Result Medical GmbH, Düsseldorf, Germany

**Keywords:** Human induced pluripotent stem cells, neural stem cells, neurogenesis, astrocyte development, synaptogenesis, human cerebrospinal fluid, hiPSC-3D-neural models, neuronal networks, microelectrode array

## Abstract

Human induced pluripotent stem cell (hiPSC)-derived *in vitro* neural and organoid models resemble fetal, rather than adult brain properties, indicating that currently applied cultivation media and supplements are insufficient to achieve neural maturation beyond the fetal stage. *In vivo*, cerebrospinal fluid molecules are regulating the transition of the immature fetal human brain into a mature adult brain. By culturing hiPSC-3D neural aggregates in human cerebrospinal fluid (hCSF) obtained from healthy adult individuals, we demonstrate that hCSF rapidly triggers neurogenesis, gliogenesis, synapse formation, neurite outgrowth, suppresses proliferation of residing neural stem cells, and results in the formation of synchronously active neuronal circuits *in vitro* within 3 days. Thus, a physiologically relevant and adult brain-like milieu triggers maturation of hiPSC-3D neural aggregates into highly functional neuronal circuits *in vitro*. The approach presented here opens a new avenue to identify novel physiological factors for the improvement of hiPSC neural *in vitro* models.

## Highlights

• Human CSF triggers rapidly multiple maturation processes in human 3D neural models
• Human CSF triggers human neurogenesis and suppresses neural stem cell proliferation
• Human CSF triggers human astrocyte development, neurite growth, and synapse formation
• Human CSF triggers the maturation of neurons into highly functional neuronal circuits

## Graphical Abstract

**Figure:**
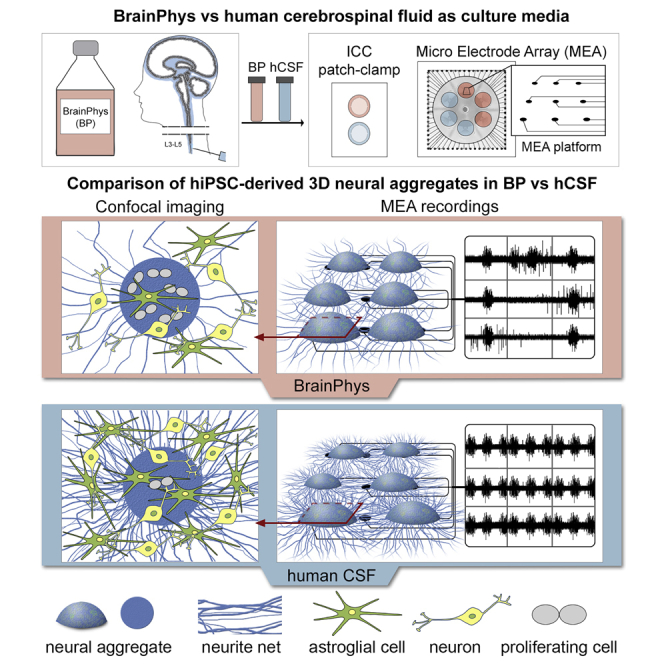

## Teaser

In this article, Izsak and colleagues show that human cerebrospinal fluid triggers rapid maturation of human iPSC-3D neural aggregates indicated by neural stem cell quiescence, enhanced neurite growth, synapse development, neuronal as well as astrocyte differentiation, and formation of highly functional neuronal circuits *in vitro*.

## Introduction

Human cerebrospinal fluid (hCSF) flows within the ventricular system, central canal, and subarachnoid space, and surrounds the central nervous system. Molecules within the CSF regulate cellular processes within the embryonic and adult brain (Bachy et al., 2008, Obernier and Alvarez-Buylla, 2019, Zappaterra and Lehtinen, 2012).

Human induced pluripotent stem cell (hiPSC)-derived neural cells are used as *in vitro* models to obtain insights into brain development and human neuronal function. For decades, DMEM and neurobasal culture media have been used as the cell culture environment for human- and animal-derived neurons *in vitro*. Bardy et al. (2015) presented BrainPhys (BP) medium with adjusted major ion concentrations identical to hCSF. Because Bardy et al. showed that electrophysiological function of hiPSC neurons cultured in BP is superior to previously used DMEM and Neurobasal culture medium, BP is currently considered as the most physiologically relevant culture medium for hiPSC-derived neurons *in vitro* (Livesey, 2015). A comparison of neuronal circuit development and function of *in vitro* neurons cultured in BP-based medium or hCSF represents an interesting, however, yet unaddressed approach.

Ongoing proliferation of neural stem cells (NSCs) and limited neuronal maturation are common phenomena described for 2D and 3D hiPSC-derived neural *in vitro* models (Kirwan et al., 2015, Lancaster et al., 2013, Qian et al., 2019). Application of neurotrophic factors (e.g., BDNF, GDNF) alone is insufficient to suppress ongoing proliferation. Application of small molecules, such as DAPT or PD0332991, are used to mediate quiescence of NSCs and neuronal differentiation (Borghese et al., 2010, Kemp et al., 2016, Kirkeby et al., 2012, Rushton et al., 2013). However, the use of small molecules represents rather an artificial approach to trigger maturation in human neural *in vitro* models. Therefore, exposing hiPSC-derived neural cells to hCSF would allow to study the functional and cellular maturation of hiPSC-derived neural cells in a physiologically relevant environment *in vitro*.

In the present study, we analyzed the functional and cellular impact of hCSF on hiPSC-3D neural aggregates (3D NAs) comprised of cortical neurons, glial cells, and residing proliferative neural cells (Edri et al., 2015, Izsak et al., 2019). For this purpose, we applied patch-clamp technique, microelectrode array (MEA) recordings, and confocal imaging on 3D NAs exposed to hCSF obtained from healthy adult individuals. After changing from BP-based medium to hCSF, 3D NAs showed a tremendous increase of neuronal network activity within 3 days, which was not reversible and lasted for several weeks. Immunocytochemical staining and confocal imaging revealed that hCSF caused an immediate increase of neurite net formation, synapse formation, astroglial and neuronal development, as well as suppressed proliferation of residing NSCs in 3D NAs. Our work demonstrates that hCSF improves the maturation of neuronal circuits in a human 3D neural *in vitro* model.

## Results

### Properties of Adherently Growing hiPSC-3D NA Cultures

**Figure 1:**
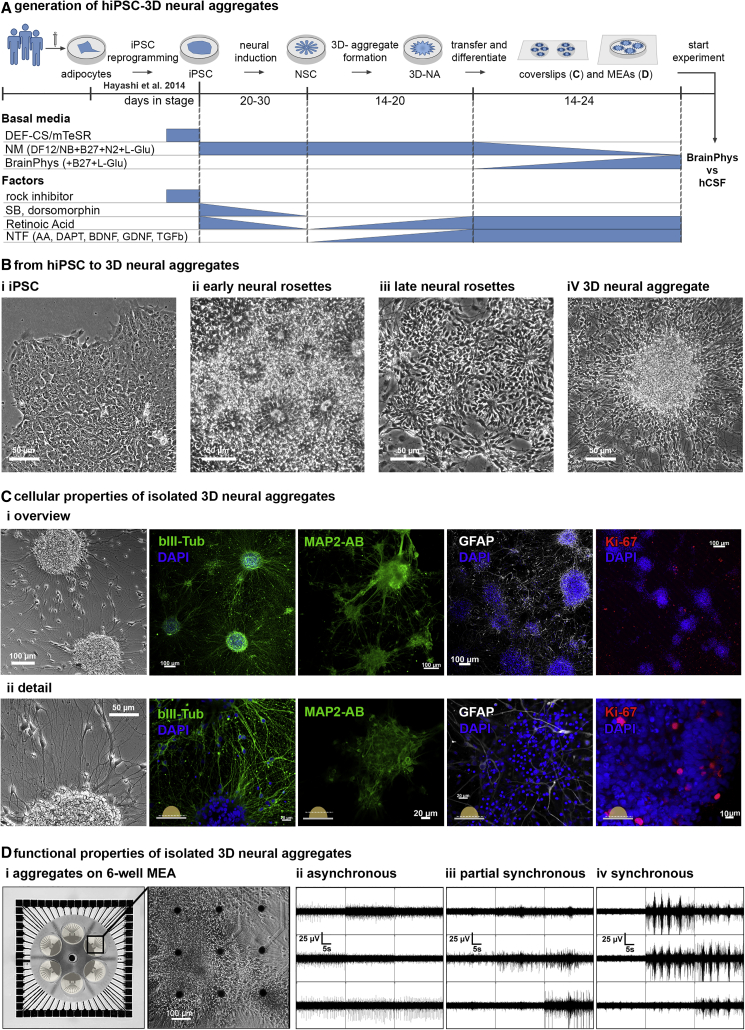
Generation of hiPSC-Derived 3D NAs (A) Schematic representation of the *in vitro* generation of hiPSC-3D NAs. (B) Phase-contrast images show the morphology of hiPSC (i), early neural rosettes (14 days *in vitro* [DIV]) (ii), late neural rosettes (30 DIV) (iii), and 3D NAs (50 DIV) (iv). (C) Overview (i) and detailed images (ii) of βIII-tubulin^+^ and MAP2-AB^+^ neurons, GFAP^+^ cells, and Ki-67^+^ proliferating cells within 3D NAs. Schematic drawings in (ii) illustrate z levels of image acquisition within 3D NAs. (D) Image shows a six-well MEA chip comprising nine microelectrodes per well and phase-contrast image shows cultured 3D NAs (20 DIV) (i). MEA recordings show asynchronous (ii), partial synchronous (iii), and synchronous (iv) neuronal population activities. Each box represents the spiking and bursting activity detected by one electrode. NM, neuronal medium; AA, ascorbic acid; NTF, neurotrophic factors.

We applied the commonly used “dual-SMAD-inhibition” protocol for neural differentiation of hiPSC into cortical NSCs (Izsak et al., 2019, Vizlin-Hodzic et al., 2017) (Figure 1A). hiPSC-derived cortical NSCs grow as neural rosettes (Figure 1B, ii, iii), which give rise to adherently growing 3D NAs (Figure 1B, iv) (Edri et al., 2015, Izsak et al., 2019). For further assessment of cellular and electrophysiological properties, isolated 3D NAs (Figure 1C) were cultured either on glass coverslips or on six-well MEA chips.

Immunocytochemistry and confocal imaging show that adherently growing 3D NAs consist of astroglial cells and cortical neurons (Figures 1C and S1). However, we observed ongoing growth of 3D NAs and the presence of Ki-67 proliferating NSCs (Figure 1C) (Izsak et al., 2019). To describe the development and properties of neuronal network activities generated by neurons in 3D NAs, multi-site extracellular recordings were performed on 3D NAs that adherently grow on nine microelectrodes per well (Figure 1D, i). Each microelectrode allows extracellular recording of spiking and bursting activity generated by neurons in close vicinity (up to 75 μm) to the microelectrode. We demonstrate that neurons within adherently growing 3D NA cultures form functional neuronal circuits that either show asynchronous (Figure 1D, ii), partially synchronous (Figure 1D, iii), or synchronous (Figure 1D, iv) neuronal population activity within 3 weeks in culture. Asynchronous activity is characterized by spontaneous uncorrelated spiking and bursting activity detected by few electrodes and no synchronization of bursts across electrodes (Figure 1D, ii). Partially synchronously active neuronal networks show spontaneous synchronous bursts (Figure 1D, iii), defined as population bursts, which show an irregular pattern. Synchronously active neuronal networks show spontaneous population bursts with a regular firing pattern (Figure 1D, iv).

Since 3D NAs are comprised of highly functional neurons and astroglial cells, however retain immature properties, such as ongoing proliferation, we used adherently growing hiPSC-3D NAs to describe the influence of hCSF on a complex human 3D neural *in vitro* model.

### Adherently Growing Human iPSC-3D NAs Cultured in Healthy hCSF Show Increased Synchronous Neuronal Network Activity

**Figure 2:**
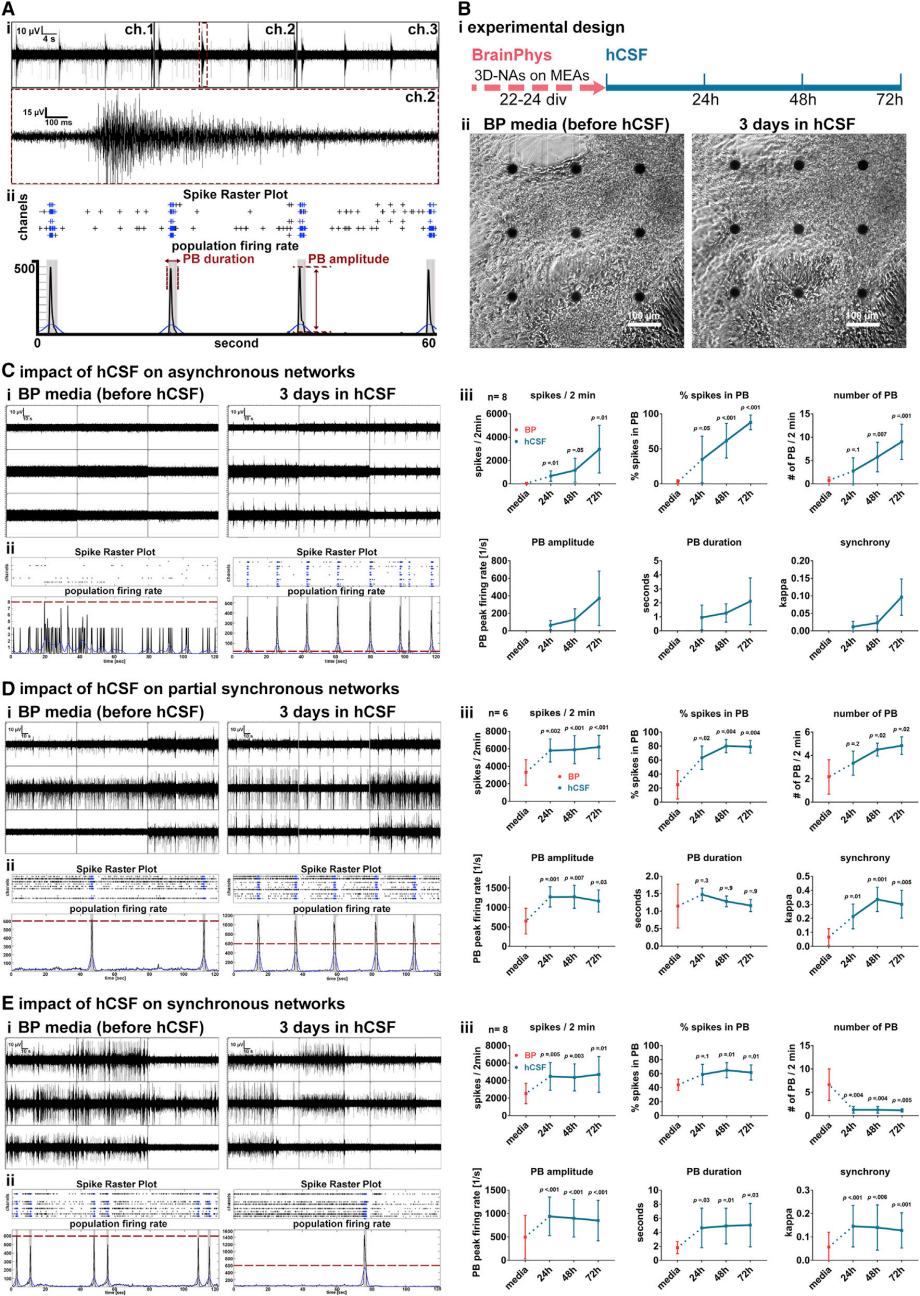
Network Activity and Morphology of hiPSC-Derived Neurons after 72 h Exposure to hCSF (A) (i) Representative example of MEA recording showing the appearance and properties of synchronous activity (three channels). The inset represents a detailed visualization of a population burst (PB) detected by one electrode. (ii) Example of spike raster plot and population firing rate illustrates synchronous network activity. (B) (i) Schematic drawing shows the experimental design. (ii) Phase-contrast images show the morphology of cultures on a nine-electrode array of a six-well MEA, cultured in BP (left) and after 72 h cultivation in hCSF (right). (C–E) Representative examples of (i) MEA recordings, (ii) spike raster plots and population firing rates illustrate the activity of asynchronously active (C), partial synchronously active (D), and synchronously active (E) neuronal populations cultured in BP before and after (72 h) exposure to hCSF. (C–E, iii) Diagrams illustrate the change of neuronal network parameters after the application of hCSF to initially asynchronously active (C, iii) (n = 8, N = 2), partial synchronously active (D, iii) (n = 6, N = 2), and synchronously active (E, iv) (n = 8, N = 2) neuronal populations. Data presented as average values ± SD. Matched one-way ANOVA with Dunnett correction (baseline compared with indicated group) and Tukey correction (comparison between groups) were applied to calculate indicated p values.

First, we studied whether neuronal network activity improved when 3D NAs were cultured in hCSF. Before application of individual hCSF samples, 3D NAs were cultured on six-well MEAs and were maintained in BP-based medium for 2 to 3 weeks (Figure 1D). According to international consensus protocols (Teunissen et al., 2009), hCSF samples from 13 healthy individuals were collected via lumbar puncture, centrifuged, and aliquots were stored at −80°C. Note that hCSF samples were not pooled. At the day of the experiment, cultivation medium was removed and 100 μL from a thawed and pre-warmed aliquot of an individual hCSF sample was added per well and cultures were kept in the incubator. Before and after hCSF application, we performed qualitative assessment of neuronal network properties by visual inspection of MEA recordings. For offline analyses and quantitative assessment, we used the SPANNER software for spike detection and applied a custom-made MATLAB tool to characterize the number of spikes, Cohen's kappa (measure of spike synchrony), and population burst firing in terms of number, inter-event interval, duration, and peak firing rate (“amplitude") of population bursts (Figure 2A, for more details see Experimental Procedures) (Izsak et al., 2019). By combining qualitative and quantitative MEA datasets, we excluded false-positive detection of population bursts. After changing from culture medium to undiluted individual hCSF, we observed that all hCSF samples caused a progressive increase in neuronal network activity within 72 h in all hCSF-treated 3D NA cultures (n = 22, four hCSF samples from different individuals were applied to five to six neuronal networks each). In detail, asynchronously active 3D NA cultures became highly synchronously active after 72 h cultivation in hCSF (Figure 2C). In a complementary manner, 3D NA cultures with initially partially (Figure 2D) and highly synchronous activity (Figure 2E) under BP-based medium showed a rapid increase of neuronal network activity within 72 h incubation in hCSF. Interestingly, synchronously active networks showed a decrease of population bursts when cultured in hCSF for 3 days (Figure 2E, iii); nevertheless, the amplitudes of population bursts were increased and neuronal activity showed a higher degree of synchrony as assessed by the parameters percentage of spikes organized as population bursts and Cohen's kappa value (Figure 2E, iii). In control cultures, full medium exchange with BP-based medium did not induce an increase of neuronal activity or changes in population burst properties (Figure S2A). However, hCSF treatment of those control cultures caused a progressive increase in neuronal network activity within 72 h (Figure S2A).

To exclude that the handling of hCSF samples, i.e., freezing and thawing of the samples, had an impact on the effect of hCSF on human neuronal network function described here, we tested freshly collected hCSF on 3D NA cultures on six-well MEAs. Forty minutes after CSF isolation, pre-warmed hCSF samples were applied on 3D NAs, which showed asynchronous, partially synchronous, or highly synchronous activity when cultured in BP-based culture medium. Identical to frozen samples (Figure 2), the fresh hCSF samples induced synchronous activity in all cultures and led to increased neuronal network activity within 72 h after hCSF application (Figure S2B).

### Long-Term Cultures of hCSF-Treated hiPSC-3D NAs Maintain Enhanced Neuronal Network Activity

**Figure 3:**
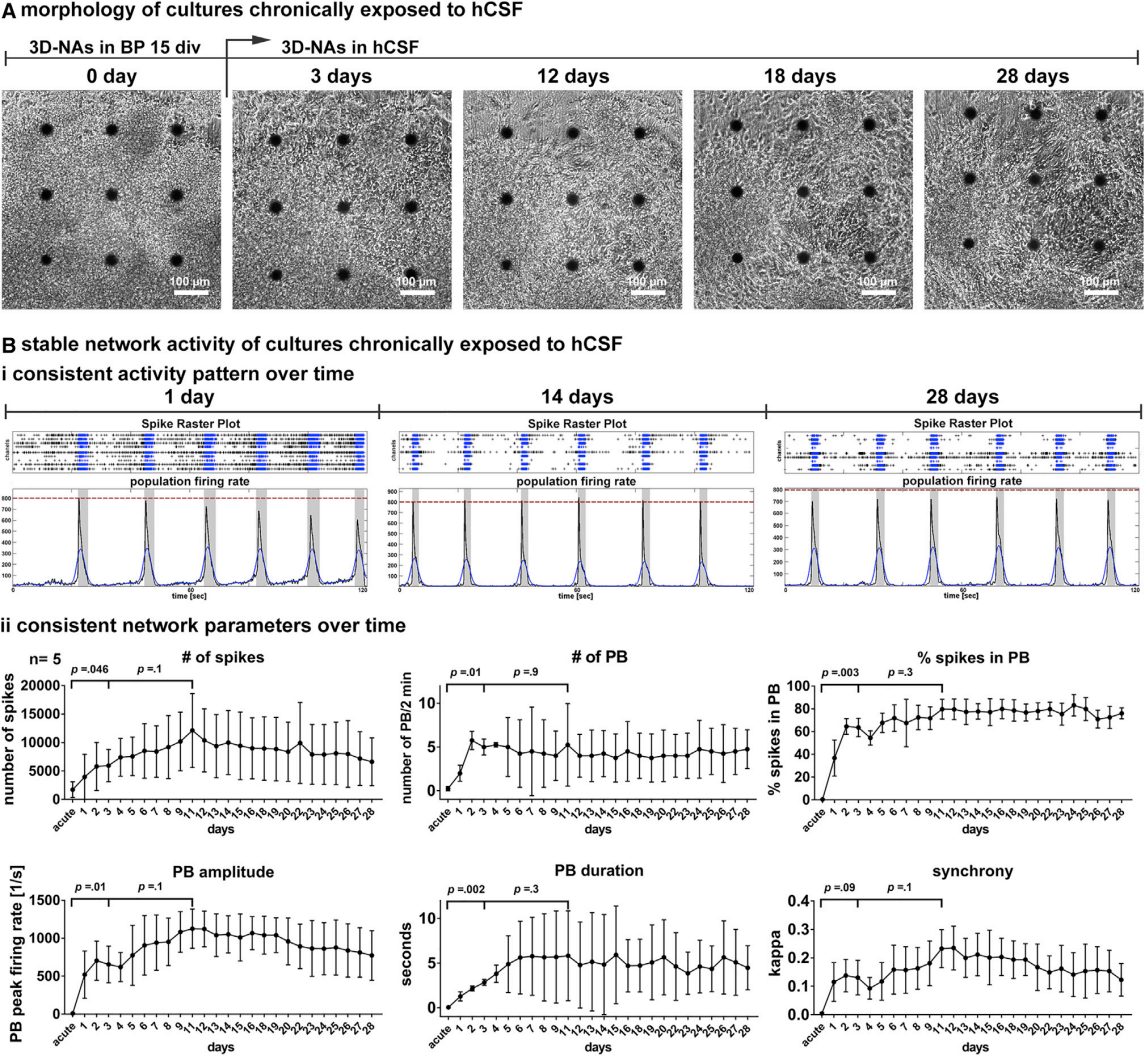
Morphology and Activity of hiPSC-Derived Neural Networks Cultured for 28 Days in hCSF (A) Phase-contrast images show the morphology of cultures exposed to hCSF over time. (B, i) Spike raster plots and population firing rates illustrate the activity of synchronous networks exposed to hCSF after 1, 14, and 28 days, respectively. (B, ii) Diagrams illustrate network parameters after the application of hCSF over time (n = 5, N = 1). Data presented as average values ± SD. Matched one-way ANOVA with Dunnett correction (baseline compared with indicated group) and Tukey correction (comparison between groups) were applied to calculate indicated p values. PB, population burst

Next, we aimed to prove the feasibility of culturing 3D NAs in hCSF over a long time period and evaluated if hCSF-induced improved network activity further increased over time. For this purpose, we cultured hiPSC-derived networks for 4 weeks in hCSF and performed MEA recordings every day (n = 5, three hCSF samples were applied to one to two networks each). After hCSF treatment, 3D NA cultures showed robust attachment to MEA recording electrode array fields, and no detachment of 3D NAs was observed (Figure 3A). As described previously, hCSF caused a rapid increase of neuronal network activity within the first 3 days after hCSF application (Figure 3B). From day 3 to 11 after hCSF application, an increase in some neuronal network parameters could be observed; however, this was not significant. After 11 days, no neuronal parameters showed further changes, indicating a stable plateau phase (Figure 3B, ii) that lasted for the following 4-week recording period (Figure 3B, ii). Interestingly, population burst patterns of 3D NAs cultured for 14 and 28 days in hCSF were nearly identical (Figure 3B, i). By performing additional experiments using two additional hiPSC cell lines, we confirmed enhanced and stable neuronal network activity in 3D NA cultures under this long-term cultivation paradigm (Figure S3).

### Short-Term Application of hCSF Causes Long-Lasting Improved Neuronal Network Activity in hiPSC-3D NAs

**Figure 4:**
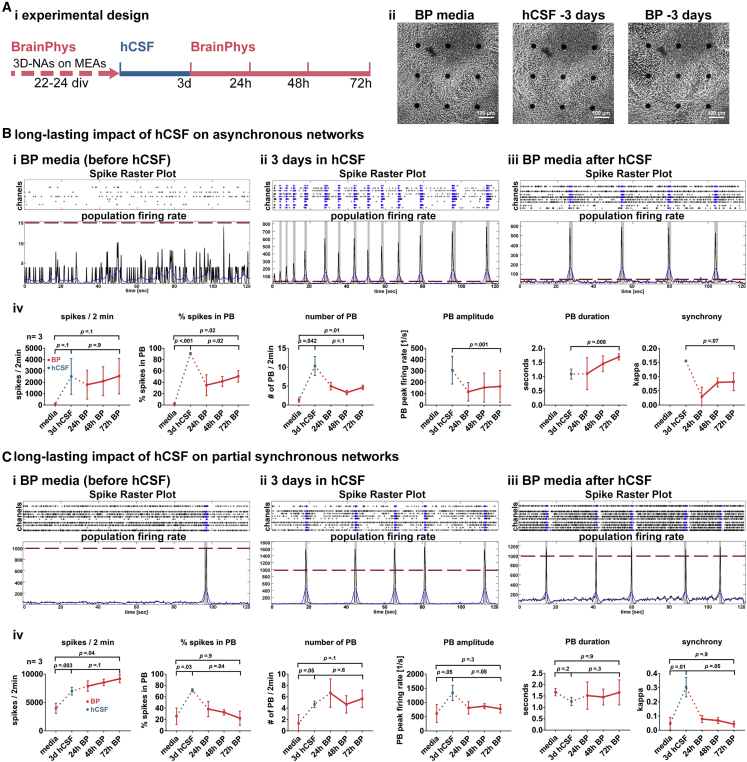
Long-Lasting Functional Alterations of hiPSC-Derived Neural Networks after Exposure to hCSF (A) (i) Schematic drawing illustrates the experimental design. (ii) Phase-contrast images show the morphology of cultures on a six-well MEA, cultured in BP (left), exposed for 72 h to hCSF (middle), and re-exposed to BP for 72 h (right). (B) Examples of spike raster plots and population firing rates illustrate the activity of asynchronous networks (cultured in BP) (i) before, (ii) after (72 h) exposure to hCSF, and (iii) after 72 h re-exposure to BP. (iv) Diagrams illustrate the network activity after the application of hCSF and the long-lasting impact after switching back to BP (n = 3, N = 1). (C) Examples of spike raster plots and population firing rates illustrate the activity of partially synchronous networks (cultured in BP) (i) before, (ii) after (72 h) exposure to hCSF, and (iii) after 72 h re-exposure to BP. (iv) Diagrams illustrate network activity after the application of hCSF and the long-lasting impact after switching back to BP (n = 3, N = 1). Data presented as average values ± SD. Matched one-way ANOVA with Dunnett correction (baseline compared with indicated group) and Tukey correction (comparison between groups) were applied to calculate indicated p values. PB, population burst.

We assessed whether short-term hCSF treatment causes long-lasting changes in neuronal network function of hiPSC neurons. For this purpose, we applied BP-based medium to 3D NA cultures that had been cultured for 72 h in hCSF (Figure 4A, i). As described previously, initially asynchronously and partially synchronously active 3D NA cultures became highly synchronously active within 3 days in hCSF (n = 6, one hCSF sample was applied, Figures 4B and 4C). After 3 days in hCSF, hCSF was replaced by BP-based medium. We observed that the synchronous neuronal network activity remained (Figures 4B and 4C) and showed increased values for nearly all neuronal network parameters (Figures 4B, iv and 4C, iv) after switching back to BP-based medium. However, the level of synchrony and the percentage of spikes in population bursts had a decreasing tendency over time in BP-based medium. Nevertheless, these functional data demonstrate that short-term application of hCSF to 3D NAs causes long-lasting enhanced neuronal network activity of hiPSC neurons.

Complementary applied phase-contrast imaging showed that adherently growing 3D NAs cultured in BP-based cultivation medium move and grow over time (Figure S4A, see also Figure S2 in Izsak et al., 2019). In contrast, 3D NAs cultured in hCSF for 4 weeks neither showed signs of movement or overgrowth, nor detachment of 3D NAs adherently grown on MEAs (Figure S4B).

### hCSF Induces Several Maturation Processes in hiPSC-3D NAs

**Figure 5:**
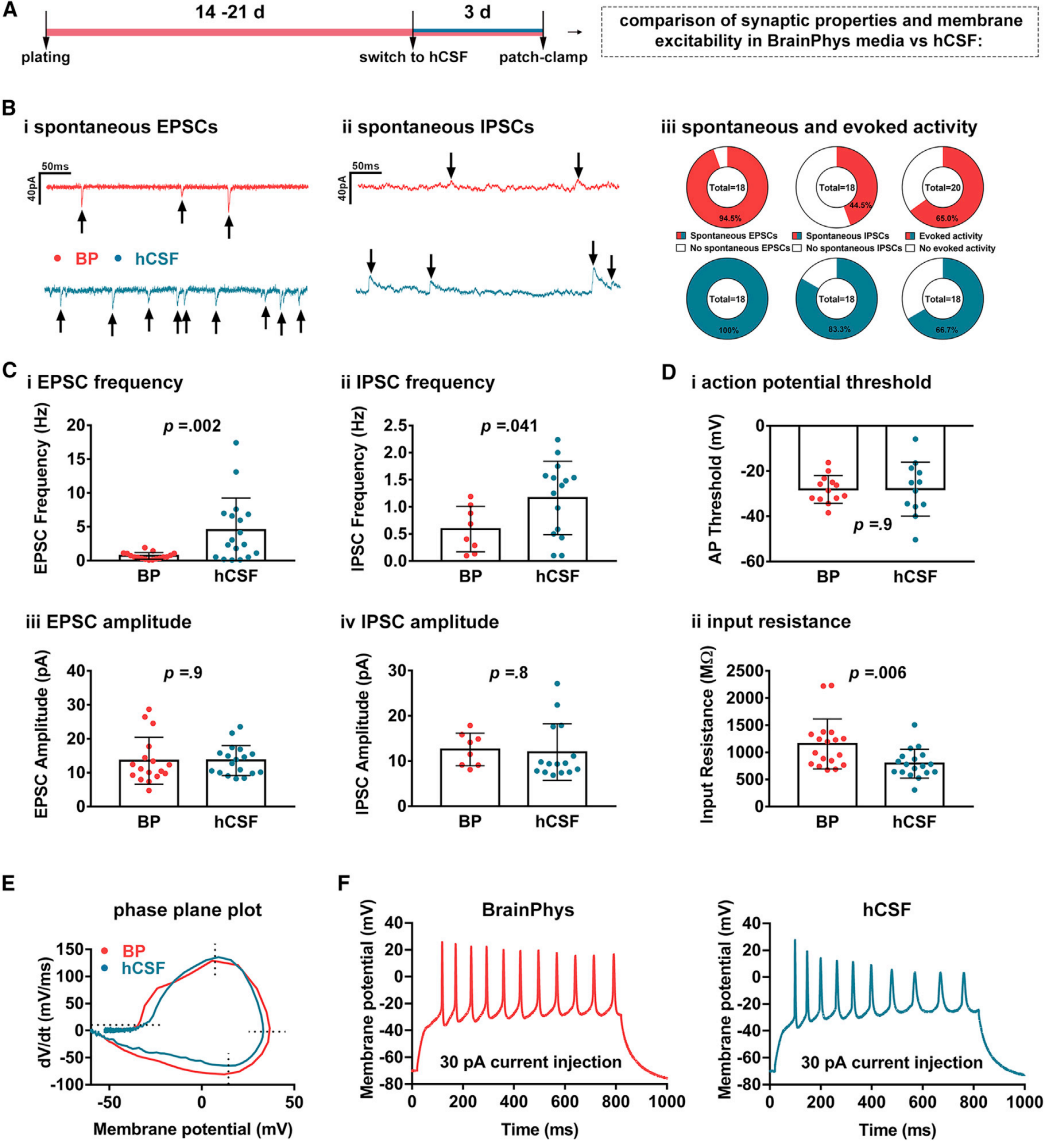
hCSF Triggers Maturation of Synaptic Properties and Connectivity of Individual hiPSC Neurons (A) Schematic drawing illustrating the experimental design. (B) Examples of whole-cell patch-clamp recordings of (i) spontaneous excitatory post-synaptic currents (sEPSCs) and whole-cell patch-clamp recordings of (ii) spontaneous inhibitory post-synaptic currents (sIPSCs) recorded from neurons at the edges of 3D NAs (BP, red or hCSF, blue). Arrows denote active events. Pie charts show the percentage of cells (iii) with sEPSCs, sIPSCs, and evoked activity (in current clamp). Total number of cells is given in the center. (C) Average frequency (i and ii) and amplitude (iii and iv) of sEPSCs and sIPSCs recorded from cells cultured in either BP or hCSF, with individual recordings shown as scattered dots. (D) Average action potential (AP) threshold (i) and input resistance (ii). AP threshold was determined from a phase plane plot at a dV/dt of 10 mV/ms. (E) Examples of phase plane plots. Dotted lines denote AP threshold (at 10 mV/ms), maximal rate of depolarization, amplitude, and maximal rate of repolarization, respectively. (F) Examples of evoked firing responses to an 800-ms current injection of 30 pA into a current-clamped neuron cultured in BP or hCSF. Cells cultured in BP and hCSF were recorded from pairs per experiment. Error bars denote SD and p values were calculated by an unpaired, Student's t test. Data were obtained from two independent experiments.

We applied whole-cell voltage- and current-clamp recordings to assess the passive membrane, excitability and synaptic properties of neurons in 3D NAs that were either kept in BP-based medium or were cultured for 3 days in hCSF samples. The electrophysiological assessment for both groups was performed in artificial cerebrospinal fluid. Neurons at the edges of 3D NAs, in both BP-based medium and hCSF, were excitable and showed spontaneous excitatory ([sEPSCs], BP group: 17 out of 18, hCSF group: 18 out of 18) and inhibitory post-synaptic currents ([sIPSCs], BP group: 8 out of 18, hCSF group: 15 out of 18) (Figure 5B). Three days of hCSF treatment significantly increased the frequency of sEPSCs and sIPSCs (Figure 5C, i, ii) without altering the amplitude of sEPSCs and IPSCs (Figure 5C, iii, iv). A lower input resistance is commonly observed during neuronal maturation *in vitro* and *in vivo* (Ehrlich et al., 2012, Mongiat et al., 2009, Tadros et al., 2015, Takazawa et al., 2012), and it is likely a consequence of a larger and more complex dendritic arbor, which should increase the connectivity. Since the hCSF-treated neurons appear larger, have a lower input resistance (Figure 5D, ii), and show the described substantial increase in spontaneous synaptic activity, we conclude that hCSF-induced neuronal maturation occurs on a single neuronal level.

When injecting currents at stepwise increments with the cell in current clamp, 13 out of 20 and 12 out of 18 neurons showed evoked responses, i.e., action potentials (APs), for BP and hCSF, respectively (Figure 5F; B, iii). From these APs we constructed phase plane plots to calculate the AP threshold (Figure 5E; D, i), the maximal rate of depolarization, amplitude and maximal rate of repolarization. There were no significant differences when comparing AP threshold (−28.12 ± 6.15 versus −28.02 ± 11.96 mV) AP amplitude (62.0 ± 16.1 versus 69.6 ± 14.1 mV), AP half-width (1.7 ± 0.5 versus 2.1 ± 0.7 ms), rheobase (49.3 ± 17.5 versus 43.3 ± 11.5 pA), and maximal rate of depolarization (119.3 ± 91.4 versus 117.9 ± 47.7 mV/ms), repolarization (−51.5 ± 31.8 versus −52.7 ± 20.2 mV/ms), for neurons grown in BP and hCSF, respectively.

Next, we applied immunofluorescent staining and fluorescent confocal imaging to obtain a more detailed morphological and cellular insight into the properties of neural cells in 3D NAs cultured in hCSF or BP-based medium.

**Figure 6:**
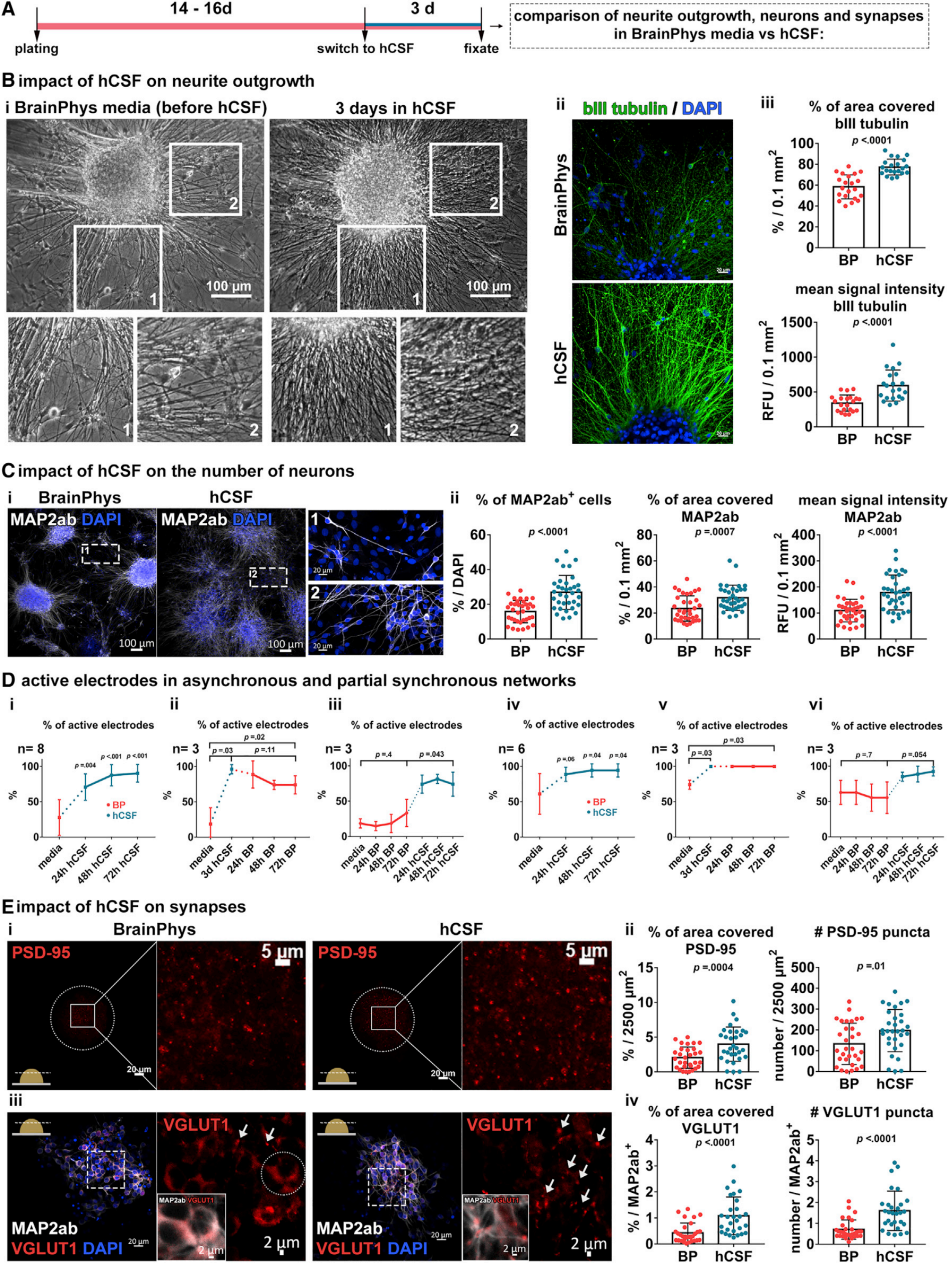
hCSF Triggers Rapid Neuronal Differentiation and Synapse Development (A) Schematic drawing illustrating the experimental design. (B) (i) Phase-contrast images show the morphology of cultures in BP (left) and after exposure for 72 h to hCSF (right), the insets illustrate the marked regions of interest (ROIs) in higher magnification. (ii) Confocal images visualize βIII-tubulin^+^ neurites from 3D NAs cultured in BP (above) and hCSF (below). (iii) Diagrams present quantified parameters for βIII-tubulin^+^ neurite net in the cultures treated with hCSF for 3 days (5 images/coverslip, n = 3, N = 3). (C) (i) Confocal images visualize MAP2AB^+^ neurons in 3D NA cultures under BP (left) and hCSF (right). Detailed images of MAP2AB^+^ neurons outside the 3D NAs. (ii) Diagrams present quantified parameters for MAP2ab^+^ neurons (5 images/coverslip, n = 2–4, N = 3). (D) Diagrams show the percentage of recording MEA electrodes detecting neuronal activity in asynchronous (i, ii, iii) and partial synchronous networks (iv, v, vi) (n indicates number of wells from N = 2). (E) (i) Confocal images visualize the PSD-95^+-^ synapses in 3D NA cultures with BP (left) or hCSF (right) culture conditions. Circles mark the position of 3D NAs and the boxes represent the ROIs shown in a higher magnification. (ii) Diagrams show the percentage of image area that shows PSD-95 signal and the number of PSD-95 objects (5 images/coverslip, n = 3, N = 3). (iii) Confocal images visualize the VGlut1^+^ synapses along the MAP2AB^+^ neurons in 3D NA cultured with BP (left) or hCSF (right). The dashed box marks the ROI presented in higher magnification on the right. The arrows mark synaptic puncta and the circle shows the cytoplasmatic localization of VGlut1 signal in MAP2AB^+^ neurons cultured in BP. The inlet images show the co-localization of VGlut1 signal with the MAP2AB. (iv) Diagrams present quantified parameters for VGlut1^+^ synaptic puncta per MAP2AB^+^ neuron (5 ROIs/coverslip, n = 3, N = 2). Data presented as average values ± SD. Two-tailed, unpaired t test were applied to calculate indicated p values. For MEA diagrams (D) matched one-way ANOVA with Dunnett correction (baseline compared with indicated group) and Tukey correction (comparison between groups) were applied to calculate indicated p values.

Assessment of the βIII-tubulin^+^ neurite net around 3D NAs cultured for 3 days in hCSF, or in BP-based medium (Figure 6B, ii; Video S1), revealed a much more intense neurite net around individual 3D NAs cultured in hCSF (Figure 6B). Note that the imaging settings (e.g., detector gain, laser intensity, etc.) were the same for both groups. Since we observed a significant increase of the number of MAP-2AB^+^-neurons in the hCSF-treated group (Figure 6C; Video S2), we assessed the number of electrodes that detected neuronal activity. Asynchronously and partially synchronously active 3D NA culture showed a progressive increase of active electrodes, and after 3 days in hCSF nearly all cultures showed electrodes detecting neuronal activity (Figure 6D), which persisted even after removal of hCSF and application of BP-based cultivation medium (Figure 6D, ii, v). However, highly synchronously active cultures did not show such changes (data not shown).

Video S1. z Stack Series and 3D Projection of Confocal Images Show βIII-Tubulin^+^ Neurons and DAPI Nuclei in 3D Neural Aggregates Cultured in BrainPhys-Based Media or hCSF, Related to Figure 6

Video S2. z Stack Series and 3D Projection of Confocal Images Show MAP2AB^+^ Neurons and DAPI Nuclei in 3D Neural Aggregates Cultured in BrainPhys-Based Media or hCSF, Related to Figure 6

Next, we assessed the number of PSD-95^+^ post-synapses and VGlut1^+^ pre-synaptic structures within 3D NAs (Figure 6E). Co-immunocytochemistry and confocal imaging showed that the post-synaptic protein PSD-95 was associated with pre-synaptic protein synapsin indicative of mature synapses (Figure S5). We observed that MAP2AB^+^ neurons cultured in BP-based medium showed rather a cytoplasmic and less a dot-like synaptic VGlut1 sub-cellular localization (Figure 6E, iii, left). In contrast, MAP2AB^+^ neurons kept for 3 days in hCSF showed rather a dot-like synaptic and less a cytoplasmic VGlut1 sub-cellular localization (Figure 6E, iii, right). Note that cytoplasmic localization of VGlut proteins occurs in immature neurons and that the translocation of VGlut proteins into the synapse is associated with neuronal maturation and synapse formation (Illes et al., 2009, Real et al., 2006). The image area was chosen in relation to the individual size of the 3D NAs to achieve a reliable quantitative assessment of the PSD-95 and VGlut1 signals in different sized 3D NAs (for details, see Supplemental Experimental Procedures). By this approach, we revealed a significant increase of PSD-95^+^ post-synapses and VGlut1^+^ pre-synapses caused by 3 days hCSF treatment of 3D NAs (Figure 6E, ii, iv).

These electrophysiological and imaging data demonstrate that hCSF induces a rapid increase of the number of electrophysiological active neurons, triggers synapse development, neuronal maturation, and the formation of a dense neurite net.

**Figure 7:**
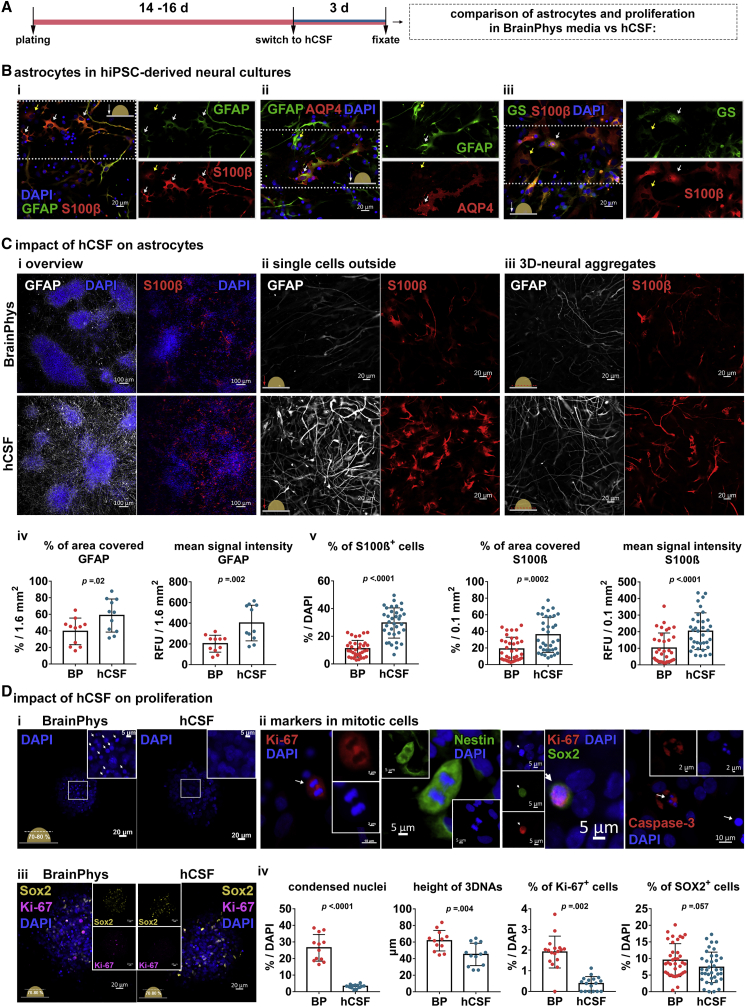
hCSF Triggers Rapid Astrocyte Development and Reduction of Proliferation (A) Schematic drawing of the experimental design. (B) Confocal images visualize astrocytic markers in hiPSC-derived neural cultures: (i) S100β^+^ astrocytes that co-express GFAP (white arrows) or only S100β (yellow arrow), (ii) GFAP^+^ astrocytes that co-express aquaporin (white arrow) or only GFAP (yellow arrow), (iii) S100β^+^ astrocytes that co-express glutamine synthetase (white arrow) or only S100β (yellow arrow). (C) (i) Confocal images visualize GFAP^+^ and S100β^+^ astrocytes in 3D NA cultures under BP (above) and hCSF (below). (ii) Detailed images of GFAP^+^ and S100β^+^ astrocytes outside and (iii) inside of aggregates. Note, images for GFAP and S100β were taken with identical image settings for both groups. (iv) Diagrams present quantified parameters of GFAP^+^ cells (1 image/culture, n = 2–3, N = 3). (v) Diagrams present quantified parameters of S100β^+^ astrocytes (5 images/coverslip, n = 2–3, N = 3). (D) Confocal images visualize the DAPI^+^ nuclei in 3D NA cultured with BP (left) or hCSF (right). Boxes mark the ROIs shown in higher magnification, arrows indicate the presence of condensed nuclei in BP. (ii) Confocal images show the presence of DAPI-condensed DNA in nuclei of Ki-67^+^, Nestin^+^, Sox2^+^ cells. Note, caspase-3^+^ apoptotic cells have fragmented DAPI^+^ DNA and casapase-3 is absent in DAPI-condensed DNA (arrow). (iii) Confocal images visualize Ki-67^+^/Sox2^+^ and Ki-67^−^/Sox2^+^ NSCs in 3D NAs cultured with BP (left) or hCSF (right). (iv) Diagrams present quantified parameters of DAPI^+^-condensed nuclei, height of 3D NAs, Ki-67^+^ proliferating cells, and Sox2^+^ NSCs. Data are presented as mean ± SD. Two-tailed, unpaired t tests were applied to calculate indicated p values.

Next, we performed GFAP stainings to visualize prospective astroglia cells within and around 3D NAs (Figure 7C). After 3 days of treatment with hCSF, 3D NAs showed numerous GFAP^+^ cells outside (Figure 7C, ii) and inside of 3D NAs (Figure 7C, iii), which were very rarely present in 3D NAs cultured in BP-based culture medium (Figure 7C). hCSF-induced GFAP cells showed elongated shape and had processes spanning throughout an entire 3D NA (Figure 7C). An increase of the detector gain was required to visualize the few GFAP cells in BP-based medium cultured 3D NA cultures, indicating that GFAP cells in BP-based medium have lower expression of GFAP than hCSF-induced GFAP cells (Figure S6). Quantitative assessment of the percentage of area covered by GFAP^+^ cells at the edges of adherently growing 3D NAs showed significantly higher values in the hCSF-treated group (Figure 7C, iv).

We stained for additional astrocyte-specific markers and confirmed that 3D NA cultures contained S100β, aquaporin-4^+^, and glutamine synthase^+^ astrocytes, which either co-expressed GFAP or were GFAP^−^ (Figure 7B). We observed that hCSF-cultured 3D NAs showed numerous S100β^+^ astrocytes outside (Figure 7C, i, ii) and inside of 3D NAs, which were less present in 3D NAs cultured in BP-based culture medium (Figure 7C, iii; Videos S3 and S4). Since quantification confirmed that hCSF substantially increased the number of S100β^+^ astrocytes (Figure 7C, v), the data demonstrate that hCSF causes rapid astrocyte development in 3D NAs cultures.

Video S3. 3D Projection of Confocal Images Shows S100beta^+^ Astrocytes and DAPI Nuclei in 3D Neural Aggregates Cultured in BrainPhys-Based Media or hCSF, Related to Figure 7

Video S4. z Stack Series of Confocal Images Shows S100beta^+^ Astrocytes and DAPI Nuclei in 3D Neural Aggregates Cultured in BrainPhys-Based Media or hCSF, Related to Figure 7

Since phase-contrast imaging revealed that 3D NAs cultured in hCSF did not show signs of neural overgrowth of 3D NAs (Figure S4), we applied different markers for proliferating neural stem/progenitor cells. Confocal imaging of DAPI-stained nuclei allows to assess the height of 3D NAs. We observed that hCSF-treated 3D NAs had a significantly lower height than BP medium-treated 3D NAs (Figure 7D, iv; Video S5). In addition, we observed significantly more condensed DNA (DAPI nuclei) in the 3D NAs cultured in BP medium in comparison with hCSF-treated cultures (Figure 7D, i and iv, see also DAPI cells in Videos S1, S2, S3, and S4). By applying the proliferation marker Ki-67, we confirmed that condensed DNA (DAPI nuclei) was present in Ki-67 proliferating cells (Figure 7D, ii). In addition, we observed nuclei with condensed DNA in Nestin^+^ NSCs and Ki-67^+^/SOX-2^+^ NSCs (Figure 7D, ii, iii). In contrast, caspase-3^+^ apoptotic cells have rather a disrupted DAPI-stained DNA (Figure 7D, ii) and we did not observe apoptotic cells with condensed nuclei. We counted the number of Ki-67^+^ proliferating cells and confirmed that a 3-day hCSF treatment of 3D NA cultures resulted in significant reduction of proliferating cells (Figure 7D, iv). Interestingly, the number of SOX-2 NSCs in hCSF-treated cultures was slightly, but not significantly reduced (Figure 7D, iv).

Video S5. Color-Coded Depth Visualization Shows the Different Heights of 3D Neural Aggregates Cultured in BrainPhys-Based Media or hCSF, Related to Figure 7

## Discussion

In an hiPSC-derived 3D NA *in vitro* model (referred to as 3D NA), we applied hCSF from healthy adult individuals to evaluate, if a physiologically relevant and adult brain-like milieu triggers neural maturation processes and enhances neuronal functionality in 3D NAs with immature properties. We demonstrated that hCSF induces several maturation processes, including neurogenesis, electrophysiological maturation of neurons, gliogenesis, and synapse and neurite net formation, which ultimately leads to a rapid formation of synchronously active neuronal circuits in 3D NA cultures. Since patch-clamp and MEA recordings demonstrate the improved electrophysiological function of neurons and enhanced neuronal circuit activity, which persists after removal of hCSF, we conclude that hCSF-induced maturation processes are responsible for this enhanced functionality on single neuronal and neuronal circuit level.

### Implications for hiPSC-Based Neural Development and Neuronal Circuits in *In Vitro* Models

Astrocytes promote synapse formation, neurite growth, and neuronal electrophysiological function (Halassa et al., 2007, Johnson et al., 2007, Tang et al., 2013). Here, we present that the increased number of neurons, a denser neurite net, increased number of PSD-95 and VGlut1 synapses, as well as enhanced individual neuronal electrophysiological and neuronal circuit function correlates with an increased number of S100β astrocytes. Thus, we surmise that, yet unknown, hCSF molecules and hCSF-induced astrocytes caused the enhanced synapse development and supported neurite growth resulting in a human neuronal population with increased functional connectivity and synchronous neuronal activity.

Several studies have shown that the differentiation of hiPSCs into human astrocytes requires 3 to 6 months (e.g., Tcw et al., 2017) and early cultures of hiPSC-derived NSCs have a rather neurogenic than a gliogenic differentiation capacity (e.g., Edri et al., 2015, Gaspard et al., 2008). Tchieu et al. (2019) recently demonstrated that within 5 days NFIA overexpression induced a gliogenic fate in neurogenic hiPSC-derived NSCs. The differentiation of those glia-committed human NSCs required silencing of NFIA and, in addition, 14 days to significantly detect the development of GFAP^+^ astrocytes (Tchieu et al., 2019). In contrast, here we demonstrate that hCSF induced the development of GFAP/S100β astrocytes within 3 days in 3D NAs (56–60 days post iPSC stage). This unexpected and very intriguing observation demonstrates that *in vitro* hiPSC neural cells have the capacity to rapidly adopt an astrocytic phenotype (3 days) *in vitro* when exposed to an appropriate *in vitro* milieu. Since astrocytes represent a heterogeneous group that differs in its capacity to enhance the neuronal properties and astrocyte development requires a diverse set of signaling cues, future studies will help to understand the mechanism of hCSF-induced astrocyte development in human NSCs and to identify novel human gliogenic signaling cues.

As a control condition, we used current, state-of-the art culture medium, the BP medium supplemented with commonly used differentiation factors, e.g., BDNF, GDNF, DAPT. However, these culture conditions are not sufficient to achieve morphological and functional properties in a human 3D neural model as described here by using hCSF. From a neurodevelopmental aspect it is interesting that hCSF not only causes astrocyte development, but induces neuronal differentiation, neuronal maturation, and suppresses proliferation. Thus, adult hCSF contains currently unknown factors that might be necessary to promote neurogenesis and suppression of proliferation to achieve terminal neural maturation in other 3D human neural *in vitro* models, e.g., brain organoids.

In previous studies, we and others showed that hCSF is superior to artificial cerebrospinal fluid and neurobasal-based cultivation medium in promoting electrophysiological function of *in vitro* neurons in primary neuronal cultures (Perez-Alcazar et al., 2016), murine embryonic stem cell cultures (Otto et al., 2009), rodent brain slices (Bjorefeldt et al., 2015, Bjorefeldt et al., 2016), and human brain slices (Schwarz et al., 2017, Wickham et al., 2020). However, the morphological and functional supportive impact of hCSF in hiPSC-derived 3D neural *in vitro* models was unknown. A limitation of all previous work, including ours, is the application of hCSF obtained from unhealthy individuals. For instance, pooled CSF samples obtained from hydrocephalus patients were used as “control” CSF in all previous works (Buddensiek et al., 2009, Buddensiek et al., 2010, Gortz et al., 2013, Jantzen et al., 2013, Koch et al., 2019, Otto et al., 2009, Schwarz et al., 2017, Wickham et al., 2020). It has been known for decades that the chemical composition of hydrocephalus CSF differs from healthy CSF (e.g., Del Bigio, 1989), which indicates that these CSF samples might not be suitable to create a healthy, adult brain-like milieu *in vitro*. Thus, a comparison of the impact of healthy and hydrocephalus CSF on hiPSC neural *in vitro* models represents an interesting approach for a future study. Furthermore, so far only frozen hCSF samples have been used for *in vitro* experiments, and it was unknown if the freezing and storage procedure influenced the quality of hCSF in *in vitro* experiments. Here, we demonstrated that the impact on human neuronal circuit formation of fresh, unfrozen hCSF and frozen hCSF is identical. Thus, our present work shows the impact of non-pathological and fresh hCSF on human *in vitro* neuronal circuit development and function.

A common hallmark of immaturity in hiPSC-based *in vitro* neural model systems, including brain organoid cultures (Lancaster et al., 2013), is ongoing proliferation of residing NSCs, leading to 3D neural cell assemblies that grow for several months *in vitro* (Qian et al., 2019). Within the development of the fetal into the adult brain, CSF-derived factors regulate proliferation, quiescence, and differentiation of NSCs, as described by a plethora of literature (for review see Gato et al., 2005, Kalamakis et al., 2019, Obernier and Alvarez-Buylla, 2019, Zappaterra and Lehtinen, 2012). Here, we demonstrate that hiPSC-derived neural cultures exposed to an adult brain-like milieu (here: adult hCSF) show suppressed proliferation in 3D NA cultures within 3 days. Interestingly, the number of SOX-2 NSCs is not significantly reduced by a 3-day hCSF treatment. Within the same time period, hCSF treatment causes a ∼1.5-fold increase in the number of MAP2AB^+^ neurons and an ∼3-fold increase in the number of S100β astrocytes. Furthermore, overgrowth of 3D NAs is absent after hCSF application, and the reduction of mitotic nuclei, e.g., in Nestin^+^ cells, by hCSF is evident. Thus, we conclude that the absence of neural overgrowth in hCSF-treated cultures might not be predominately mediated by differentiation of SOX-2 NSCs, which would have led to a significant reduction of the SOX-2 NSC population. We rather assume that hCSF induces the differentiation of proliferative neuronal and glial progenitor cells into neurons and astrocytes, respectively. Since SOX-2 is present in proliferative and non-proliferating quiescent NSCs (Lugert et al., 2010, Surzenko et al., 2013), we assume that some non-proliferating quiescent SOX-2^+^ NSCs persist even in the presence of very potent differentiation cues as present in hCSF.

Of course, the question about factors within hCSF, which rapidly promote functional human *in vitro* neuronal circuit development, astroglial and neuronal development, neurite growth, synapse formation, and suppression of proliferation, is highly interesting—however, also challenging to answer. It is extremely unlikely that only one single factor is responsible to trigger the different maturational processes described here. Moreover, we can envision that certain factors might promote the transition from NSCs into neurons, while other factors are required to promote astrocyte development, neurite growth, and synapse development. Since we observed hCSF-enhanced glia development, it is reasonable to expect that these glial cells secrete additional factors, which then further promote neurite growth and synapse development of hiPSC-derived neurons. To highlight the complexity of specific factor identification even more, fetal bovine serum (FBS) is commonly used to create a gliogenic milieu *in vitro* (e.g., Tchieu et al., 2019), and thus used to induce astrocyte development of human NSCs. However, FBS strongly suppresses neuronal network activity (Figure S7) (see also Bardy et al., 2015). Thus, our present work represents the rationale for future studies, which aim to identify novel physiological factors for the improvement of neural development and neuronal function in hiPSC neural *in vitro* models.

## Experimental Procedures

### Generation of Human iPSC-3D NAs

hiPSC lines (C1, C2, C3) were cultured and differentiated into cortical NSCs as described elsewhere (Hayashi et al., 2015, Vizlin-Hodzic et al., 2017). Within 10–14 days, hiPSC NSCs formed 3D NAs (Edri et al., 2015, Izsak et al., 2019) and 3D NAs were manually transferred on coverslips or MEAs and cells were kept in BP medium with supplements.

### Multi-electrode Array Recordings

Two to five hiPSC-3D NAs were seeded as a 5-μL drop directly on PDL/laminin-coated electrode arrays of six-well PEDOT-CNT MEAs. After 1 h, BP medium with supplements was added. Half medium exchanges were performed twice a week. Baseline recordings have been performed in BP medium with supplements before the application of either hCSF or fresh culture medium. Data were recorded and analyzed using MC_Rack software (Multi Channel Systems), SPANNER software suite (RESULT Medical), and custom-built MATLAB software (Hedrich et al., 2014, Izsak et al., 2019).

### Whole-Cell Patch-Clamp Recordings and Data Analysis

Five to ten hiPSC-3D NAs were seeded on PDL/laminin-coated coverslips and cultured with BP medium with supplements. Half medium exchanges were performed twice a week, 14–20 days after differentiation. For the experiment, coverslips were mounted under a differential interference microscope (Nikon E600FN) together with a CCD camera (Sony XC-73CE) to visually identify the cells. Cells were perfused (2–3 mL/min) with artificial CSF. The micropipette was filled with an intracellular solution. The data were collected with a sampling frequency of 10 kHz and filtered at 3 kHz using an EPC-9 amplifier (HEKA Elektronik, D-67466 Lambrecht/Pfalz, Germany). Whole-cell recordings were carried out at 32°C and all recordings were performed between the second (14 days) and third weeks (21 days) *in vitro*. Calculations and data analysis were performed in custom-made IGOR Pro 8 (WaveMetrics, Lake Oswego, OR, USA) software.

For further details and details about hCSF sample collection, immunocytochemistry, image acquisition, and analyses, as well as statistical analysis see Supplemental Experimental Procedures.

### Statistical Analysis

For statistical analysis either matched one-way ANOVA with Dunnett correction (baseline compared with indicated group) or Tukey correction (comparison between groups) were applied; two-way, unpaired t test was applied to calculate indicated p values. All presented data show mean value ± SD, n refers to the number of individual cultures treated with hCSF or BP medium and N refers to the number of individual experiments. For statistical analysis, GraphPad Prism 8.0 software was used.

## Authors Contributions

J.I. performed the experiments, analyzed the data, and prepared the figures. H.S. performed patch-clamp experiments. S.T. developed data analysis programs and critically revised the manuscript. E.H. critically revised the manuscript. S.I. conceived the study, performed part of the experiments and wrote the manuscript.

## Conflicts of Interest

S.I. holds a position at Cellectricon. Cellectricon were not involved in the study, and all experiments and data analyses were conducted at the Sahlgrenska Academy at the University of Gothenburg.

S.T. is founder of Result Medical GmbH, Düsseldorf, Germany.

The other authors declare no conflict of interest.

## Supplemental Information

Document S1. Supplemental Experimental Procedures, Figures S1–S7, and Table S1

Document S2. Article plus Supplemental Information
