# Supplementary material for: Human Cerebrospinal Fluid Promotes Neuronal Circuit Maturation of Human Induced Pluripotent Stem Cell-Derived 3D Neural Aggregates
Source: Stem Cell Reports. 2020 Jun 9;14(6):1044–59. doi: 10.1016/j.stemcr.2020.05.006 (PMC7355159; doi:10.1016/j.stemcr.2020.05.006)
Supplement: Document S1. Supplemental Experimental Procedures, Figures S1–S7, and Table S1 [file mmc1.pdf]

Stem Cell Reports, Volume *14*

Supplemental Information

Human Cerebrospinal Fluid Promotes Neuronal Circuit Maturation of Human Induced Pluripotent Stem Cell-Derived 3D Neural Aggregates

Julia Izsak, Henrik Seth, Stephan Theiss, Eric Hanse, and Sebastian Illes

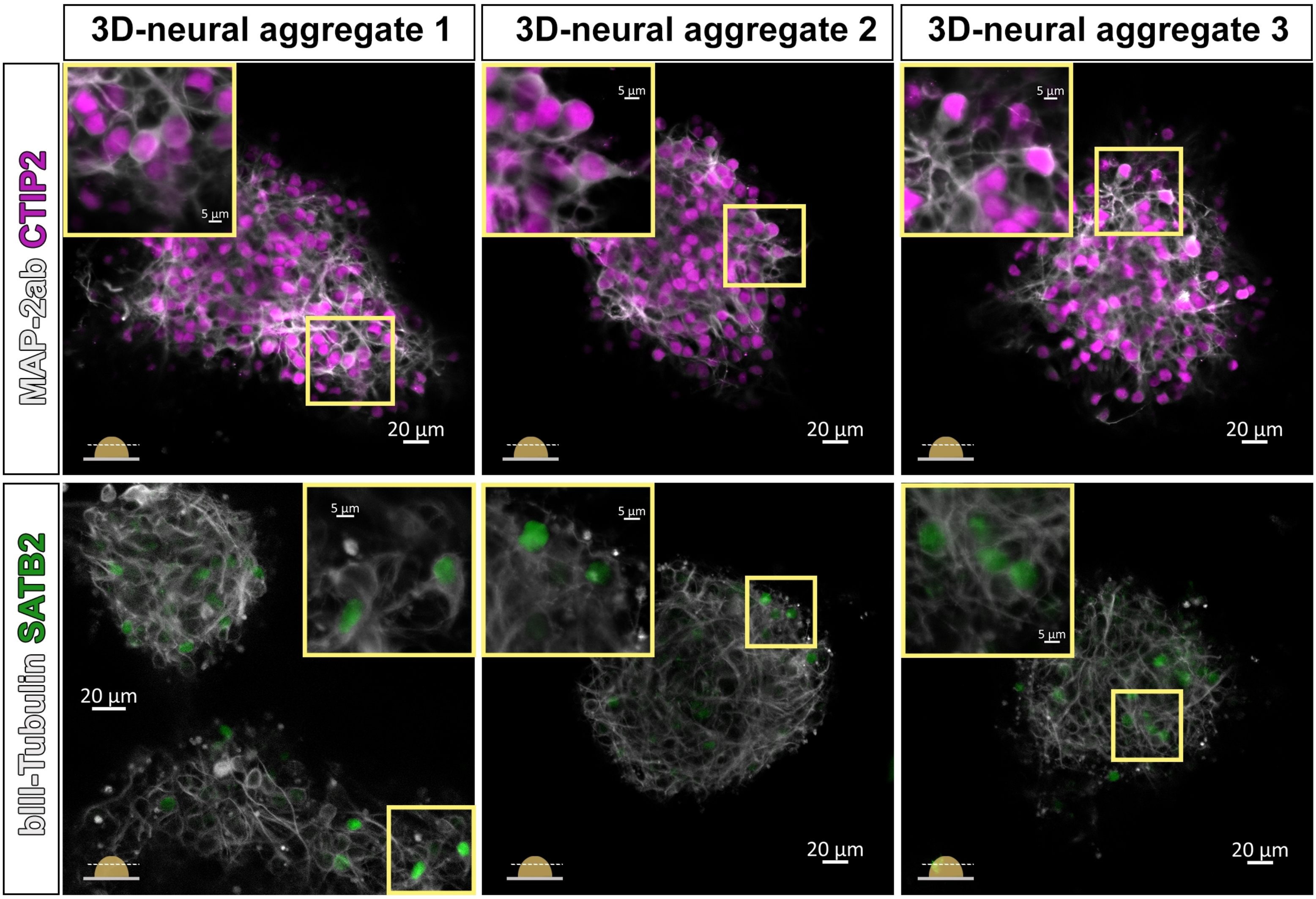
3D-neural aggregate 1
3D-neural aggregate 2
3D-neural aggregate 3
MAP-2ab CTIP2
bIII-Tubulin SATB2
5 µm
20 µm

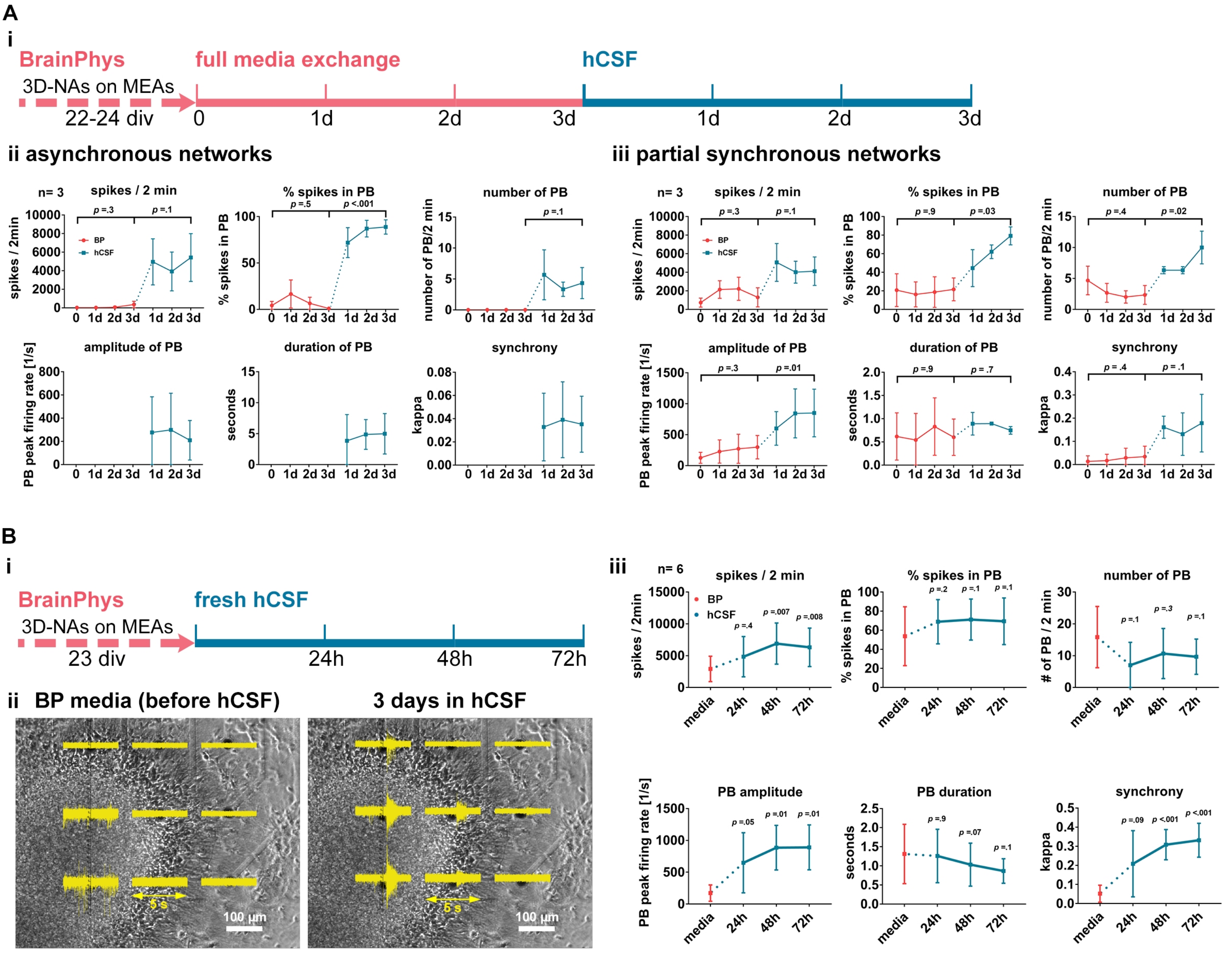
A
i
BrainPhys
full media exchange
hCSF
3D-NAs on MEAs
22-24 div
ii asynchronous networks
iii partial synchronous networks
spikes / 2 min
% spikes in PB
number of PB
amplitude of PB
duration of PB
synchrony
B
i
BrainPhys
fresh hCSF
3D-NAs on MEAs
23 div
ii BP media (before hCSF)
3 days in hCSF
iii
spikes / 2 min
% spikes in PB
number of PB
PB amplitude
PB duration
synchrony

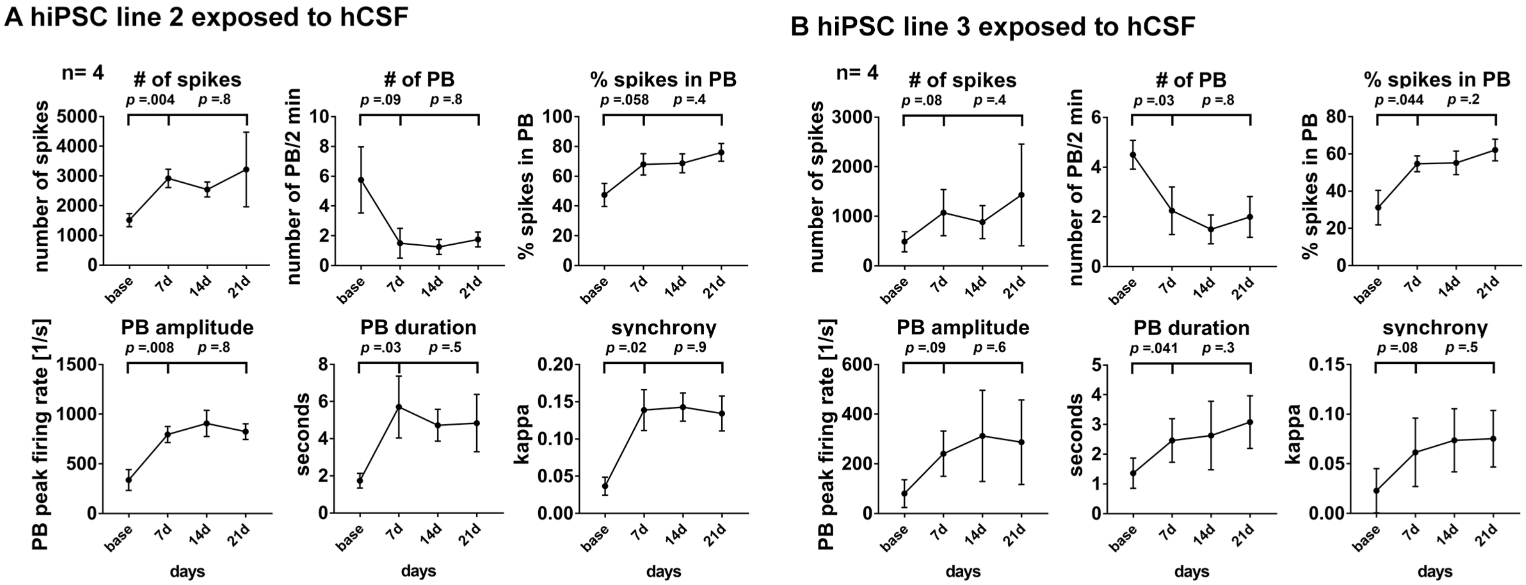
A hiPSC line 2 exposed to hCSF
B hiPSC line 3 exposed to hCSF
n= 4
# of spikes
# of PB
% spikes in PB
PB amplitude
PB duration
synchrony
number of spikes
number of PB/2 min
% spikes in PB
PB peak firing rate [1/s]
seconds
kappa
p =.004
p =.8
p =.09
p =.8
p =.058
p =.4
p =.008
p =.8
p =.03
p =.5
p =.02
p =.9
p =.08
p =.4
p =.03
p =.8
p =.044
p =.2
p =.09
p =.6
p =.041
p =.3
p =.08
p =.5
base
7d
14d
21d
days

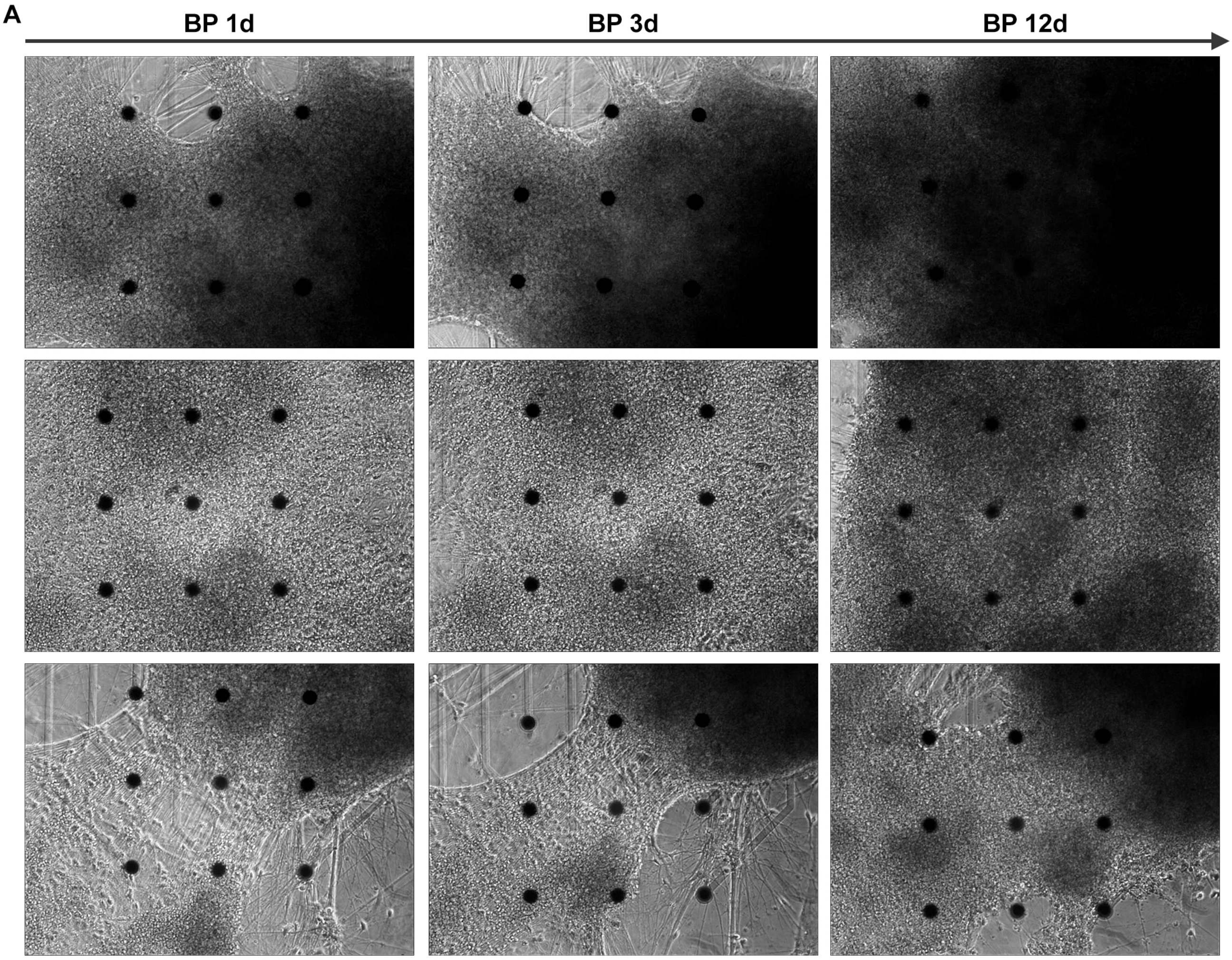
A
BP 1d
BP 3d
BP 12d

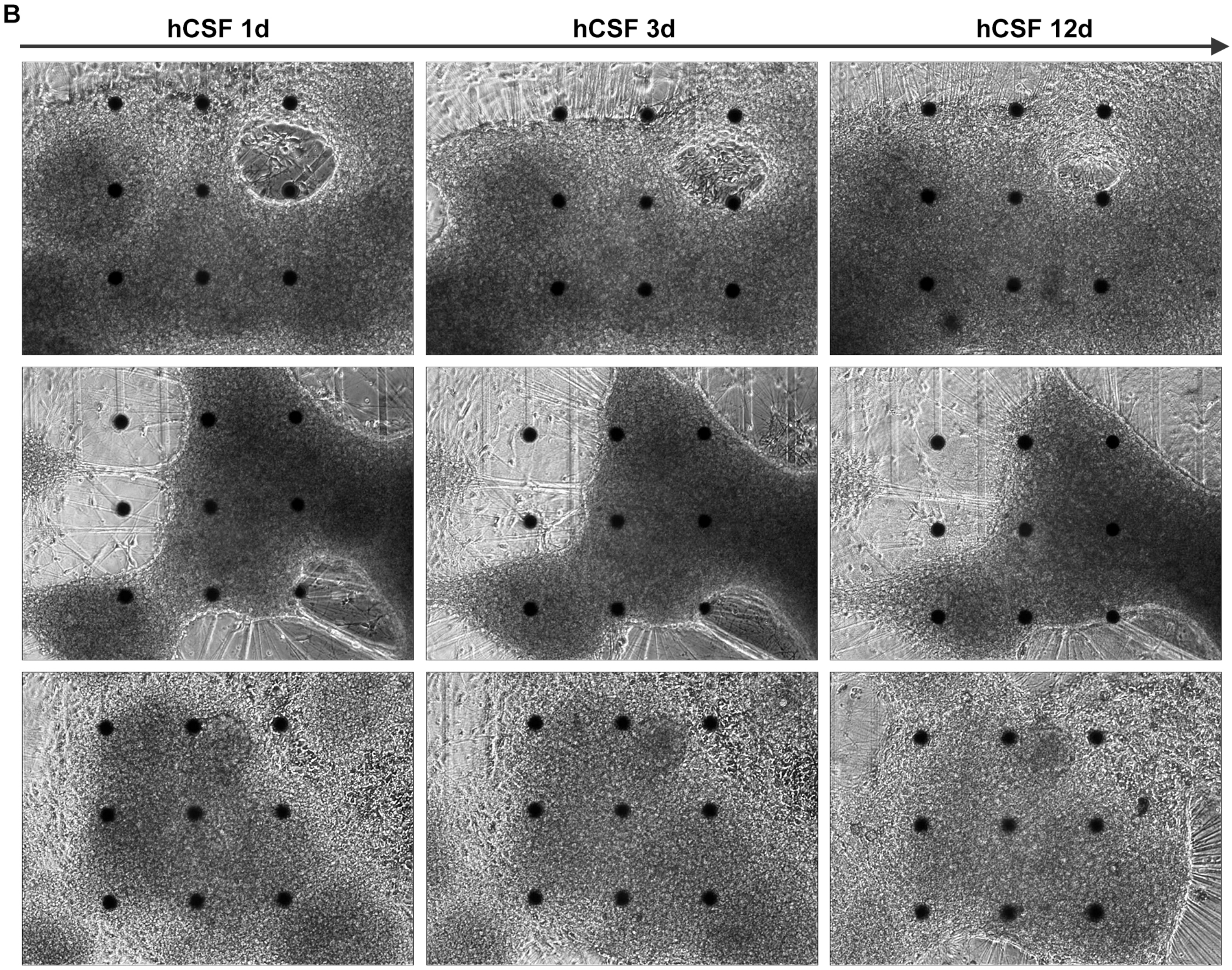
B
hCSF 1d
hCSF 3d
hCSF 12d

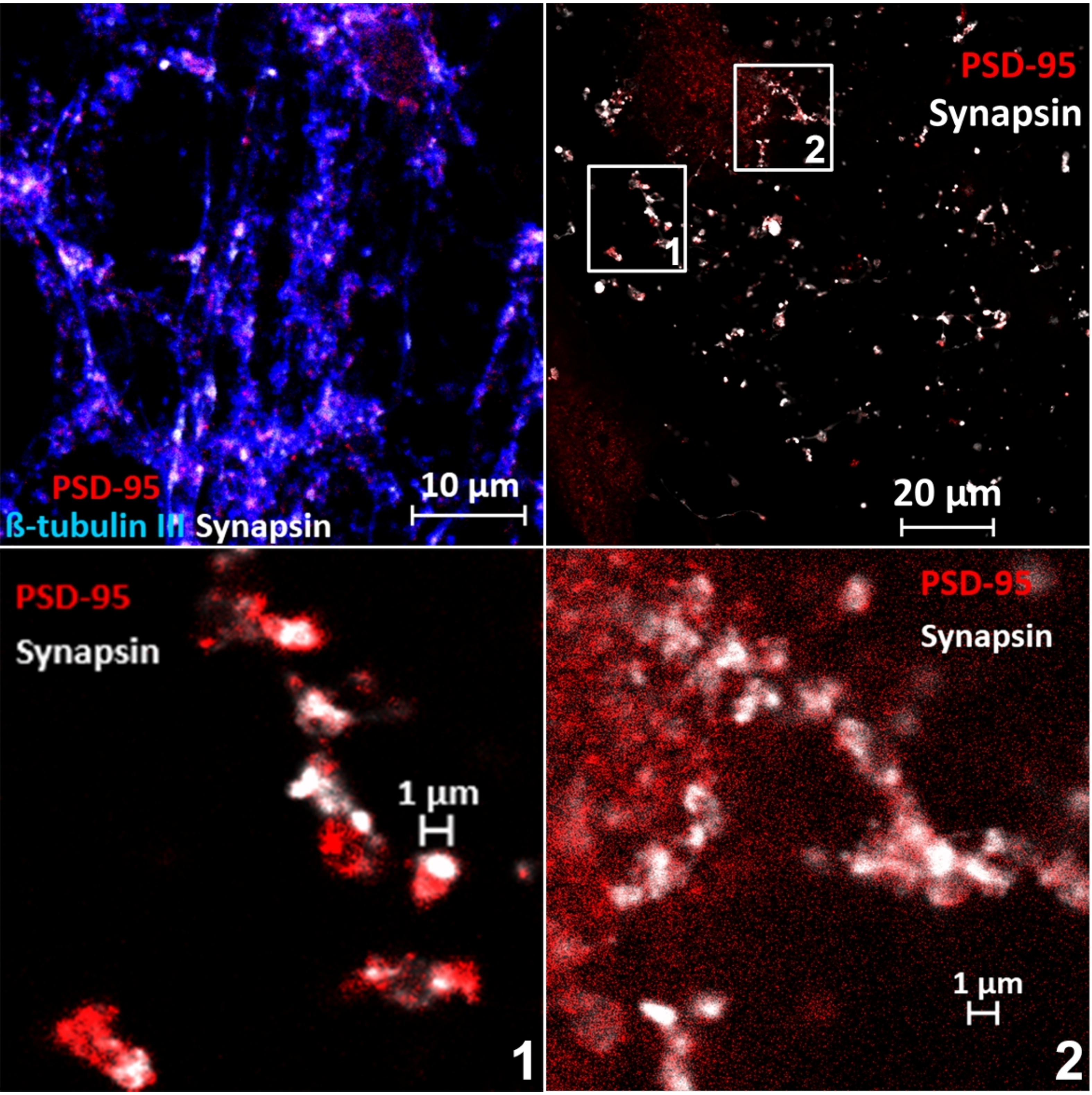
PSD-95
ß-tubulin III Synapsin
10 µm
PSD-95
Synapsin
2
1
20 µm
PSD-95
Synapsin
1 µm
1
PSD-95
Synapsin
1 µm
2

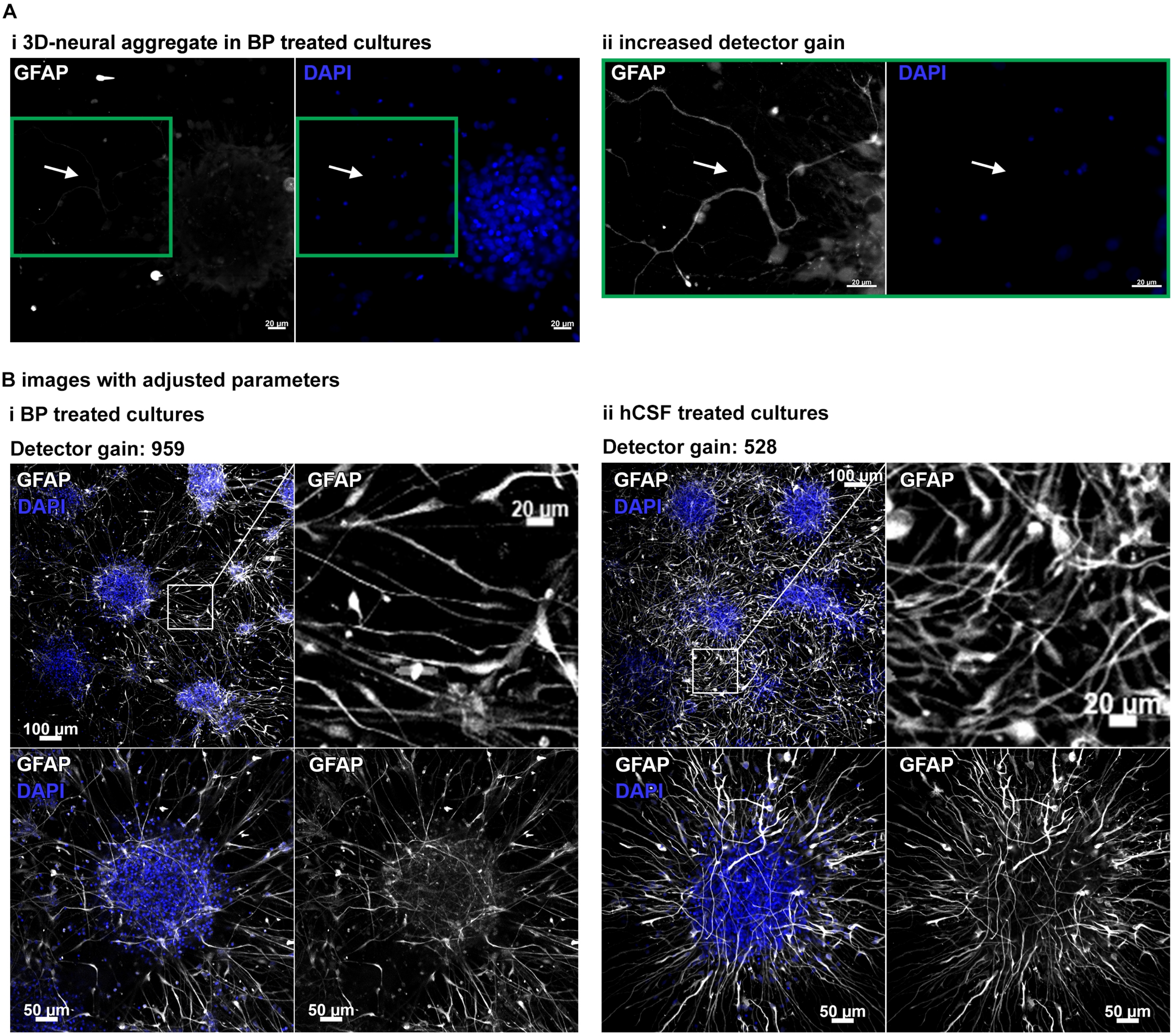
A
i 3D-neural aggregate in BP treated cultures
GFAP
DAPI
20 µm
20 µm
ii increased detector gain
GFAP
DAPI
20 µm
20 µm
B images with adjusted parameters
i BP treated cultures
Detector gain: 959
GFAP
DAPI
100 µm
GFAP
20 µm
GFAP
DAPI
50 µm
GFAP
50 µm
ii hCSF treated cultures
Detector gain: 528
GFAP
DAPI
100 µm
GFAP
20 µm
GFAP
DAPI
50 µm
GFAP
50 µm

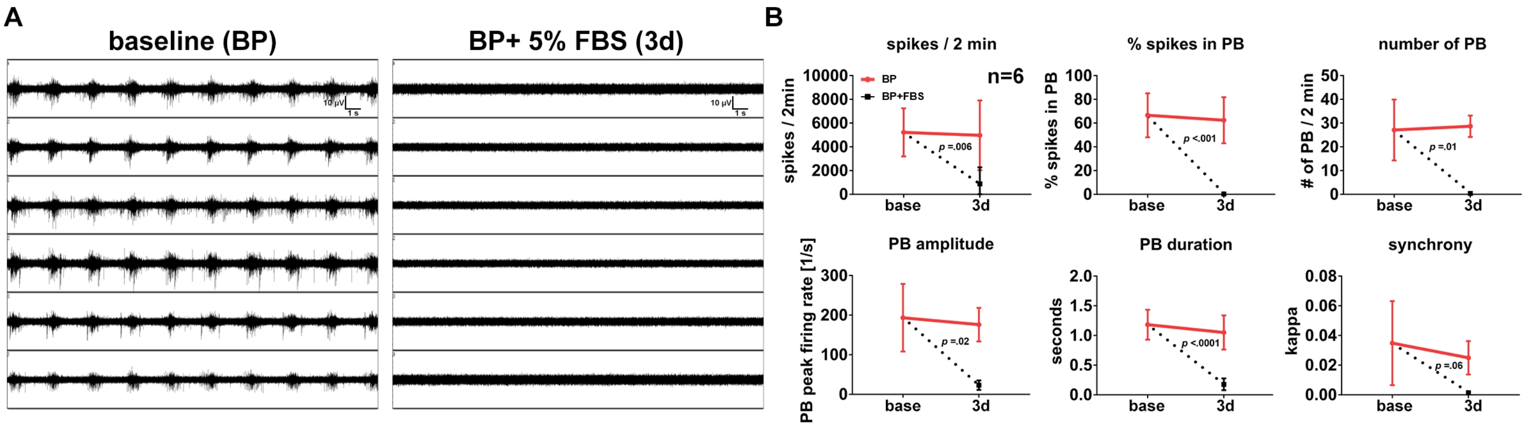
A
baseline (BP)
BP+ 5% FBS (3d)
10 µV
1 s
B
spikes / 2 min
n=6
BP
BP+FBS
spikes / 2min
p =.006
base
3d
% spikes in PB
p <.001
number of PB
# of PB / 2 min
p =.01
PB amplitude
PB peak firing rate [1/s]
p =.02
PB duration
seconds
p <.0001
synchrony
kappa
p =.06

Supplementary material

Title:

Human cerebrospinal fluid promotes neuronal circuit maturation of human induced pluripotent stem cell-derived 3D neural aggregates

Author list:

Julia Izsak[1], Henrik Seth[1], Stephan Theiss[2,3], Eric Hanse[1], Sebastian Illes[1]*

Affiliations:

[1] Institute of Neuroscience and Physiology, Sahlgrenska Academy at University of Gothenburg, Sweden

[2] Institute of Clinical Neuroscience and Medical Psychology, Medical Faculty, Heinrich Heine University, Düsseldorf, Germany

[3] Result Medical GmbH, Düsseldorf, Germany

Contact information:

*Corresponding author: sebastian.illes@neuro.gu.se

Supplemental figure legends

Suppl. Figure 1| Cortical identity of neurons in 3D-neural aggregates

(A) Confocal images show the presence of early-born CTIP2$^+$/MAP2AB$^+$ cortical neurons (up) and late-born SATB2$^+$/bIII-tubulin$^+$-cortical neurons (down) in three different 3D-neural aggregates cultured for 14 days in BrainPhys-based culture media. Boxes mark the ROIs shown in higher magnification. Related to figure 1.

Suppl. figure 2| Control experiments show that full media exchange with BrainPhys-based media does not increase neuronal network activity.

(A) (i) Schematic drawing illustrates the experimental design. Diagrams illustrate the change of neuronal network parameters after full media exchange with BrainPhys-based media and human CSF in (**ii**) asynchronous (n=6) and (**ii**) partially synchronously active neuronal networks (n= 6). Data presented as average values ± standard deviation. **(B) (i)** Schematic drawing shows the experimental design. **(ii)** Phase-contrast images show the morphology of cultures on a nine-electrode array of a six-well MEA, cultured in BrainPhys media (left) and exposed for 72 h to fresh hCSF (right); yellow traces show MEA recordings from the culture. **(iii)** Diagrams illustrate the increase of the network activity after the application of fresh hCSF (n=6). Data presented as average values ± standard deviation. Matched one-way ANOVA with Dunnet correction (baseline compared to indicated group) and Tukey correction (comparison between groups) were applied to calculate indicated *p*-values.Related to figure 2.

Suppl. figure 3| Activity of hiPSC lines 2 and 3 -derived neural networks cultured for 21 days in hCSF.

Diagrams illustrate the increase of network parameters after the application of hCSF and the stable network activity over time in hiPSC line 2 (n=4) **(A)** and hiPSC line 3 (n=4) **(B).** Data presented as average values ± standard deviation. Matched one-way ANOVA with Dunnett correction (baseline compared to indicated group) and Tukey correction (comparison between groups) were applied to calculate indicated p-values. Related to figure 3.

Suppl. figure 4| Morphology of hiPSC 3D neural aggregate cultures exposed to BrainPhys-based media and hCSF.

Phase-contrast images show morphological changes over time of three hiPSC 3D neural aggregate cultures either exposed to **(A)** BrainPhys media or to **(B)** hCSF. Related to figure 3.

Suppl. figure 5| Synapses of hiPSC-derived neurons contain the post-synaptic protein PSD-95 and the pre-synaptic protein Synapsin.

Overview image show the localization of the post-synaptic protein PSD-95 and the pre-synaptic protein Synapsin along ßIII-Tubulin^{+}-neurites and somata from hiPSC-neurons after 21 days cultivation in BrainPhys-based culture media. Detailed confocal imaging demonstrate that PSD-95 and Synapsin puncta appear next to each other. Related to figure 6.

Suppl. figure 6| Visualization of GFAP^{+} astrocytes requires high detector gain in BrainPhys treated cultures

(A) (i) Confocal images shows that the visualization of GFAP^{+}-astroglial cells in 3D-neural aggregate cultures under BrainPhys cannot be achieved by using the same detector gain level as used for hCSF-treated cultures. The inset marks an area which contains a GFAP^{+}-astroglial cell (arrow) that can be visualized by increasing the detector gain (**ii**). **(B)** Confocal images visualize the GFAP^{+}-astroglial cell in 3D-neural aggregate cultures under **(i)** BrainPhys and **(ii)** hCSF, with detailed images showing the outside and inside regions. Note the different applied detector gains to visualize GFAP^{+}-astroglial cell under BrainPhys and hCSF. Related to figure 7.

Suppl. figure 7| Fetal bovine serum suppresses neuronal network activity in hiPSC-derived neural networks

(A) Representative examples of MEA recordings in BrainPhys media left) and after three days exposure to 5% fetal bovine serum (right) **(B)** diagrams illustrate the decrease of network activity after the application of fetal bovine serum. Synchronous networks turn into nearly inactive cultures when exposed to fetal bovine serum. (control: n=6, fetal bovine serum: n=6). Data presented as average values ± standard deviation. *p*-values were calculated by paired student's *t*-test. Related to Figure 2.

Supplementary videos

Note, all suppl. videos contain description about video content.

Suppl. video 1| Z-stack series and 3D projection of confocal images shows bIII-tubulin^{+}-neurons and DAPI-nuclei in 3D-neural aggregates cultured in BrainPhys-based media or hCSF.

Related to figure 6.

Suppl. video 2| Z-stack series and 3D projection of confocal images shows MAP2AB^{+}-neurons and DAPI-nuclei in 3D-neural aggregates cultured in BrainPhys-based media or hCSF.

Related to figure 6.

Suppl. video 3| 3D projection of confocal images shows S100beta^{+}-astrocytes and DAPI-nuclei in 3D-neural aggregates cultured in BrainPhys-based media or hCSF.

Related to figure 7.

Suppl. video 4| Z-stack series of confocal images shows S100beta^{+}-astrocytes and DAPI-nuclei in 3D-neural aggregates cultured in BrainPhys-based media or hCSF.

Related to figure 7.

Suppl. video 5| Color-coded depth visualisation shows the different height of 3D-neural aggregates cultured in BrainPhys-based media or hCSF.

Related to figure 7.

Supplementary table

Suppl. table 1: List of primary and secondary antibodies used for the study

Primary antibody	Supplier (cat. No)	Host	Dilution
GFAP	Pharmingen (556327)	mouse	1:500
S1oo beta	Dako Agilent (Z0311)	rabbit	1:500
Aquaporin 4	Atlas antibodies (HPA014784)	rabbit	1:500
Glutamine synthetase	Chemicon (MAB302)	mouse	1:500
Ki-67	Santa Cruz (sc-15402)	rabbit	1:500
Sox2	R&D (AF2018)	goat	1:500
Map2ab	Abcam (ab11267)	mouse	1:1000
bIII-tubulin	R&D (MAB1195)	mouse	1:2000
PSD-95	Abcam (ab18258)	rabbit	1:1000
Synapsin	Santa Cruz (sc-8295)	goat	1:1000
vGLUT1	Synaptic systems (135303)	rabbit	1:1000
CTIP2	Abcam (ab18465)	rat	1:500
SATB2	Abcam (ab51502)	mouse	1:500
caspase-3	Abcam (ab13847)	rabbit	1:500
Nestin	Merck (MAB353)	mouse	1:500

Secondary antibody	Supplier (cat. No)	Host	Anti-	Dilution
Alexa Fluor 488	Thermo Fisher (A11029)	goat	mouse	1:500
Alexa Fluor 488	Thermo Fisher (A21208)	donkey	rat	1:500
Alexa Fluor 555	Thermo Fisher (A31572)	donkey	rabbit	1:500
Alexa Fluor 488	Thermo Fisher (A11055)	donkey	goat	1:500
Alexa Fluor 405	Thermo Fisher (A31553)	goat	mouse	1:500
Alexa Fluor 555	Thermo Fisher (A21432)	donkey	goat	1:500

Supplementary experimental procedures

Ethical statement

We confirm that all methods were carried out in accordance with relevant guidelines and regulations. We confirm that all experimental protocols were approved by the named institutions. Informed consent was obtained from all subjects. Work with human iPSC lines were approved by a local ethics committee (Regionala etikprövningsnämnden i Göteborg, with the ethical approval number: DNR 172-08. The procedure of CSF sampling and CSF samples application for research purposes was approved by a local ethics committee (Regionala etikprövningsnämnden i Göteborg, with the ethical approval number: DNR 942-12).

Generation of human iPSC-3D neural aggregates

Human iPSC cell lines (C1, C2, C3) were cultured and differentiated into cortical neural stem cells as described elsewhere (Hayashi et al., 2015; Vizlin-Hodzic et al., 2017). Shortly, hiPSCs were cultured under feeder-free conditions in Cellartis DEF-CS™ (Takara Bio Europe AB) or mTesR at 37°C in a humidified atmosphere of 5% CO_2. For neural induction of hiPSCs, the DUAL-SMAD inhibition protocol was applied, as previously established in (Shi et al., 2012). 20-30 days post neural induction, human iPSC-NSC cultures were passaged with Accutase and were frozen in 10%-DMSO solution (solved in DMEM/F12). The cryostocks were preserved at -152°C. For 3D-cortical aggregate formation, frozen cryostocks of hiPSC-NSC were thawed and 1.0×10^6 cells were cultured in neural culture media on laminin [20 µg/ml]-coated 3.5 cm culture plates. Neural culture media consisting of DMEM/F12 GlutaMAX, Neurobasal, 1x N2 supplement, 1x B27 supplement, 5µg ml-1 insulin, 1 mM Ultra glutamine, 100 µM non-essential amino acids, 100 µM 2-mercaptoethanol, 50 U ml-1 penicillin and streptomycin/gentamicin. Within 10-14 days, hiPSC-NSC formed 3D-neural aggregates (Edri et al., 2015; Izsak et al., 2019) and 3D-neural aggregates with diameters ≤150 µM were manually transferred on PDL/laminin-coated coverslips or MEAs. For neuronal differentiation, BrainPhys-media supplemented with N2 supplement, B27 with vitamin A, 2 mM Ultra glutamine, 50 U ml^{-1} Pen/Strep, and 200 µM ascorbic acid were used. Half media exchanges were performed twice a week. To promote neuronal differentiation, DAPT [10µM] and human BNDF, GDNF, TGF-β, were added [20 ng/ml] to the cultivation media.

Multi-electrode array recordings

Two to five hiPSC 3D-neural aggregates were seeded as a 5 µl drop directly on PDL/laminin coated electrode arrays of 6-well PEDOT-CNT-MEAs. After 1 h, BrainPhys media with supplements (described above) was added. Half media exchanges were performed twice a week.

MEAs had a square grid of 9 planar Ti/TiAu electrodes with PEDOT-CNT (carbon nanotube poly-3,4-ethylene-dioxythiophene) of 30 µm diameter and 200 µm spacing. The 9 planar electrodes can record spontaneous activity of neurons in a 50-100 µm radius. Baseline recordings have been performed in BrainPhys media with supplements prior to the application of either HEPES buffered hCSF or fresh culture media. The signals from the 9 electrodes were simultaneously sampled at 25 kHz, and stored using the MC_Rack software provided by Multi Channel Systems. MEA electrodes had an input impedance of 30–50 kΩ according to the specifications of the manufacturer (Multi Channel Systems). Offline-spike detection was performed by the SPANNER software suite (RESULT Medical; see also (Illes et al., 2014)). Synchronous network activity was analyzed by population burst (PB) detection using custom-built Matlab software (Hedrich et al., 2014; Izsak et al., 2019). As described in Hedrich et al, (2014), for the quantification of firing synchrony across pairs of electrodes, spikes were collected in 10-ms-wide bins and subsequently dichotomized to either zero spikes or at least one spike. Consequently, either both electrodes were active during a time bin ("coincident bin"), only one electrode was active, or both were silent. Cohen's κ statistic then captures the proportion of observed coincident bins exceeding the chance expected proportion of coincidences. Similar to Pearson's correlation coefficient, κ values lie in the range −1 to +1. The average κ value of all electrode pairs with a firing rate of at least 30 spikes/min was calculated as a measure of the overall synchrony of a recording.

Whole-cell patch clamp recordings and data analysis

Five to ten hiPSC 3D-neural aggregates were seeded on PDL/laminin coated coverslips and cultured with BrainPhys media with supplements. Half media exchanges were performed twice a week. 14-20 days after differentiation a small plastic cylinder was placed on top of each position and filled with either BrainPhys or hCSF. Subsequently, each coverslip contained two population of cells, one exposed to BrainPhys and one exposed to hCSF.Three days after treatment, patch-clamp recordings were performend. The coverslips were mounted under a differential interference microscope (Nikon E600FN) together with a CCD camera (Sony XC-73CE) to visually identify the cells and to visualize the recording electrode connected to the neuron via a borosilicate glass micropipette (resistance 3-6 MΩ). Cells were perfused (2-3 ml/min) with artificial CSF (aSCF) containing: 1 mM NaH_2PO_4, 123 mM NaCl, 26 mM $NaHCO_3$, 3 mM KCl, 1 mM $MgCl_2$, 2 mM $CaCl_2$, and 10 mM D-glucose. The micropipette was filled with an intracellular solution containing; 127 mM K-gluconate, 8 mM KCl, 10 mM HEPES, 15 mM phosphocreatine, 4 mM Mg-ATP, 0.3 mM Na-GTP (pH ~7.3 and osmolality

280–300 mOsm). Patch-clamp recordings were performed on cells at the edge of 3D-neural aggregates visually identified using infrared differential interference contrast video microscopy. The data was collected with a sampling frequency of 10 kHz and filtered at 3 kHz by an EPC-9 amplifier (HEKA Elektronik, D-67466 Lambrecht/Pfalz, Germany). After opening, the cell was allowed to rest for 5 minutes before recordings started. Series resistance was monitored using a 20 ms 10 mV hyperpolarizing pulse. The series resistance was not allowed to exceed 20 MΩ in whole-cell recordings, or to change more than 20% during an experiment, otherwise the experiment was discarded. Whole-cell recordings were carried out at 32 °C.

The firing response to step-wise current injections (800 ms) was recorded in whole-cell current-clamp and spontaneous synaptic activity (i.e. EPSCs and IPSCs) was recorded in whole-cell voltage-clamp. For spontaneous synaptic activity cells were clamped at -70 mV for recordings of α-amino-3-hydroxy-5-methyl-4-Isoxazolepropionic acid receptor (AMPAR) mediated excitatory postsynaptic currents (EPSCs) and at 0 mV for recordings of γ-aminobutyric acid receptor (GABAR) mediated inhibitory postsynaptic currents (IPSCs). All recordings were performed between second (14 days) and third week (21 days) in vitro.

Spontaneous synaptic activity, i.e. frequency and amplitude, was analyzed in Minianalysis 6.0.3 (Synaptosoft, Fort Lee, NJ, USA). Input resistance was calculated using Ohm's law (U = R x I) after injecting a 50 pA depolarizing current in current-clamp. To calculate the action potential threshold, we constructed phase-plane plots by plotting the first derivative (i.e. the rate of change) of the membrane potential during the first action potential, at current injection, against the membrane potential (Fig. 5E). These plots visualize some aspects of the action potential very clearly (i.e. threshold de- and repolarization as well as amplitude). Threshold was then estimated at a derivative (dV/dt) of 10 mV/ms. Calculations and data analysis were performed in custom-made IGOR Pro 8 (WaveMetrics, Lake Oswego, OR, USA) software.

Human CSF sample collection

Human CSF was collected by lumbar puncture from healthy volunteers (two women and four men, 20-31 years) (Forsberg et al., 2019). The volunteers were recruited via social media and they provided written informed consent. No anesthesia was used. Samples were immediately centrifuged to separate cells from CSF, and 1.5-2 ml aliquots were frozen to -80°C. For further validation of the presented approach, additional de-identified, left-over control hCSF samples (n= six women and three men, 67-89 years old) provided by Sahlgrenska University Hospital, Sweden, were used. The hCSF sampling procedure and classification as control hCSF samples

is according an international consensus protocol (Teunissen et al., 2009). For sampling of fresh hCSF, 12 ml human CSF were obtained via lumbar puncture from a 38-year old male without any neurological symptoms. Additional blood samples were collected at the same time of hCSF sampling. Since IgG level within the blood sample was in the normal range, we exclude acute infection or any other diseases occurring in this human individual. Immediately after lumbar puncture, CSF samples were centrifuged, 11 ml supernatant was collected and stored on ice for 40 min prior direct application to the experiment.

Immunocytochemistry, image acquisition and analyses

For immunocytochemical investigations, cultured hiPSC-3D neural aggregates (control and hCSF treated) were washed in phosphate-buffered saline (PBS), pH 7.2 and fixed for 20 min in 4% paraformaldehyde at room temperature. After fixation, the cells were incubated with 1% BSA for 30 min. Primary antibodies diluted in blocking solution with 0,025 Triton-X were applied at 4°C overnight. After washing in PBS, appropriate secondary antibodies coupled were applied for 2 h at room temperature. Images were collected with a confocal-laser scanning microscope (LSM 700 META Zeiss). All the images were taken with the same image settings per marker. For the overview images and quantification of GFAP+ area coverage, one image per coverslip was taken with an 10x objective. For the rest of the image quantification, five images with a 2 µm optical slice were randomly taken from each coverslip with an 40x water-objective. The raw images were exported as TIFF files by using Zen Blue software. Mean signal intensity values for the different channels were obtained from image acquisition raw data, and further quantification was performed by using the Fiji-plug-in as part of the ImageJ software (Schindelin et al., 2012). The neuronal processes (bIII-tubulin) and coverage by $GFAP^+$ astrocytes were quantified by measuring the percentage of area covered across the image. The number of $MAP2AB^+$ neurons and $S100ß^+$ astrocytes were manually quantified using Cell Counter in ImageJ and normalized per number of total DAPI nuclei. The $DAPI^+$ cell nuclei, the $SOX\text{-}2^+$ and $Ki\text{-}67^+$ nuclei, were quantified by the Nucleus counter plugin (Schneider et al., 2012). For the quantification of $PSD\text{-}95^+$ synapses, 50 µm x 50 µm square ROIs were selected from each image and exported. The PSD-95 particles in the ROIs were counted using the Particle analysis tool implemented in ImageJ (Schneider et al., 2012). For the $vGLUT1^+$ synapses, in order to remove the cytoplasmatic signal, a reduced detector gain was used at image acquisition to visualize only the intense synaptic puncta. The synaptic $vGLUT1^+$ puncta were quantified by the Particle analysis tool and normalized per number of $MAP2ab^+$ neurons. To achieve a reliable quantitative assessment of the PSD-95 and vGlut1 signals in different sized

3D neural aggregates, first measured the total height of 3D neural aggregates by using the DAPI-visualized nuclei as references. Here, we defined that the top of 3D neural aggregates is 100% and the bottom of 3D neural aggregates is 0%. Then we imaged 2 µm thick optical slices at a z-level position that represented 80% of the total height of each individual 3D neural aggregate. In these optical slices, we compared the percentage of area covered by PSD-95^{+} post-synapses and vGlut1^{+} pre-synapses as well as the number of PSD-95^{+} post-synaptic and vGlut1^{+} pre-synaptic dots in BP-based media and three days hCSF treated 3D neural aggregates. For vGlut1, we normalized these values to the number of MAP2AB^{+} neurons counted in the same image field.

All the image quantification was manually revised to exclude false signal detection. The measurement of 3D-NA cluster height was done live by using the z-stack method in the Zen Black image acquisition software. For the z-stack videos of the 3D-neural aggregates 2,5 µm optical slices were taken with an interval of 2,2 µm (15-30 slices per aggregate). The raw and depth-coded z-stack projections were exported with the use of Zen Blue software. The used primary and secondary antibodies are summarized in suppl. table 1.

Statistical analysis

For statistical analysis either matched one-way ANOVA with Dunnet correction (baseline compared to indicated group) and Tukey correction (comparison between groups) were applied or two-way, unpaired *t*-test were applied to calculate indicated *p*-values. All presented data show mean value ± standard deviation (SD), *n* refers to the number of individual cultures treated with hCSF or BrainPhys media and N refers to the number of individual experiments. For statistical analysis, GraphPad Prism 8.0 software was used.
