# Supplementary material for: Human Cerebrospinal Fluid Promotes Neuronal Circuit Maturation of Human Induced Pluripotent Stem Cell-Derived 3D Neural Aggregates
Source: Stem Cell Reports. 2020 Jun 9;14(6):1044–59. doi: 10.1016/j.stemcr.2020.05.006 (PMC7355159; doi:10.1016/j.stemcr.2020.05.006)
Supplement: Document S2. Article plus Supplemental Information [file mmc7.pdf]

Stem Cell Reports
Article
OPEN ACCESS

Human Cerebrospinal Fluid Promotes Neuronal Circuit Maturation of Human Induced Pluripotent Stem Cell-Derived 3D Neural Aggregates

Julia Izsak,[1] Henrik Seth,[1] Stephan Theiss,[2,3] Eric Hanse,[1] and Sebastian Illes[1,*]
[1]Institute of Neuroscience and Physiology, Sahlgrenska Academy at University of Gothenburg, Gothenburg, Sweden
[2]Institute of Clinical Neuroscience and Medical Psychology, Medical Faculty, Heinrich Heine University, Düsseldorf, Germany
[3]Result Medical GmbH, Düsseldorf, Germany
*Correspondence: sebastian.illes@neuro.gu.se
https://doi.org/10.1016/j.stemcr.2020.05.006

SUMMARY

Human induced pluripotent stem cell (hiPSC)-derived *in vitro* neural and organoid models resemble fetal, rather than adult brain properties, indicating that currently applied cultivation media and supplements are insufficient to achieve neural maturation beyond the fetal stage. *In vivo*, cerebrospinal fluid molecules are regulating the transition of the immature fetal human brain into a mature adult brain. By culturing hiPSC-3D neural aggregates in human cerebrospinal fluid (hCSF) obtained from healthy adult individuals, we demonstrate that hCSF rapidly triggers neurogenesis, gliogenesis, synapse formation, neurite outgrowth, suppresses proliferation of residing neural stem cells, and results in the formation of synchronously active neuronal circuits *in vitro* within 3 days. Thus, a physiologically relevant and adult brain-like milieu triggers maturation of hiPSC-3D neural aggregates into highly functional neuronal circuits *in vitro*. The approach presented here opens a new avenue to identify novel physiological factors for the improvement of hiPSC neural *in vitro* models.

INTRODUCTION

Human cerebrospinal fluid (hCSF) flows within the ventricular system, central canal, and subarachnoid space, and surrounds the central nervous system. Molecules within the CSF regulate cellular processes within the embryonic and adult brain (Bachy et al., 2008; Obernier and Alvarez-Buylla, 2019; Zappaterra and Lehtinen, 2012).

Human induced pluripotent stem cell (hiPSC)-derived neural cells are used as *in vitro* models to obtain insights into brain development and human neuronal function. For decades, DMEM and neurobasal culture media have been used as the cell culture environment for human- and animal-derived neurons *in vitro*. Bardy et al. (2015) presented BrainPhys (BP) medium with adjusted major ion concentrations identical to hCSF. Because Bardy et al. showed that electrophysiological function of hiPSC neurons cultured in BP is superior to previously used DMEM and Neurobasal culture medium, BP is currently considered as the most physiologically relevant culture medium for hiPSC-derived neurons *in vitro* (Livesey, 2015). A comparison of neuronal circuit development and function of *in vitro* neurons cultured in BP-based medium or hCSF represents an interesting, however, yet unaddressed approach.

Ongoing proliferation of neural stem cells (NSCs) and limited neuronal maturation are common phenomena described for 2D and 3D hiPSC-derived neural *in vitro* models (Kirwan et al., 2015; Lancaster et al., 2013; Qian et al., 2019). Application of neurotrophic factors (e.g., BDNF, GDNF) alone is insufficient to suppress ongoing proliferation. Application of small molecules, such as DAPT or PD0332991, are used to mediate quiescence of NSCs and neuronal differentiation (Borghese et al., 2010; Kemp et al., 2016; Kirkeby et al., 2012; Rushton et al., 2013). However, the use of small molecules represents rather an artificial approach to trigger maturation in human neural *in vitro* models. Therefore, exposing hiPSC-derived neural cells to hCSF would allow to study the functional and cellular maturation of hiPSC-derived neural cells in a physiologically relevant environment *in vitro*.

In the present study, we analyzed the functional and cellular impact of hCSF on hiPSC-3D neural aggregates (3D NAs) comprised of cortical neurons, glial cells, and residing proliferative neural cells (Edri et al., 2015; Izsak et al., 2019). For this purpose, we applied patch-clamp technique, microelectrode array (MEA) recordings, and confocal imaging on 3D NAs exposed to hCSF obtained from healthy adult individuals. After changing from BP-based medium to hCSF, 3D NAs showed a tremendous increase of neuronal network activity within 3 days, which was not reversible and lasted for several weeks. Immunocytochemical staining and confocal imaging revealed that hCSF caused an immediate increase of neurite net formation, synapse formation, astroglial and neuronal development, as well as suppressed proliferation of residing NSCs in 3D NAs. Our work demonstrates that hCSF improves the maturation of neuronal circuits in a human 3D neural *in vitro* model.

RESULTS

Properties of Adherently Growing hiPSC-3D NA Cultures

We applied the commonly used “dual-SMAD-inhibition” protocol for neural differentiation of hiPSC into cortical

 © 2020 The Author(s).
This is an open access article under the CC BY license (http://creativecommons.org/licenses/by/4.0/).

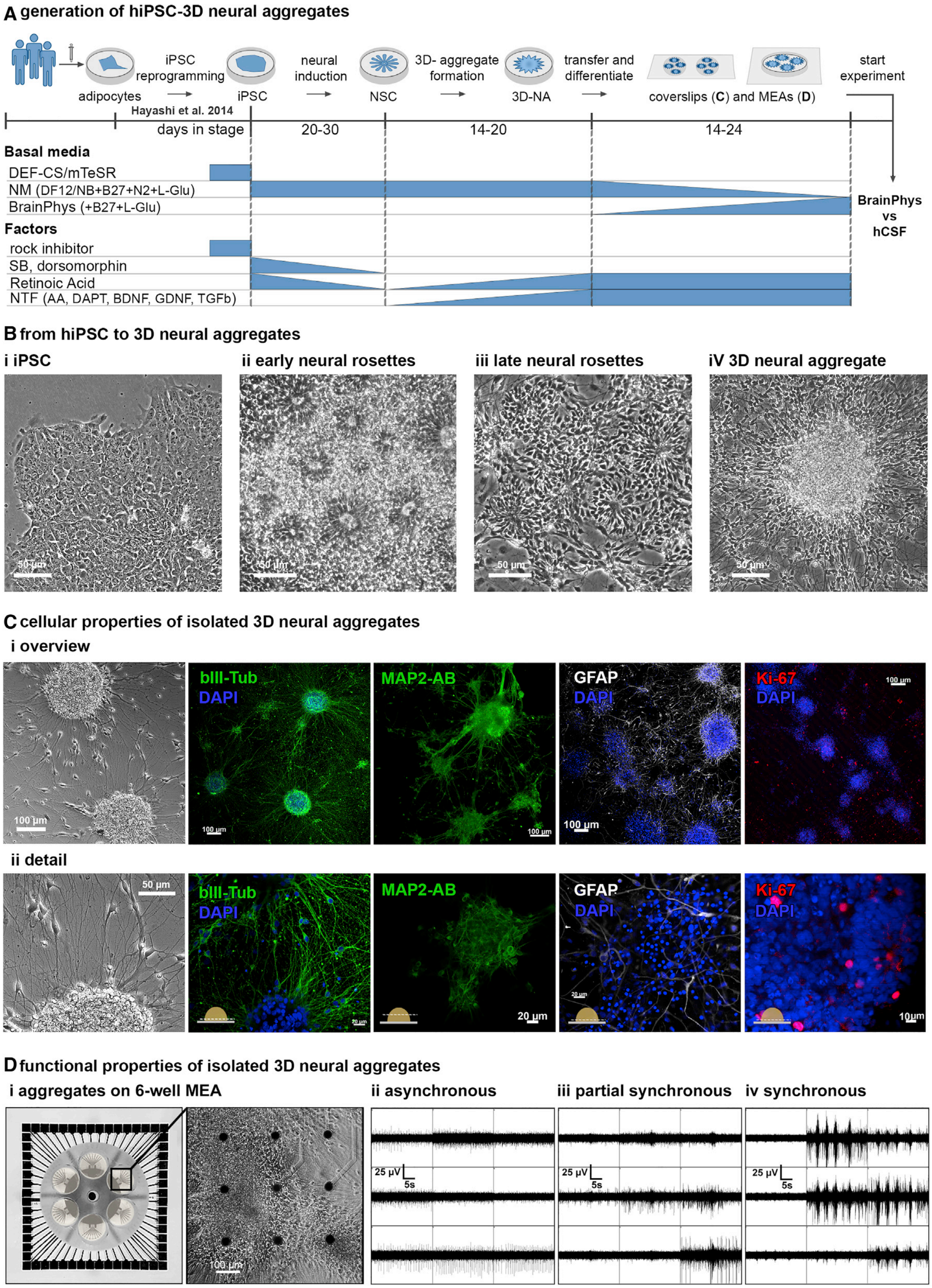

(legend on next page)

NSCs (Izsak et al., 2019; Vizlin-Hodzic et al., 2017) (Figure 1A). hiPSC-derived cortical NSCs grow as neural rosettes (Figure 1B, ii, iii), which give rise to adherently growing 3D NAs (Figure 1B, iv) (Edri et al., 2015; Izsak et al., 2019). For further assessment of cellular and electrophysiological properties, isolated 3D NAs (Figure 1C) were cultured either on glass coverslips or on six-well MEA chips.

Immunocytochemistry and confocal imaging show that adherently growing 3D NAs consist of astroglial cells and cortical neurons (Figures 1C and S1). However, we observed ongoing growth of 3D NAs and the presence of Ki-67 proliferating NSCs (Figure 1C) (Izsak et al., 2019). To describe the development and properties of neuronal network activities generated by neurons in 3D NAs, multi-site extracellular recordings were performed on 3D NAs that adherently grow on nine microelectrodes per well (Figure 1D, i). Each microelectrode allows extracellular recording of spiking and bursting activity generated by neurons in close vicinity (up to 75 μm) to the microelectrode. We demonstrate that neurons within adherently growing 3D NA cultures form functional neuronal circuits that either show asynchronous (Figure 1D, ii), partially synchronous (Figure 1D, iii), or synchronous (Figure 1D, iv) neuronal population activity within 3 weeks in culture. Asynchronous activity is characterized by spontaneous uncorrelated spiking and bursting activity detected by few electrodes and no synchronization of bursts across electrodes (Figure 1D, ii). Partially synchronously active neuronal networks show spontaneous synchronous bursts (Figure 1D, iii), defined as population bursts, which show an irregular pattern. Synchronously active neuronal networks show spontaneous population bursts with a regular firing pattern (Figure 1D, iv).

Since 3D NAs are comprised of highly functional neurons and astroglial cells, however retain immature properties, such as ongoing proliferation, we used adherently growing hiPSC-3D NAs to describe the influence of hCSF on a complex human 3D neural *in vitro* model.

Adherently Growing Human iPSC-3D NAs Cultured in Healthy hCSF Show Increased Synchronous Neuronal Network Activity

First, we studied whether neuronal network activity improved when 3D NAs were cultured in hCSF. Before application of individual hCSF samples, 3D NAs were cultured on six-well MEAs and were maintained in BP-based medium for 2 to 3 weeks (Figure 1D). According to international consensus protocols (Teunissen et al., 2009), hCSF samples from 13 healthy individuals were collected via lumbar puncture, centrifuged, and aliquots were stored at −80°C. Note that hCSF samples were not pooled. At the day of the experiment, cultivation medium was removed and 100 μL from a thawed and pre-warmed aliquot of an individual hCSF sample was added per well and cultures were kept in the incubator. Before and after hCSF application, we performed qualitative assessment of neuronal network properties by visual inspection of MEA recordings. For offline analyses and quantitative assessment, we used the SPANNER software for spike detection and applied a custom-made MATLAB tool to characterize the number of spikes, Cohen's kappa (measure of spike synchrony), and population burst firing in terms of number, inter-event interval, duration, and peak firing rate ("amplitude") of population bursts (Figure 2A, for more details see Experimental Procedures) (Izsak et al., 2019). By combining qualitative and quantitative MEA datasets, we excluded false-positive detection of population bursts. After changing from culture medium to undiluted individual hCSF, we observed that all hCSF samples caused a progressive increase in neuronal network activity within 72 h in all hCSF-treated 3D NA cultures (n = 22, four hCSF samples from different individuals were applied to five to six neuronal networks each). In detail, asynchronously active 3D NA cultures became highly synchronously active after 72 h cultivation in hCSF (Figure 2C). In a complementary manner, 3D NA cultures with initially partially (Figure 2D) and highly synchronous activity (Figure 2E) under BP-based medium showed a rapid increase of neuronal network activity within 72 h incubation in hCSF. Interestingly, synchronously active networks showed a decrease of population bursts when cultured in hCSF for 3 days (Figure 2E, iii); nevertheless, the amplitudes of population bursts were increased and neuronal activity showed a higher degree of synchrony as assessed by the parameters percentage of spikes organized as population bursts and Cohen's kappa value (Figure 2E, iii). In control cultures, full

Figure 1. Generation of hiPSC-Derived 3D NAs
(A) Schematic representation of the *in vitro* generation of hiPSC-3D NAs.
(B) Phase-contrast images show the morphology of hiPSC (i), early neural rosettes (14 days *in vitro* [DIV]) (ii), late neural rosettes (30 DIV) (iii), and 3D NAs (50 DIV) (iv).
(C) Overview (i) and detailed images (ii) of βIII-tubulin$^+$ and MAP2-AB$^+$ neurons, GFAP$^+$ cells, and Ki-67$^+$ proliferating cells within 3D NAs. Schematic drawings in (ii) illustrate z levels of image acquisition within 3D NAs.
(D) Image shows a six-well MEA chip comprising nine microelectrodes per well and phase-contrast image shows cultured 3D NAs (20 DIV) (i). MEA recordings show asynchronous (ii), partial synchronous (iii), and synchronous (iv) neuronal population activities. Each box represents the spiking and bursting activity detected by one electrode. NM, neuronal medium; AA, ascorbic acid; NTF, neurotrophic factors.

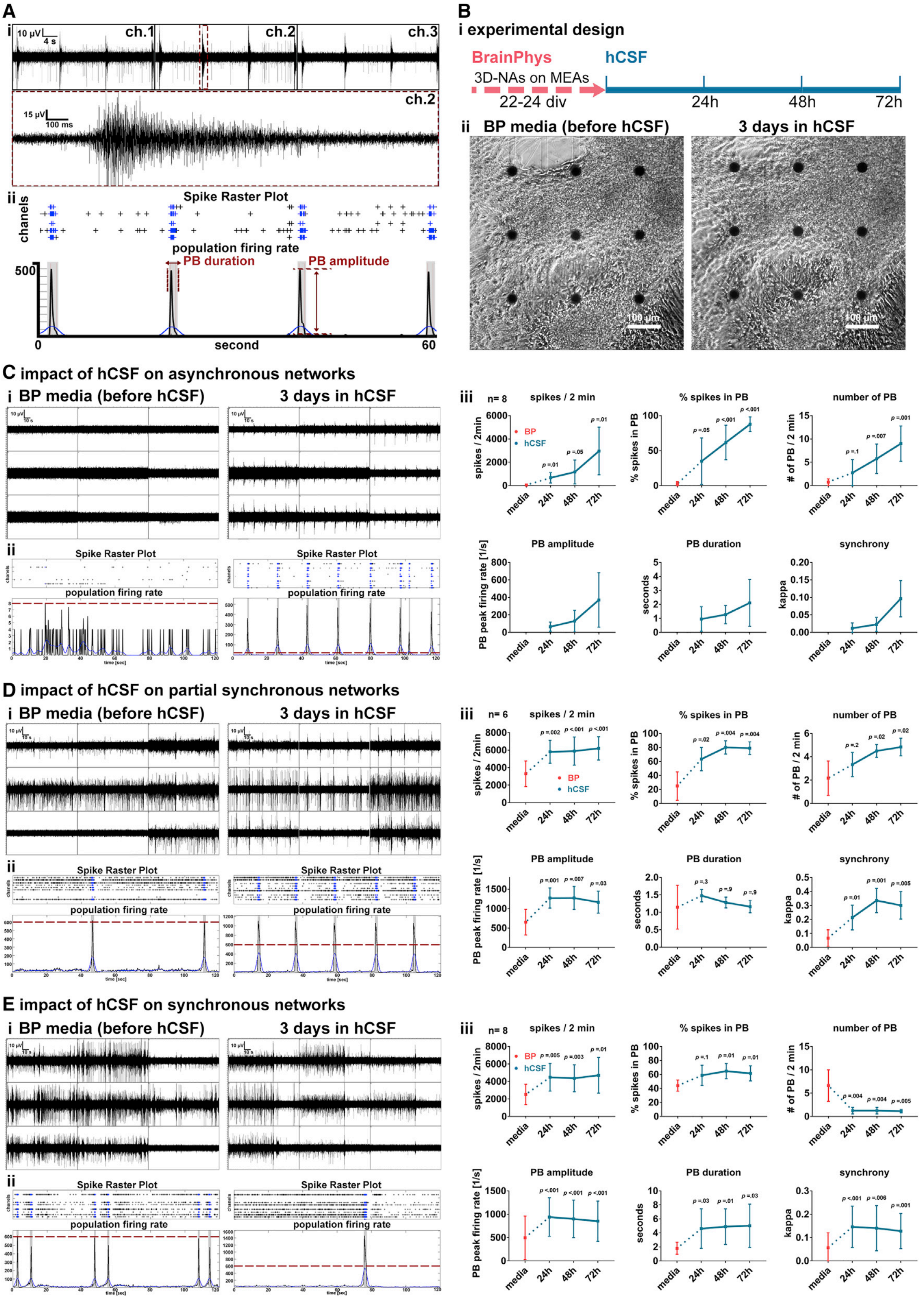

(legend on next page)

medium exchange with BP-based medium did not induce an increase of neuronal activity or changes in population burst properties (Figure S2A). However, hCSF treatment of those control cultures caused a progressive increase in neuronal network activity within 72 h (Figure S2A).

To exclude that the handling of hCSF samples, i.e., freezing and thawing of the samples, had an impact on the effect of hCSF on human neuronal network function described here, we tested freshly collected hCSF on 3D NA cultures on six-well MEAs. Forty minutes after CSF isolation, pre-warmed hCSF samples were applied on 3D NAs, which showed asynchronous, partially synchronous, or highly synchronous activity when cultured in BP-based culture medium. Identical to frozen samples (Figure 2), the fresh hCSF samples induced synchronous activity in all cultures and led to increased neuronal network activity within 72 h after hCSF application (Figure S2B).

Long-Term Cultures of hCSF-Treated hiPSC-3D NAs Maintain Enhanced Neuronal Network Activity

Next, we aimed to prove the feasibility of culturing 3D NAs in hCSF over a long time period and evaluated if hCSF-induced improved network activity further increased over time. For this purpose, we cultured hiPSC-derived networks for 4 weeks in hCSF and performed MEA recordings every day (n = 5, three hCSF samples were applied to one to two networks each). After hCSF treatment, 3D NA cultures showed robust attachment to MEA recording electrode array fields, and no detachment of 3D NAs was observed (Figure 3A). As described previously, hCSF caused a rapid increase of neuronal network activity within the first 3 days after hCSF application (Figure 3B). From day 3 to 11 after hCSF application, an increase in some neuronal network parameters could be observed; however, this was not significant. After 11 days, no neuronal parameters showed further changes, indicating a stable plateau phase (Figure 3B, ii) that lasted for the following 4-week recording period (Figure 3B, ii). Interestingly, population burst patterns of 3D NAs cultured for 14 and 28 days in hCSF were nearly identical (Figure 3B, i). By performing additional experiments using two additional hiPSC cell lines, we confirmed enhanced and stable neuronal network activity in 3D NA cultures under this long-term cultivation paradigm (Figure S3).

Short-Term Application of hCSF Causes Long-Lasting Improved Neuronal Network Activity in hiPSC-3D NAs

We assessed whether short-term hCSF treatment causes long-lasting changes in neuronal network function of hiPSC neurons. For this purpose, we applied BP-based medium to 3D NA cultures that had been cultured for 72 h in hCSF (Figure 4A, i). As described previously, initially asynchronously and partially synchronously active 3D NA cultures became highly synchronously active within 3 days in hCSF (n = 6, one hCSF sample was applied, Figures 4B and 4C). After 3 days in hCSF, hCSF was replaced by BP-based medium. We observed that the synchronous neuronal network activity remained (Figures 4B and 4C) and showed increased values for nearly all neuronal network parameters (Figures 4B, iv and 4C, iv) after switching back to BP-based medium. However, the level of synchrony and the percentage of spikes in population bursts had a decreasing tendency over time in BP-based medium. Nevertheless, these functional data demonstrate that short-term application of hCSF to 3D NAs causes long-lasting enhanced neuronal network activity of hiPSC neurons.

Complementary applied phase-contrast imaging showed that adherently growing 3D NAs cultured in BP-based cultivation medium move and grow over time (Figure S4A, see also Figure S2 in Izsak et al., 2019). In contrast, 3D NAs cultured in hCSF for 4 weeks neither showed signs of movement or overgrowth, nor detachment of 3D NAs adherently grown on MEAs (Figure S4B).

hCSF Induces Several Maturation Processes in hiPSC-3D NAs

We applied whole-cell voltage- and current-clamp recordings to assess the passive membrane, excitability and synaptic properties of neurons in 3D NAs that were either

Figure 2. Network Activity and Morphology of hiPSC-Derived Neurons after 72 h Exposure to hCSF

(A) (i) Representative example of MEA recording showing the appearance and properties of synchronous activity (three channels). The inset represents a detailed visualization of a population burst (PB) detected by one electrode. (ii) Example of spike raster plot and population firing rate illustrates synchronous network activity.

(B) (i) Schematic drawing shows the experimental design. (ii) Phase-contrast images show the morphology of cultures on a nine-electrode array of a six-well MEA, cultured in BP (left) and after 72 h cultivation in hCSF (right).

(C–E) Representative examples of (i) MEA recordings, (ii) spike raster plots and population firing rates illustrate the activity of asynchronously active (C), partial synchronously active (D), and synchronously active (E) neuronal populations cultured in BP before and after (72 h) exposure to hCSF. (C–E, iii) Diagrams illustrate the change of neuronal network parameters after the application of hCSF to initially asynchronously active (C, iii) (n = 8, N = 2), partial synchronously active (D, iii) (n = 6, N = 2), and synchronously active (E, iv) (n = 8, N = 2) neuronal populations. Data presented as average values ± SD. Matched one-way ANOVA with Dunnett correction (baseline compared with indicated group) and Tukey correction (comparison between groups) were applied to calculate indicated p values.

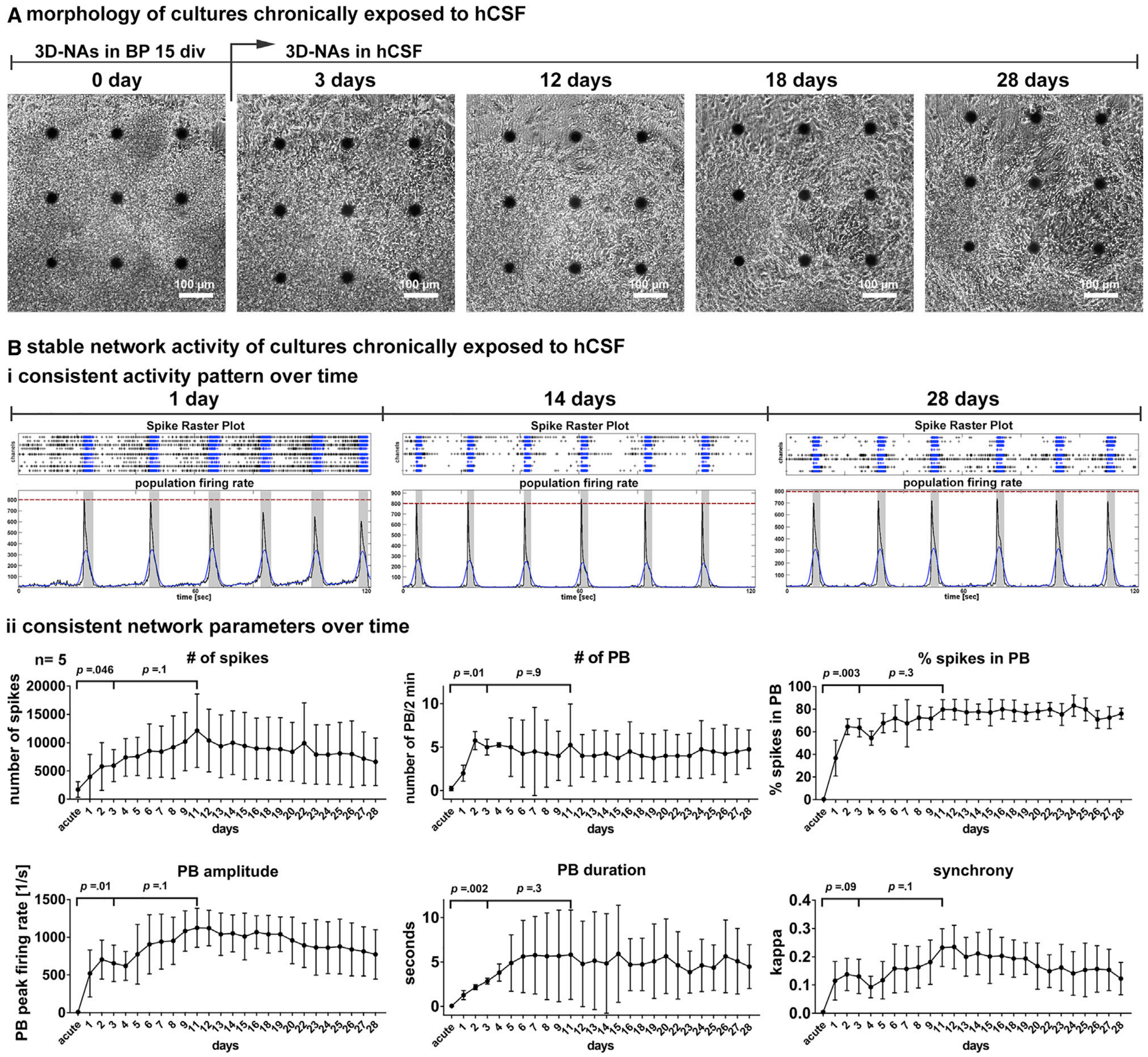

Figure 3. Morphology and Activity of hiPSC-Derived Neural Networks Cultured for 28 Days in hCSF

(A) Phase-contrast images show the morphology of cultures exposed to hCSF over time.

(B, i) Spike raster plots and population firing rates illustrate the activity of synchronous networks exposed to hCSF after 1, 14, and 28 days, respectively.

(B, ii) Diagrams illustrate network parameters after the application of hCSF over time (n = 5, N = 1). Data presented as average values ± SD. Matched one-way ANOVA with Dunnett correction (baseline compared with indicated group) and Tukey correction (comparison between groups) were applied to calculate indicated p values. PB, population burst

kept in BP-based medium or were cultured for 3 days in hCSF samples. The electrophysiological assessment for both groups was performed in artificial cerebrospinal fluid. Neurons at the edges of 3D NAs, in both BP-based medium and hCSF, were excitable and showed spontaneous excitatory ([sEPSCs], BP group: 17 out of 18, hCSF group: 18 out of 18) and inhibitory post-synaptic currents ([sIPSCs], BP group: 8 out of 18, hCSF group: 15 out of 18) (Figure 5B). Three days of hCSF treatment significantly increased the frequency of sEPSCs and sIPSCs (Figure 5C, i, ii) without altering the amplitude of sEPSCs and IPSCs (Figure 5C, iii, iv). A lower input resistance is commonly observed during neuronal maturation *in vitro* and *in vivo* (Ehrlich et al., 2012; Mongiat et al., 2009; Tadros et al., 2015; Takazawa

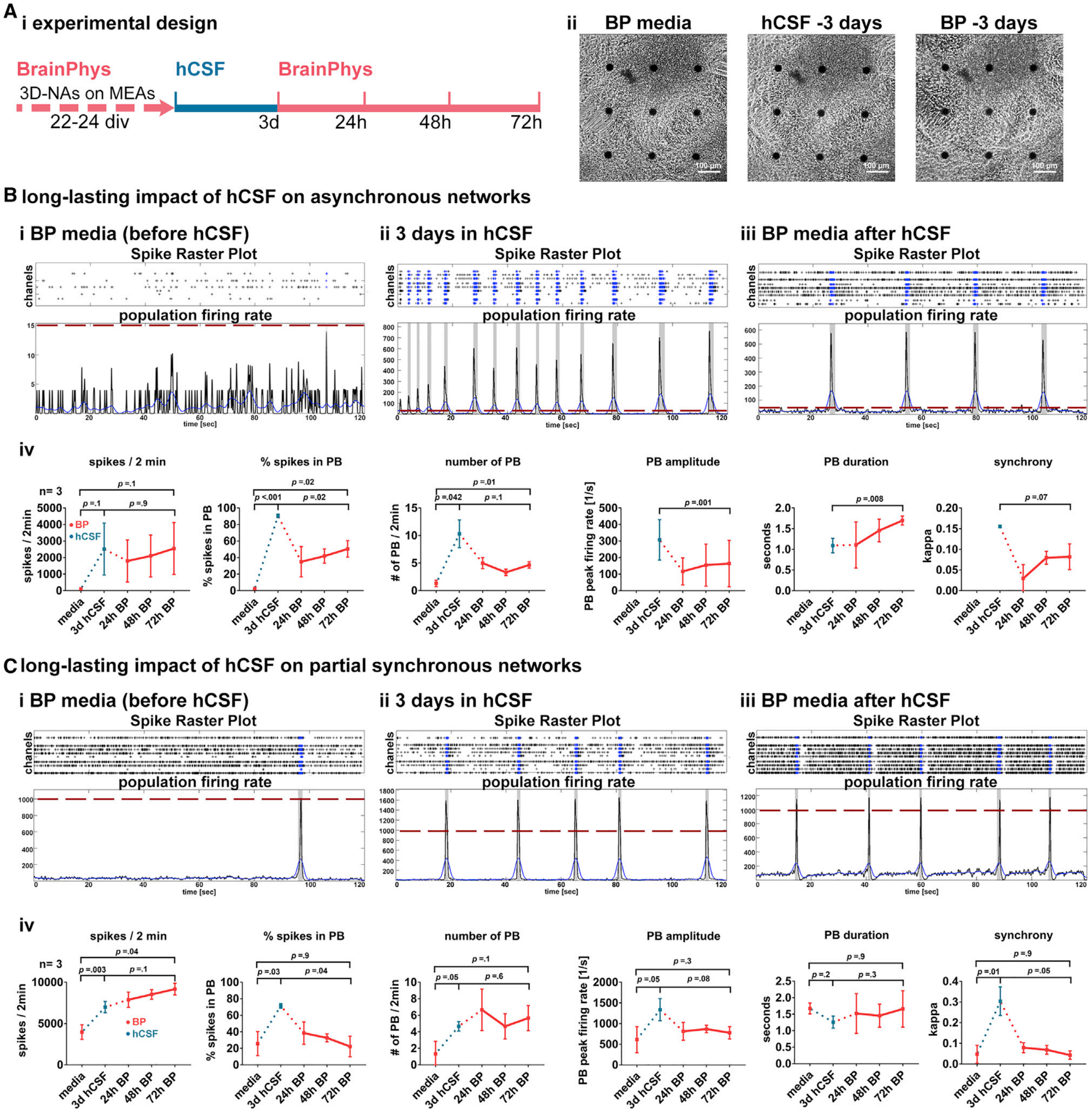

Figure 4. Long-Lasting Functional Alterations of hiPSC-Derived Neural Networks after Exposure to hCSF

(A) (i) Schematic drawing illustrates the experimental design. (ii) Phase-contrast images show the morphology of cultures on a six-well MEA, cultured in BP (left), exposed for 72 h to hCSF (middle), and re-exposed to BP for 72 h (right).

(B) Examples of spike raster plots and population firing rates illustrate the activity of asynchronous networks (cultured in BP) (i) before, (ii) after (72 h) exposure to hCSF, and (iii) after 72 h re-exposure to BP. (iv) Diagrams illustrate the network activity after the application of hCSF and the long-lasting impact after switching back to BP (n = 3, N = 1).

(C) Examples of spike raster plots and population firing rates illustrate the activity of partially synchronous networks (cultured in BP) (i) before, (ii) after (72 h) exposure to hCSF, and (iii) after 72 h re-exposure to BP. (iv) Diagrams illustrate network activity after the application of hCSF and the long-lasting impact after switching back to BP (n = 3, N = 1). Data presented as average values ± SD. Matched one-way ANOVA with Dunnett correction (baseline compared with indicated group) and Tukey correction (comparison between groups) were applied to calculate indicated p values. PB, population burst.

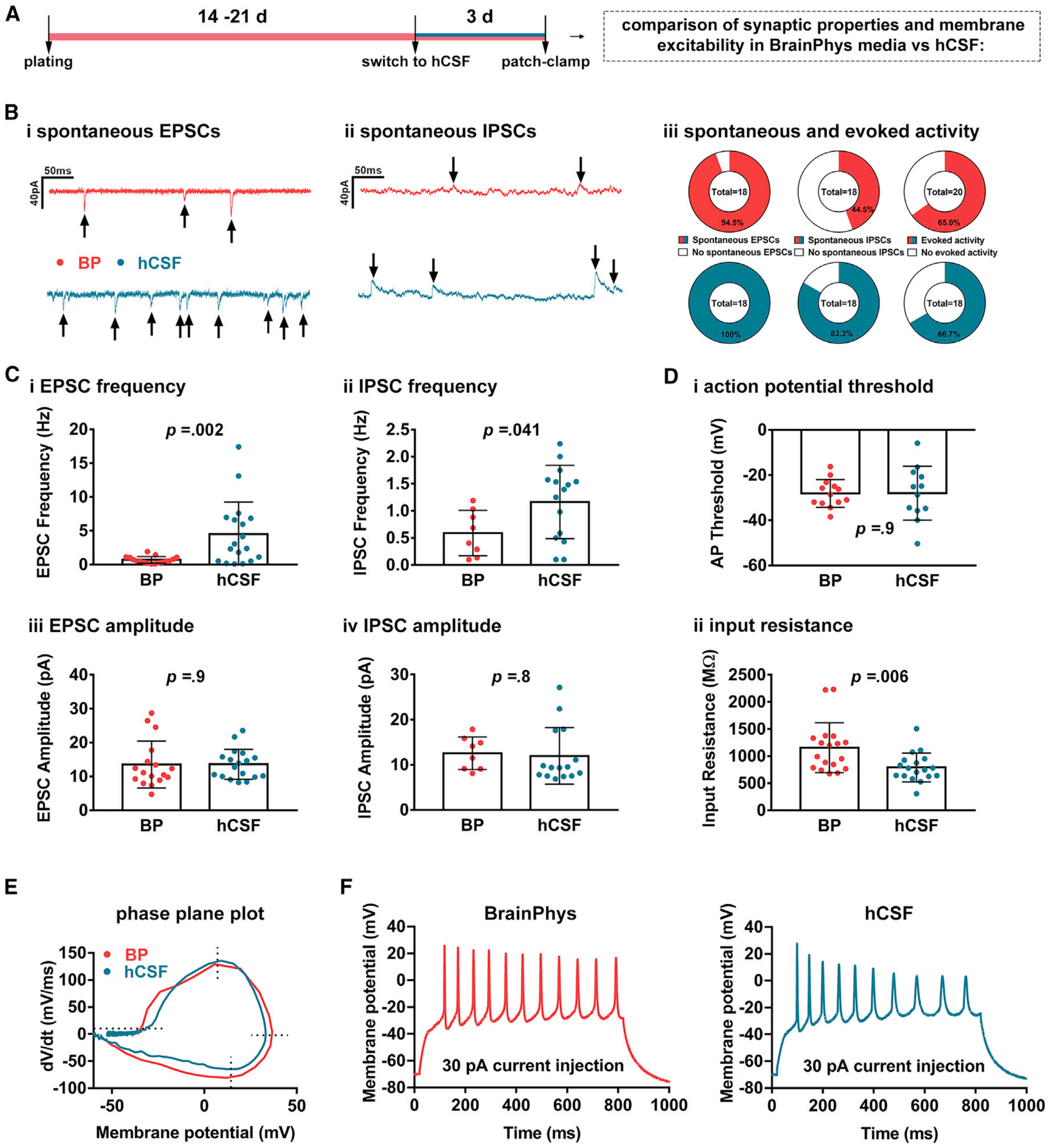

Figure 5. hCSF Triggers Maturation of Synaptic Properties and Connectivity of Individual hiPSC Neurons

(A) Schematic drawing illustrating the experimental design.

(B) Examples of whole-cell patch-clamp recordings of (i) spontaneous excitatory post-synaptic currents (sEPSCs) and whole-cell patch-clamp recordings of (ii) spontaneous inhibitory post-synaptic currents (sIPSCs) recorded from neurons at the edges of 3D NAs (BP, red or hCSF, blue). Arrows denote active events. Pie charts show the percentage of cells (iii) with sEPSCs, sIPSCs, and evoked activity (in current clamp). Total number of cells is given in the center.

(C) Average frequency (i and ii) and amplitude (iii and iv) of sEPSCs and sIPSCs recorded from cells cultured in either BP or hCSF, with individual recordings shown as scattered dots.

(D) Average action potential (AP) threshold (i) and input resistance (ii). AP threshold was determined from a phase plane plot at a dV/dt of 10 mV/ms.

(legend continued on next page)

et al., 2012), and it is likely a consequence of a larger and more complex dendritic arbor, which should increase the connectivity. Since the hCSF-treated neurons appear larger, have a lower input resistance (Figure 5D, ii), and show the described substantial increase in spontaneous synaptic activity, we conclude that hCSF-induced neuronal maturation occurs on a single neuronal level.

When injecting currents at stepwise increments with the cell in current clamp, 13 out of 20 and 12 out of 18 neurons showed evoked responses, i.e., action potentials (APs), for BP and hCSF, respectively (Figure 5F; B, iii). From these APs we constructed phase plane plots to calculate the AP threshold (Figure 5E; D, i), the maximal rate of depolarization, amplitude and maximal rate of repolarization. There were no significant differences when comparing AP threshold (−28.12 ± 6.15 versus −28.02 ± 11.96 mV) AP amplitude (62.0 ± 16.1 versus 69.6 ± 14.1 mV), AP half-width (1.7 ± 0.5 versus 2.1 ± 0.7 ms), rheobase (49.3 ± 17.5 versus 43.3 ± 11.5 pA), and maximal rate of depolarization (119.3 ± 91.4 versus 117.9 ± 47.7 mV/ms), repolarization (−51.5 ± 31.8 versus −52.7 ± 20.2 mV/ms), for neurons grown in BP and hCSF, respectively.

Next, we applied immunofluorescent staining and fluorescent confocal imaging to obtain a more detailed morphological and cellular insight into the properties of neural cells in 3D NAs cultured in hCSF or BP-based medium.

Assessment of the βIII-tubulin$^+$ neurite net around 3D NAs cultured for 3 days in hCSF, or in BP-based medium (Figure 6B, ii; Video S1), revealed a much more intense neurite net around individual 3D NAs cultured in hCSF (Figure 6B). Note that the imaging settings (e.g., detector gain, laser intensity, etc.) were the same for both groups. Since we observed a significant increase of the number of MAP-2AB$^+$-neurons in the hCSF-treated group (Figure 6C; Video S2), we assessed the number of electrodes that detected neuronal activity. Asynchronously and partially synchronously active 3D NA culture showed a progressive increase of active electrodes, and after 3 days in hCSF nearly all cultures showed electrodes detecting neuronal activity (Figure 6D), which persisted even after removal of hCSF and application of BP-based cultivation medium (Figure 6D, ii, v). However, highly synchronously active cultures did not show such changes (data not shown).

Next, we assessed the number of PSD-95$^+$ post-synapses and VGlut1$^+$ pre-synaptic structures within 3D NAs (Figure 6E). Co-immunocytochemistry and confocal imaging showed that the post-synaptic protein PSD-95 was associated with pre-synaptic protein synapsin indicative of mature synapses (Figure S5). We observed that MAP2AB$^+$ neurons cultured in BP-based medium showed rather a cytoplasmic and less a dot-like synaptic VGlut1 sub-cellular localization (Figure 6E, iii, left). In contrast, MAP2AB$^+$ neurons kept for 3 days in hCSF showed rather a dot-like synaptic and less a cytoplasmic VGlut1 sub-cellular localization (Figure 6E, iii, right). Note that cytoplasmic localization of VGlut proteins occurs in immature neurons and that the translocation of VGlut proteins into the synapse is associated with neuronal maturation and synapse formation (Illes et al., 2009; Real et al., 2006). The image area was chosen in relation to the individual size of the 3D NAs to achieve a reliable quantitative assessment of the PSD-95 and VGlut1 signals in different sized 3D NAs (for details, see Supplemental Experimental Procedures). By this approach, we revealed a significant increase of PSD-95$^+$ post-synapses and VGlut1$^+$ pre-synapses caused by 3 days hCSF treatment of 3D NAs (Figure 6E, ii, iv).

These electrophysiological and imaging data demonstrate that hCSF induces a rapid increase of the number of electrophysiological active neurons, triggers synapse development, neuronal maturation, and the formation of a dense neurite net.

Next, we performed GFAP stainings to visualize prospective astroglia cells within and around 3D NAs (Figure 7C). After 3 days of treatment with hCSF, 3D NAs showed numerous GFAP$^+$ cells outside (Figure 7C, ii) and inside of 3D NAs (Figure 7C, iii), which were very rarely present in 3D NAs cultured in BP-based culture medium (Figure 7C). hCSF-induced GFAP cells showed elongated shape and had processes spanning throughout an entire 3D NA (Figure 7C). An increase of the detector gain was required to visualize the few GFAP cells in BP-based medium cultured 3D NA cultures, indicating that GFAP cells in BP-based medium have lower expression of GFAP than hCSF-induced GFAP cells (Figure S6). Quantitative assessment of the percentage of area covered by GFAP$^+$ cells at the edges of adherently growing 3D NAs showed significantly higher values in the hCSF-treated group (Figure 7C, iv).

We stained for additional astrocyte-specific markers and confirmed that 3D NA cultures contained S100β, aquaporin-4$^+$, and glutamine synthase$^+$ astrocytes, which either co-expressed GFAP or were GFAP$^−$ (Figure 7B). We observed that hCSF-cultured 3D NAs showed numerous S100β$^+$ astrocytes outside (Figure 7C, i, ii) and inside of 3D NAs,

(E) Examples of phase plane plots. Dotted lines denote AP threshold (at 10 mV/ms), maximal rate of depolarization, amplitude, and maximal rate of repolarization, respectively.
(F) Examples of evoked firing responses to an 800-ms current injection of 30 pA into a current-clamped neuron cultured in BP or hCSF. Cells cultured in BP and hCSF were recorded from pairs per experiment. Error bars denote SD and p values were calculated by an unpaired, Student's t test. Data were obtained from two independent experiments.

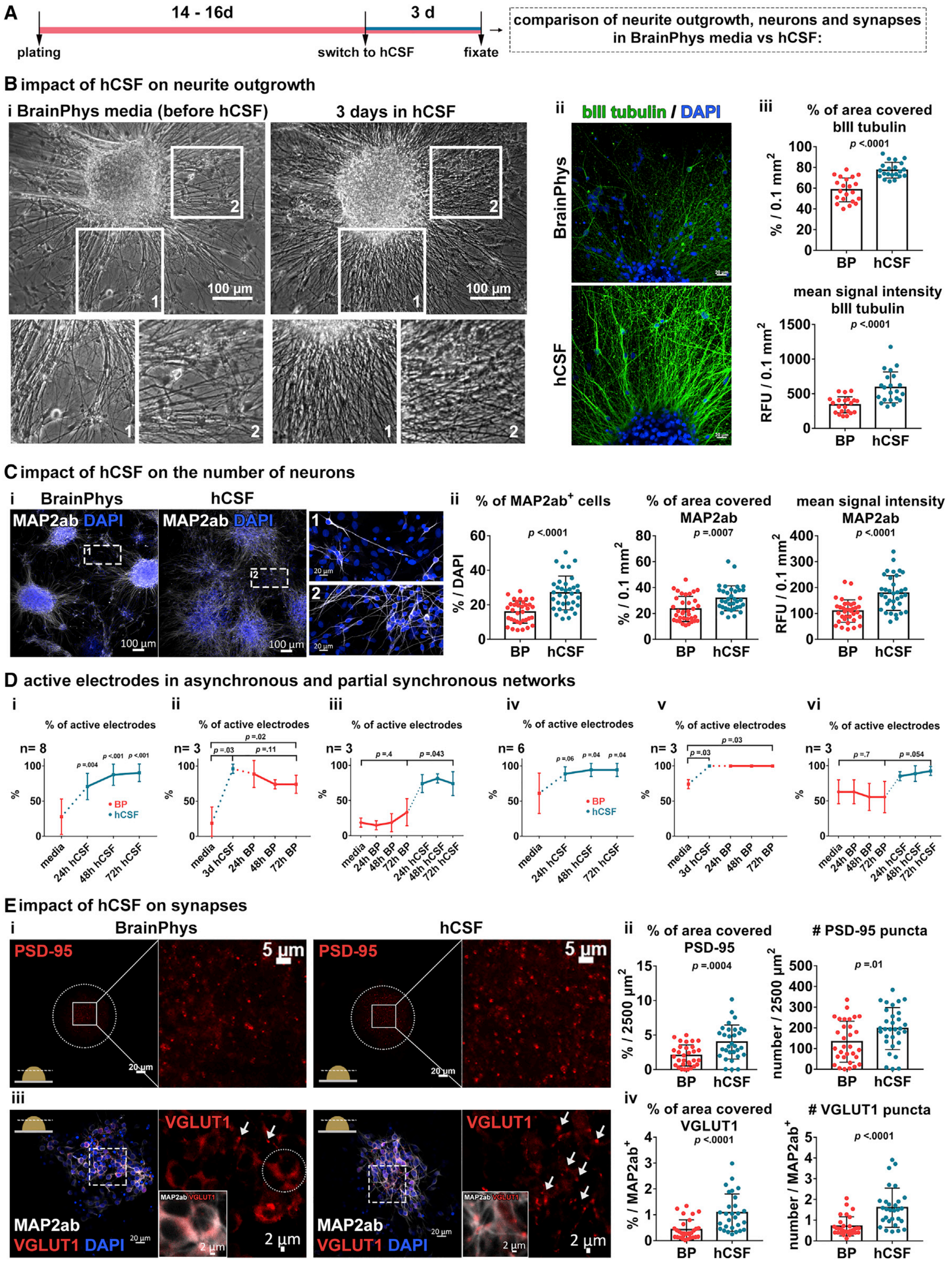

Figure 6. hCSF Triggers Rapid Neuronal Differentiation and Synapse Development

(A) Schematic drawing illustrating the experimental design.

(B) (i) Phase-contrast images show the morphology of cultures in BP (left) and after exposure for 72 h to hCSF (right), the insets illustrate the marked regions of interest (ROIs) in higher magnification. (ii) Confocal images visualize βIII-tubulin+ neurites from 3D NAs cultured in

(legend continued on next page)

which were less present in 3D NAs cultured in BP-based culture medium (Figure 7C, iii; Videos S3 and S4). Since quantification confirmed that hCSF substantially increased the number of S100β$^+$ astrocytes (Figure 7C, v), the data demonstrate that hCSF causes rapid astrocyte development in 3D NAs cultures.

Since phase-contrast imaging revealed that 3D NAs cultured in hCSF did not show signs of neural overgrowth of 3D NAs (Figure S4), we applied different markers for proliferating neural stem/progenitor cells. Confocal imaging of DAPI-stained nuclei allows to assess the height of 3D NAs. We observed that hCSF-treated 3D NAs had a significantly lower height than BP medium-treated 3D NAs (Figure 7D, iv; Video S5). In addition, we observed significantly more condensed DNA (DAPI nuclei) in the 3D NAs cultured in BP medium in comparison with hCSF-treated cultures (Figure 7D, i and iv, see also DAPI cells in Videos S1, S2, S3, and S4). By applying the proliferation marker Ki-67, we confirmed that condensed DNA (DAPI nuclei) was present in Ki-67 proliferating cells (Figure 7D, ii). In addition, we observed nuclei with condensed DNA in Nestin$^+$ NSCs and Ki-67$^+$/SOX-2$^+$ NSCs (Figure 7D, ii, iii). In contrast, caspase-3$^+$ apoptotic cells have rather a disrupted DAPI-stained DNA (Figure 7D, ii) and we did not observe apoptotic cells with condensed nuclei. We counted the number of Ki-67$^+$ proliferating cells and confirmed that a 3-day hCSF treatment of 3D NA cultures resulted in significant reduction of proliferating cells (Figure 7D, iv). Interestingly, the number of SOX-2 NSCs in hCSF-treated cultures was slightly, but not significantly reduced (Figure 7D, iv).

DISCUSSION

In an hiPSC-derived 3D NA *in vitro* model (referred to as 3D NA), we applied hCSF from healthy adult individuals to evaluate, if a physiologically relevant and adult brain-like milieu triggers neural maturation processes and enhances neuronal functionality in 3D NAs with immature properties. We demonstrated that hCSF induces several maturation processes, including neurogenesis, electrophysiological maturation of neurons, gliogenesis, and synapse and neurite net formation, which ultimately leads to a rapid formation of synchronously active neuronal circuits in 3D NA cultures. Since patch-clamp and MEA recordings demonstrate the improved electrophysiological function of neurons and enhanced neuronal circuit activity, which persists after removal of hCSF, we conclude that hCSF-induced maturation processes are responsible for this enhanced functionality on single neuronal and neuronal circuit level.

Implications for hiPSC-Based Neural Development and Neuronal Circuits in *In Vitro* Models

Astrocytes promote synapse formation, neurite growth, and neuronal electrophysiological function (Halassa et al., 2007; Johnson et al., 2007; Tang et al., 2013). Here, we present that the increased number of neurons, a denser neurite net, increased number of PSD-95 and VGlut1 synapses, as well as enhanced individual neuronal electrophysiological and neuronal circuit function correlates with an increased number of S100β astrocytes. Thus, we surmise that, yet unknown, hCSF molecules and hCSF-induced astrocytes caused the enhanced synapse development and supported neurite growth resulting in a human neuronal population with increased functional connectivity and synchronous neuronal activity.

Several studies have shown that the differentiation of hiPSCs into human astrocytes requires 3 to 6 months (e.g., Tcw et al., 2017) and early cultures of hiPSC-derived NSCs have a rather neurogenic than a gliogenic differentiation capacity (e.g., Edri et al., 2015; Gaspard et al., 2008). Tchieu et al. (2019) recently demonstrated that within 5 days NFIA overexpression induced a gliogenic fate in neurogenic

BP (above) and hCSF (below). (iii) Diagrams present quantified parameters for βIII-tubulin$^+$ neurite net in the cultures treated with hCSF for 3 days (5 images/coverslip, n = 3, N = 3).
(C) (i) Confocal images visualize MAP2AB$^+$ neurons in 3D NA cultures under BP (left) and hCSF (right). Detailed images of MAP2AB$^+$ neurons outside the 3D NAs. (ii) Diagrams present quantified parameters for MAP2ab$^+$ neurons (5 images/coverslip, n = 2–4, N = 3).
(D) Diagrams show the percentage of recording MEA electrodes detecting neuronal activity in asynchronous (i, ii, iii) and partial synchronous networks (iv, v, vi) (n indicates number of wells from N = 2).
(E) (i) Confocal images visualize the PSD-95^{+-} synapses in 3D NA cultures with BP (left) or hCSF (right) culture conditions. Circles mark the position of 3D NAs and the boxes represent the ROIs shown in a higher magnification. (ii) Diagrams show the percentage of image area that shows PSD-95 signal and the number of PSD-95 objects (5 images/coverslip, n = 3, N = 3). (iii) Confocal images visualize the VGlut1$^+$ synapses along the MAP2AB$^+$ neurons in 3D NA cultured with BP (left) or hCSF (right). The dashed box marks the ROI presented in higher magnification on the right. The arrows mark synaptic puncta and the circle shows the cytoplasmatic localization of VGlut1 signal in MAP2AB$^+$ neurons cultured in BP. The inlet images show the co-localization of VGlut1 signal with the MAP2AB. (iv) Diagrams present quantified parameters for VGlut1$^+$ synaptic puncta per MAP2AB$^+$ neuron (5 ROIs/coverslip, n = 3, N = 2). Data presented as average values ± SD. Two-tailed, unpaired t test were applied to calculate indicated p values. For MEA diagrams (D) matched one-way ANOVA with Dunnett correction (baseline compared with indicated group) and Tukey correction (comparison between groups) were applied to calculate indicated p values.

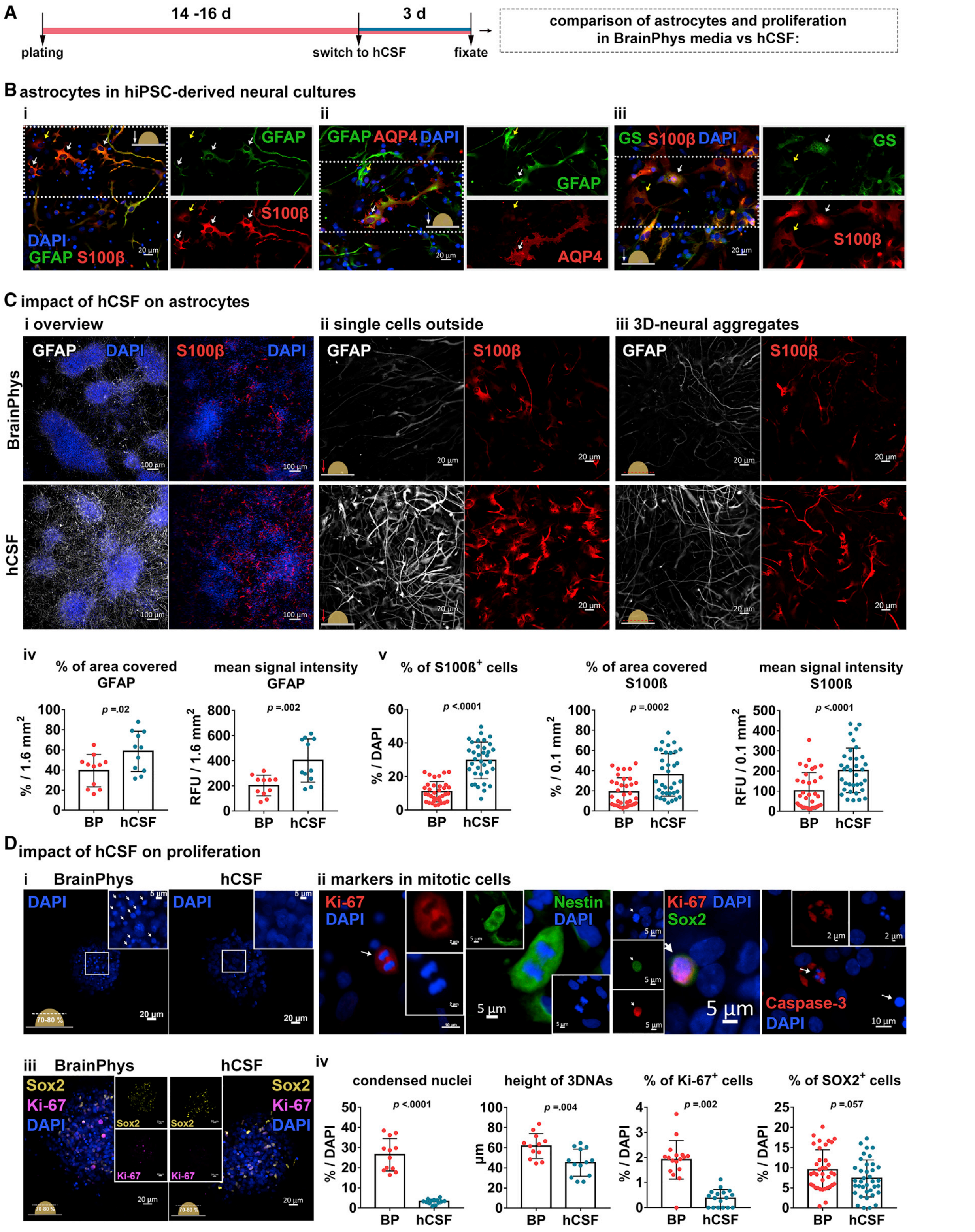

(legend on next page)

hiPSC-derived NSCs. The differentiation of those glia-committed human NSCs required silencing of NFIA and, in addition, 14 days to significantly detect the development of $GFAP^+$ astrocytes (Tchieu et al., 2019). In contrast, here we demonstrate that hCSF induced the development of GFAP/S100β astrocytes within 3 days in 3D NAs (56–60 days post iPSC stage). This unexpected and very intriguing observation demonstrates that *in vitro* hiPSC neural cells have the capacity to rapidly adopt an astrocytic phenotype (3 days) *in vitro* when exposed to an appropriate *in vitro* milieu. Since astrocytes represent a heterogeneous group that differs in its capacity to enhance the neuronal properties and astrocyte development requires a diverse set of signaling cues, future studies will help to understand the mechanism of hCSF-induced astrocyte development in human NSCs and to identify novel human gliogenic signaling cues.

As a control condition, we used current, state-of-the art culture medium, the BP medium supplemented with commonly used differentiation factors, e.g., BDNF, GDNF, DAPT. However, these culture conditions are not sufficient to achieve morphological and functional properties in a human 3D neural model as described here by using hCSF. From a neurodevelopmental aspect it is interesting that hCSF not only causes astrocyte development, but induces neuronal differentiation, neuronal maturation, and suppresses proliferation. Thus, adult hCSF contains currently unknown factors that might be necessary to promote neurogenesis and suppression of proliferation to achieve terminal neural maturation in other 3D human neural *in vitro* models, e.g., brain organoids.

In previous studies, we and others showed that hCSF is superior to artificial cerebrospinal fluid and neurobasal-based cultivation medium in promoting electrophysiological function of *in vitro* neurons in primary neuronal cultures (Perez-Alcazar et al., 2016), murine embryonic stem cell cultures (Otto et al., 2009), rodent brain slices (Bjorefeldt et al., 2015, 2016), and human brain slices (Schwarz et al., 2017; Wickham et al., 2020). However, the morphological and functional supportive impact of hCSF in hiPSC-derived 3D neural *in vitro* models was unknown. A limitation of all previous work, including ours, is the application of hCSF obtained from unhealthy individuals. For instance, pooled CSF samples obtained from hydrocephalus patients were used as "control" CSF in all previous works (Buddensiek et al., 2009, 2010; Gortz et al., 2013; Jantzen et al., 2013; Koch et al., 2019; Otto et al., 2009; Schwarz et al., 2017; Wickham et al., 2020). It has been known for decades that the chemical composition of hydrocephalus CSF differs from healthy CSF (e.g., Del Bigio, 1989), which indicates that these CSF samples might not be suitable to create a healthy, adult brain-like milieu *in vitro*. Thus, a comparison of the impact of healthy and hydrocephalus CSF on hiPSC neural *in vitro* models represents an interesting approach for a future study. Furthermore, so far only frozen hCSF samples have been used for *in vitro* experiments, and it was unknown if the freezing and storage procedure influenced the quality of hCSF in *in vitro* experiments. Here, we demonstrated that the impact on human neuronal circuit formation of fresh, unfrozen hCSF and frozen hCSF is identical. Thus, our present work shows the impact of non-pathological and fresh hCSF on human *in vitro* neuronal circuit development and function.

A common hallmark of immaturity in hiPSC-based *in vitro* neural model systems, including brain organoid cultures (Lancaster et al., 2013), is ongoing proliferation of residing NSCs, leading to 3D neural cell assemblies that grow for several months *in vitro* (Qian et al., 2019). Within the development of the fetal into the adult brain, CSF-derived factors regulate proliferation, quiescence, and differentiation of NSCs, as described by a plethora of literature (for review see Gato et al., 2005; Kalamakis et al., 2019; Obernier and Alvarez-Buylla, 2019; Zappaterra and Lehtinen, 2012). Here, we demonstrate that hiPSC-derived neural cultures exposed to an adult brain-like milieu (here: adult hCSF) show suppressed proliferation in 3D NA cultures within 3 days. Interestingly, the number of SOX-2

Figure 7. hCSF Triggers Rapid Astrocyte Development and Reduction of Proliferation
(A) Schematic drawing of the experimental design.
(B) Confocal images visualize astrocytic markers in hiPSC-derived neural cultures: (i) $S100\beta^+$ astrocytes that co-express GFAP (white arrows) or only S100β (yellow arrow), (ii) $GFAP^+$ astrocytes that co-express aquaporin (white arrow) or only GFAP (yellow arrow), (iii) $S100\beta^+$ astrocytes that co-express glutamine synthetase (white arrow) or only S100β (yellow arrow).
(C) (i) Confocal images visualize $GFAP^+$ and $S100\beta^+$ astrocytes in 3D NA cultures under BP (above) and hCSF (below). (ii) Detailed images of $GFAP^+$ and $S100\beta^+$ astrocytes outside and (iii) inside of aggregates. Note, images for GFAP and S100β were taken with identical image settings for both groups. (iv) Diagrams present quantified parameters of $GFAP^+$ cells (1 image/culture, n = 2–3, N = 3). (v) Diagrams present quantified parameters of $S100\beta^+$ astrocytes (5 images/coverslip, n = 2–3, N = 3).
(D) Confocal images visualize the $DAPI^+$ nuclei in 3D NA cultured with BP (left) or hCSF (right). Boxes mark the ROIs shown in higher magnification, arrows indicate the presence of condensed nuclei in BP. (ii) Confocal images show the presence of DAPI-condensed DNA in nuclei of Ki-67^+, $Nestin^+$, $Sox2^+$ cells. Note, caspase-3^+ apoptotic cells have fragmented $DAPI^+$ DNA and casapase-3 is absent in DAPI-condensed DNA (arrow). (iii) Confocal images visualize Ki-67^+/$Sox2^+$ and Ki-67^-/$Sox2^+$ NSCs in 3D NAs cultured with BP (left) or hCSF (right). (iv) Diagrams present quantified parameters of $DAPI^+$-condensed nuclei, height of 3D NAs, Ki-67^+ proliferating cells, and $Sox2^+$ NSCs. Data are presented as mean ± SD. Two-tailed, unpaired t tests were applied to calculate indicated p values.

NSCs is not significantly reduced by a 3-day hCSF treatment. Within the same time period, hCSF treatment causes a ~1.5-fold increase in the number of MAP2AB$^+$ neurons and an ~3-fold increase in the number of S100β astrocytes. Furthermore, overgrowth of 3D NAs is absent after hCSF application, and the reduction of mitotic nuclei, e.g., in Nestin$^+$ cells, by hCSF is evident. Thus, we conclude that the absence of neural overgrowth in hCSF-treated cultures might not be predominately mediated by differentiation of SOX-2 NSCs, which would have led to a significant reduction of the SOX-2 NSC population. We rather assume that hCSF induces the differentiation of proliferative neuronal and glial progenitor cells into neurons and astrocytes, respectively. Since SOX-2 is present in proliferative and non-proliferating quiescent NSCs (Lugert et al., 2010; Surzenko et al., 2013), we assume that some non-proliferating quiescent SOX-2$^+$ NSCs persist even in the presence of very potent differentiation cues as present in hCSF.

Of course, the question about factors within hCSF, which rapidly promote functional human *in vitro* neuronal circuit development, astroglial and neuronal development, neurite growth, synapse formation, and suppression of proliferation, is highly interesting—however, also challenging to answer. It is extremely unlikely that only one single factor is responsible to trigger the different maturational processes described here. Moreover, we can envision that certain factors might promote the transition from NSCs into neurons, while other factors are required to promote astrocyte development, neurite growth, and synapse development. Since we observed hCSF-enhanced glia development, it is reasonable to expect that these glial cells secrete additional factors, which then further promote neurite growth and synapse development of hiPSC-derived neurons. To highlight the complexity of specific factor identification even more, fetal bovine serum (FBS) is commonly used to create a gliogenic milieu *in vitro* (e.g., Tchieu et al., 2019), and thus used to induce astrocyte development of human NSCs. However, FBS strongly suppresses neuronal network activity (Figure S7) (see also Bardy et al., 2015). Thus, our present work represents the rationale for future studies, which aim to identify novel physiological factors for the improvement of neural development and neuronal function in hiPSC neural *in vitro* models.

EXPERIMENTAL PROCEDURES

Generation of Human iPSC-3D NAs

hiPSC lines (C1, C2, C3) were cultured and differentiated into cortical NSCs as described elsewhere (Hayashi et al., 2015; Vizlin-Hodzic et al., 2017). Within 10–14 days, hiPSC NSCs formed 3D NAs (Edri et al., 2015; Izsak et al., 2019) and 3D NAs were manually transferred on coverslips or MEAs and cells were kept in BP medium with supplements.

Multi-electrode Array Recordings

Two to five hiPSC-3D NAs were seeded as a 5-μL drop directly on PDL/laminin-coated electrode arrays of six-well PEDOT-CNT MEAs. After 1 h, BP medium with supplements was added. Half medium exchanges were performed twice a week. Baseline recordings have been performed in BP medium with supplements before the application of either hCSF or fresh culture medium. Data were recorded and analyzed using MC_Rack software (Multi Channel Systems), SPANNER software suite (RESULT Medical), and custom-built MATLAB software (Hedrich et al., 2014; Izsak et al., 2019).

Whole-Cell Patch-Clamp Recordings and Data Analysis

Five to ten hiPSC-3D NAs were seeded on PDL/laminin-coated coverslips and cultured with BP medium with supplements. Half medium exchanges were performed twice a week, 14–20 days after differentiation. For the experiment, coverslips were mounted under a differential interference microscope (Nikon E600FN) together with a CCD camera (Sony XC-73CE) to visually identify the cells. Cells were perfused (2–3 mL/min) with artificial CSF. The micropipette was filled with an intracellular solution. The data were collected with a sampling frequency of 10 kHz and filtered at 3 kHz using an EPC-9 amplifier (HEKA Elektronik, D-67466 Lambrecht/Pfalz, Germany). Whole-cell recordings were carried out at 32°C and all recordings were performed between the second (14 days) and third weeks (21 days) *in vitro*. Calculations and data analysis were performed in custom-made IGOR Pro 8 (WaveMetrics, Lake Oswego, OR, USA) software.

For further details and details about hCSF sample collection, immunocytochemistry, image acquisition, and analyses, as well as statistical analysis see Supplemental Experimental Procedures.

Statistical Analysis

For statistical analysis either matched one-way ANOVA with Dunnett correction (baseline compared with indicated group) or Tukey correction (comparison between groups) were applied; two-way, unpaired t test was applied to calculate indicated p values. All presented data show mean value ± SD, n refers to the number of individual cultures treated with hCSF or BP medium and N refers to the number of individual experiments. For statistical analysis, GraphPad Prism 8.0 software was used.

SUPPLEMENTAL INFORMATION

Supplemental Information can be found online at https://doi.org/10.1016/j.stemcr.2020.05.006.

AUTHORS CONTRIBUTIONS

J.I. performed the experiments, analyzed the data, and prepared the figures. H.S. performed patch-clamp experiments. S.T. developed data analysis programs and critically revised the manuscript. E.H. critically revised the manuscript. S.I. conceived the study, performed part of the experiments and wrote the manuscript.

CONFLICTS OF INTEREST

S.I. holds a position at Cellectricon. Cellectricon were not involved in the study, and all experiments and data analyses

were conducted at the Sahlgrenska Academy at the University of Gothenburg.

S.T. is founder of Result Medical GmbH, Düsseldorf, Germany. The other authors declare no conflict of interest.

ACKNOWLEDGMENTS

The authors thank Pontus Wasling and Henrik Zetterberg for providing human CSF samples, Keiko Funa and Hans Ågren for providing human iPSC lines, Marcela Pekna and Ulrika Wilhelmsson for providing astrocyte antibodies, and the Patrik Rorsman group for providing access to the LSM720 microscope. S.T. received support from the German Ministry of Education and Research (BMBF: FKZ 031B0010B) and the European Union (EuroTransBio9 project In-HEALTH). This work was supported by research grants issued by the Alzheimerfonden, Sweden (AF-556051/AF-744871 to S.I. and AF-640391 to E.H.), the Frederik and Ingrid Thuring Foundation, Sweden (2016-0225 to S.I.), Magnus Bergvalls Stiftelse, Sweden (to S.I.), Swedish Research Council 2016-00986 (to E.H.), Stiftelsen Psykiatriska Forskningsfonden, Sweden (to S.I.), Swedish State Support for Clinical Research ALFGBG-427611 (to E.H.), and the Åke Wisberg Foundation, Sweden (M17-0265, to S.I.).

Received: November 1, 2019
Revised: May 6, 2020
Accepted: May 10, 2020
Published: June 9, 2020

REFERENCES

Bachy, I., Kozyraki, R., and Wassef, M. (2008). The particles of the embryonic cerebrospinal fluid: how could they influence brain development? Brain Res. Bull. *75*, 289–294.

Bardy, C., van den Hurk, M., Eames, T., Marchand, C., Hernandez, R.V., Kellogg, M., Gorris, M., Galet, B., Palomares, V., Brown, J., et al. (2015). Neuronal medium that supports basic synaptic functions and activity of human neurons in vitro. Proc. Natl. Acad. Sci. U S A *112*, E2725–E2734.

Del Bigio, M.R. (1989). Hydrocephalus-induced changes in the composition of cerebrospinal fluid. Neurosurgery *25*, 416–423.

Bjorefeldt, A., Andreasson, U., Daborg, J., Riebe, I., Wasling, P., Zetterberg, H., and Hanse, E. (2015). Human cerebrospinal fluid increases the excitability of pyramidal neurons in the in vitro brain slice. J. Physiol. *593*, 231–243.

Bjorefeldt, A., Wasling, P., Zetterberg, H., and Hanse, E. (2016). Neuromodulation of fast-spiking and non-fast-spiking hippocampal CA1 interneurons by human cerebrospinal fluid. J. Physiol. *594*, 937–952.

Borghese, L., Dolezalova, D., Opitz, T., Haupt, S., Leinhaas, A., Steinfarz, B., Koch, P., Edenhofer, F., Hampl, A., and Brustle, O. (2010). Inhibition of notch signaling in human embryonic stem cell-derived neural stem cells delays G1/S phase transition and accelerates neuronal differentiation in vitro and in vivo. Stem Cells *28*, 955–964.

Buddensiek, J., Dressel, A., Kowalski, M., Storch, A., and Sabolek, M. (2009). Adult cerebrospinal fluid inhibits neurogenesis but facilitates gliogenesis from fetal rat neural stem cells. J. Neurosci. Res. *87*, 3054–3066.

Buddensiek, J., Dressel, A., Kowalski, M., Runge, U., Schroeder, H., Hermann, A., Kirsch, M., Storch, A., and Sabolek, M. (2010). Cerebrospinal fluid promotes survival and astroglial differentiation of adult human neural progenitor cells but inhibits proliferation and neuronal differentiation. BMC Neurosci. *11*, 48.

Edri, R., Yaffe, Y., Ziller, M.J., Mutukula, N., Volkman, R., David, E., Jacob-Hirsch, J., Malcov, H., Levy, C., Rechavi, G., et al. (2015). Analysing human neural stem cell ontogeny by consecutive isolation of Notch active neural progenitors. Nat. Commun. *6*, 6500.

Ehrlich, D.E., Ryan, S.J., and Rainnie, D.G. (2012). Postnatal development of electrophysiological properties of principal neurons in the rat basolateral amygdala. J. Physiol. *590*, 4819–4838.

Gaspard, N., Bouschet, T., Hourez, R., Dimidschstein, J., Naeije, G., van den Ameele, J., Espuny-Camacho, I., Herpoel, A., Passante, L., Schiffmann, S.N., et al. (2008). An intrinsic mechanism of corticogenesis from embryonic stem cells. Nature *455*, 351–357.

Gato, A., Moro, J.A., Alonso, M.I., Bueno, D., De La Mano, A., and Martin, C. (2005). Embryonic cerebrospinal fluid regulates neuroepithelial survival, proliferation, and neurogenesis in chick embryos. Anat. Rec. *284*, 475–484.

Gortz, P., Siebler, M., Ihl, R., Henning, U., Luckhaus, C., Supprian, T., and Lange-Asschenfeldt, C. (2013). Multielectrode array analysis of cerebrospinal fluid in Alzheimer's disease versus mild cognitive impairment: a potential diagnostic and treatment biomarker. Biochem. Biophys. Res. Commun. *434*, 293–297.

Halassa, M.M., Fellin, T., and Haydon, P.G. (2007). The tripartite synapse: roles for gliotransmission in health and disease. Trends Mol. Med. *13*, 54–63.

Hayashi, A., Le Gal, K., Sodersten, K., Vizlin-Hodzic, D., Agren, H., and Funa, K. (2015). Calcium-dependent intracellular signal pathways in primary cultured adipocytes and ANK3 gene variation in patients with bipolar disorder and healthy controls. Mol. Psychiatry *20*, 931–940.

Hedrich, U.B., Liautard, C., Kirschenbaum, D., Pofahl, M., Lavigne, J., Liu, Y., Theiss, S., Slotta, J., Escayg, A., Dihne, M., et al. (2014). Impaired action potential initiation in GABAergic interneurons causes hyperexcitable networks in an epileptic mouse model carrying a human Na(V)1.1 mutation. J. Neurosci. *34*, 14874–14889.

Illes, S., Theiss, S., Hartung, H.P., Siebler, M., and Dihne, M. (2009). Niche-dependent development of functional neuronal networks from embryonic stem cell-derived neural populations. BMC Neurosci. *10*, 93.

Izsak, J., Seth, H., Andersson, M., Vizlin-Hodzic, D., Theiss, S., Hanse, E., Agren, H., Funa, K., and Illes, S. (2019). Robust generation of person-specific, synchronously active neuronal networks using purely isogenic human iPSC-3D neural aggregate cultures. Front. Neurosci. *13*, 351.

Jantzen, S.U., Ferrea, S., Wach, C., Quasthoff, K., Illes, S., Scherfeld, D., Hartung, H.P., Seitz, R.J., and Dihne, M. (2013). In vitro neuronal network activity in NMDA receptor encephalitis. BMC Neurosci. *14*, 17.

Johnson, M.A., Weick, J.P., Pearce, R.A., and Zhang, S.C. (2007). Functional neural development from human embryonic stem

cells: accelerated synaptic activity via astrocyte coculture. J. Neurosci. *27*, 3069–3077.

Kalamakis, G., Brune, D., Ravichandran, S., Bolz, J., Fan, W., Ziebell, F., Stiehl, T., Catala-Martinez, F., Kupke, J., Zhao, S., et al. (2019). Quiescence modulates stem cell maintenance and regenerative capacity in the aging brain. Cell *176*, 1407–1419.e14.

Kemp, P.J., Rushton, D.J., Yarova, P.L., Schnell, C., Geater, C., Hancock, J.M., Wieland, A., Hughes, A., Badder, L., Cope, E., et al. (2016). Improving and accelerating the differentiation and functional maturation of human stem cell-derived neurons: role of extracellular calcium and GABA. J. Physiol. *594*, 6583–6594.

Kirkeby, A., Grealish, S., Wolf, D.A., Nelander, J., Wood, J., Lundblad, M., Lindvall, O., and Parmar, M. (2012). Generation of regionally specified neural progenitors and functional neurons from human embryonic stem cells under defined conditions. Cell Rep. *1*, 703–714.

Kirwan, P., Turner-Bridger, B., Peter, M., Momoh, A., Arambepola, D., Robinson, H.P., and Livesey, F.J. (2015). Development and function of human cerebral cortex neural networks from pluripotent stem cells in vitro. Development *142*, 3178–3187.

Koch, H., Niturad, C.E., Theiss, S., Bien, C.G., Elger, C., Wandinger, K.P., Vincent, A., Malter, M., Kortvelyessy, P., Lerche, H., et al. (2019). In vitro neuronal network activity as a new functional diagnostic system to detect effects of cerebrospinal fluid from autoimmune encephalitis patients. Sci. Rep. *9*, 5591.

Lancaster, M.A., Renner, M., Martin, C.A., Wenzel, D., Bicknell, L.S., Hurles, M.E., Homfray, T., Penninger, J.M., Jackson, A.P., and Knoblich, J.A. (2013). Cerebral organoids model human brain development and microcephaly. Nature *501*, 373–379.

Livesey, F.J. (2015). Reconstructing the neuronal milieu interieur. Proc. Natl. Acad. Sci. U S A *112*, 6250–6251.

Lugert, S., Basak, O., Knuckles, P., Haussler, U., Fabel, K., Gotz, M., Haas, C.A., Kempermann, G., Taylor, V., and Giachino, C. (2010). Quiescent and active hippocampal neural stem cells with distinct morphologies respond selectively to physiological and pathological stimuli and aging. Cell Stem Cell *6*, 445–456.

Mongiat, L.A., Esposito, M.S., Lombardi, G., and Schinder, A.F. (2009). Reliable activation of immature neurons in the adult hippocampus. PLoS One *4*, e5320.

Obernier, K., and Alvarez-Buylla, A. (2019). Neural stem cells: origin, heterogeneity and regulation in the adult mammalian brain. Development *146*, dev156059.

Otto, F., Illes, S., Opatz, J., Laryea, M., Theiss, S., Hartung, H.P., Schnitzler, A., Siebler, M., and Dihne, M. (2009). Cerebrospinal fluid of brain trauma patients inhibits in vitro neuronal network function via NMDA receptors. Ann. Neurol. *66*, 546–555.

Perez-Alcazar, M., Culley, G., Lyckenvik, T., Mobarrez, K., Bjorefeldt, A., Wasling, P., Seth, H., Asztely, F., Harrer, A., Iglseder, B., et al. (2016). Human cerebrospinal fluid promotes neuronal viability and activity of hippocampal neuronal circuits in vitro. Front. Cell. Neurosci. *10*, 54.

Qian, X., Song, H., and Ming, G.L. (2019). Brain organoids: advances, applications and challenges. Development *146*, dev166074.

Real, M.A., Davila, J.C., and Guirado, S. (2006). Immunohistochemical localization of the vesicular glutamate transporter VGLUT2 in the developing and adult mouse claustrum. J. Chem. Neuroanat. *31*, 169–177.

Rushton, D.J., Mattis, V.B., Svendsen, C.N., Allen, N.D., and Kemp, P.J. (2013). Stimulation of GABA-induced Ca^{2+} influx enhances maturation of human induced pluripotent stem cell-derived neurons. PLoS One *8*, e81031.

Schwarz, N., Hedrich, U.B.S., Schwarz, H., P A, H., Dammeier, N., Auffenberg, E., Bedogni, F., Honegger, J.B., Lerche, H., Wuttke, T.V., et al. (2017). Human cerebrospinal fluid promotes long-term neuronal viability and network function in human neocortical organotypic brain slice cultures. Sci. Rep. *7*, 12249.

Surzenko, N., Crowl, T., Bachleda, A., Langer, L., and Pevny, L. (2013). SOX2 maintains the quiescent progenitor cell state of postnatal retinal Muller glia. Development *140*, 1445–1456.

Tadros, M.A., Lim, R., Hughes, D.I., Brichta, A.M., and Callister, R.J. (2015). Electrical maturation of spinal neurons in the human fetus: comparison of ventral and dorsal horn. J. Neurophysiol. *114*, 2661–2671.

Takazawa, T., Croft, G.F., Amoroso, M.W., Studer, L., Wichterle, H., and Macdermott, A.B. (2012). Maturation of spinal motor neurons derived from human embryonic stem cells. PLoS One *7*, e40154.

Tang, X., Zhou, L., Wagner, A.M., Marchetto, M.C., Muotri, A.R., Gage, F.H., and Chen, G. (2013). Astroglial cells regulate the developmental timeline of human neurons differentiated from induced pluripotent stem cells. Stem Cell Res. *11*, 743–757.

Tchieu, J., Calder, E.L., Guttikonda, S.R., Gutzwiller, E.M., Aromolaran, K.A., Steinbeck, J.A., Goldstein, P.A., and Studer, L. (2019). NFIA is a gliogenic switch enabling rapid derivation of functional human astrocytes from pluripotent stem cells. Nat. Biotechnol. *37*, 267–275.

Tcw, J., Wang, M., Pimenova, A.A., Bowles, K.R., Hartley, B.J., Lacin, E., Machlovi, S.I., Abdelaal, R., Karch, C.M., Phatnani, H., et al. (2017). An efficient platform for astrocyte differentiation from human induced pluripotent stem cells. Stem Cell Reports *9*, 600–614.

Teunissen, C.E., Tumani, H., Bennett, J.L., Berven, F.S., Brundin, L., Comabella, M., Franciotta, D., Federiksen, J.L., Fleming, J.O., Furlan, R., et al. (2009). Consensus guidelines for CSF and blood biobanking for CNS biomarker studies. Mult. Scler. Int. *2011*, 246412.

Vizlin-Hodzic, D., Zhai, Q., Illes, S., Sodersten, K., Truve, K., Parris, T.Z., Sobhan, P.K., Salmela, S., Kosalai, S.T., Kanduri, C., et al. (2017). Early onset of inflammation during ontogeny of bipolar disorder: the NLRP2 inflammasome gene distinctly differentiates between patients and healthy controls in the transition between iPS cell and neural stem cell stages. Transl. Psychiatry *7*, e1010.

Wickham, J., Corna, A., Schwarz, N., Uysal, B., Layer, N., Wuttke, T.V., Koch, H., and Zeck, G. (2020). Human cerebrospinal fluid induces neuronal excitability changes in resected human neocortical and hippocampal brain slices. Front. Neurosci. *14*, 283.

Zappaterra, M.W., and Lehtinen, M.K. (2012). The cerebrospinal fluid: regulator of neurogenesis, behavior, and beyond. Cell. Mol. Life Sci. *69*, 2863–2878.

Stem Cell Reports, Volume *14*

Supplemental Information

Human Cerebrospinal Fluid Promotes Neuronal Circuit Maturation of Human Induced Pluripotent Stem Cell-Derived 3D Neural Aggregates

Julia Izsak, Henrik Seth, Stephan Theiss, Eric Hanse, and Sebastian Illes

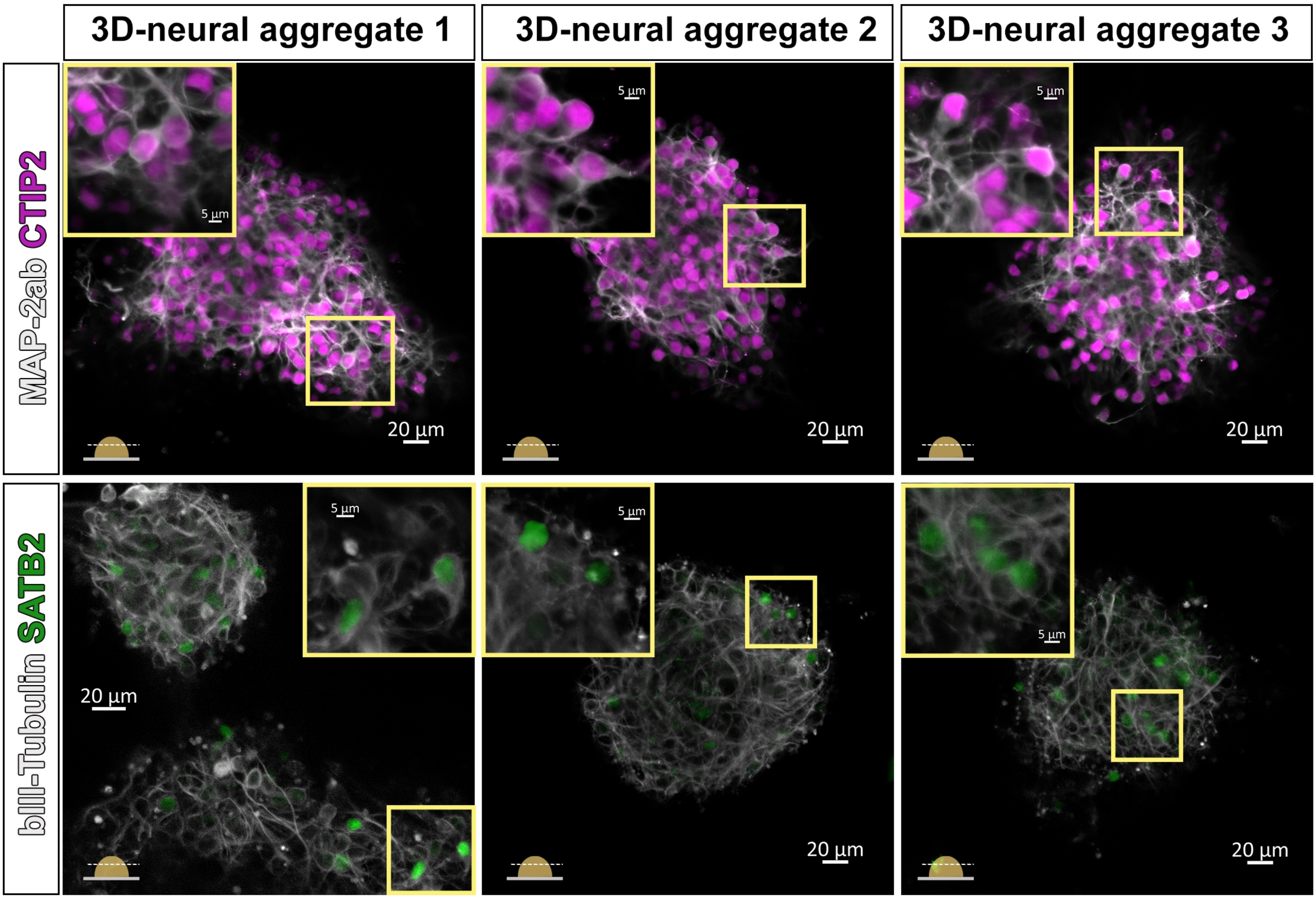
3D-neural aggregate 1
3D-neural aggregate 2
3D-neural aggregate 3
MAP-2ab CTIP2
bIII-Tubulin SATB2
5 µm
20 µm

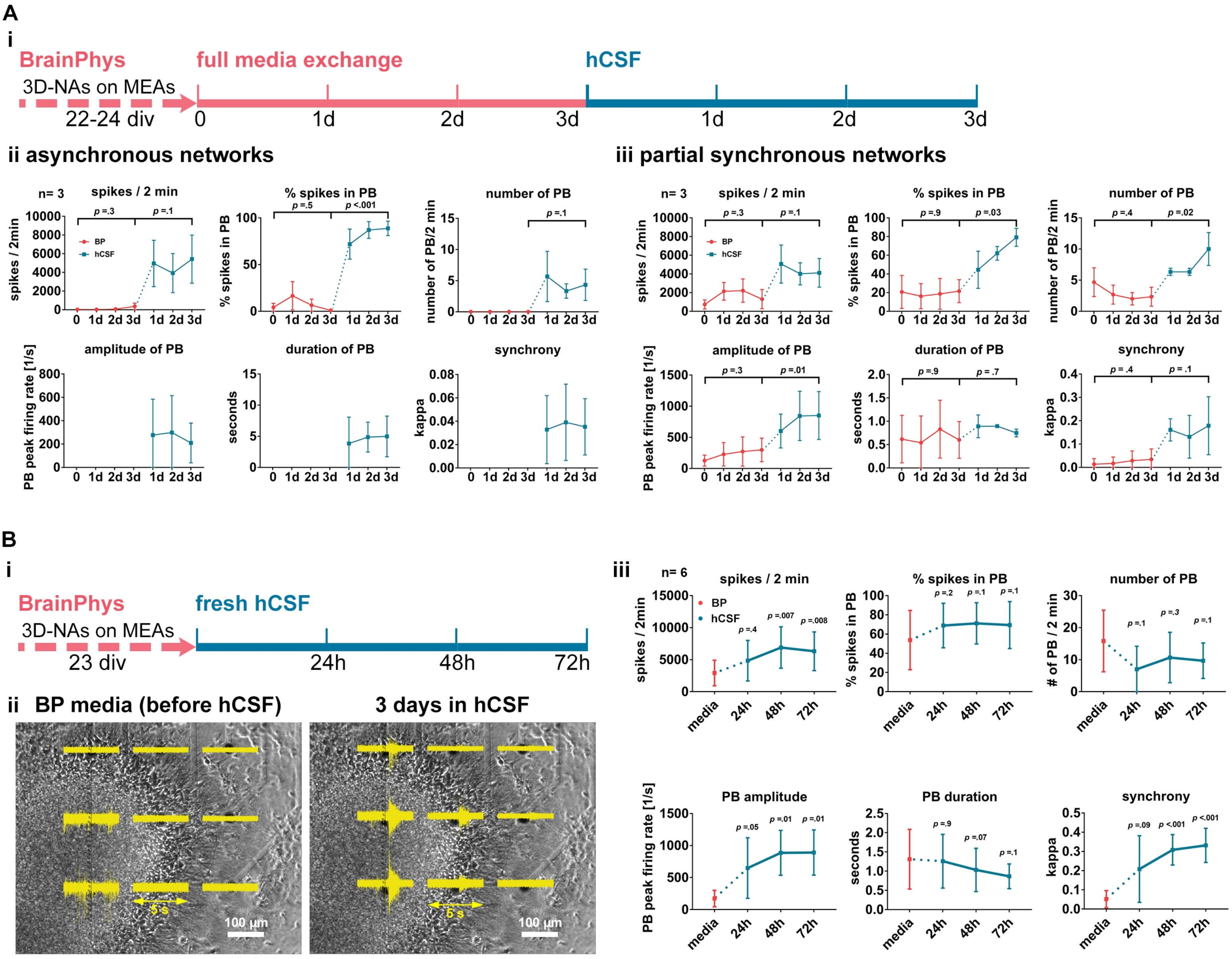
A
i
BrainPhys
full media exchange
hCSF
3D-NAs on MEAs
22-24 div
0
1d
2d
3d
1d
2d
3d
ii asynchronous networks
iii partial synchronous networks
n= 3
spikes / 2 min
% spikes in PB
number of PB
spikes / 2min
% spikes in PB
number of PB/2 min
BP
hCSF
amplitude of PB
duration of PB
synchrony
PB peak firing rate [1/s]
seconds
kappa
B
i
BrainPhys
fresh hCSF
3D-NAs on MEAs
23 div
24h
48h
72h
ii BP media (before hCSF)
3 days in hCSF
5 s
100 µm
iii
n= 6
spikes / 2 min
% spikes in PB
number of PB
# of PB / 2 min
PB amplitude
PB duration
synchrony
media
24h
48h
72h

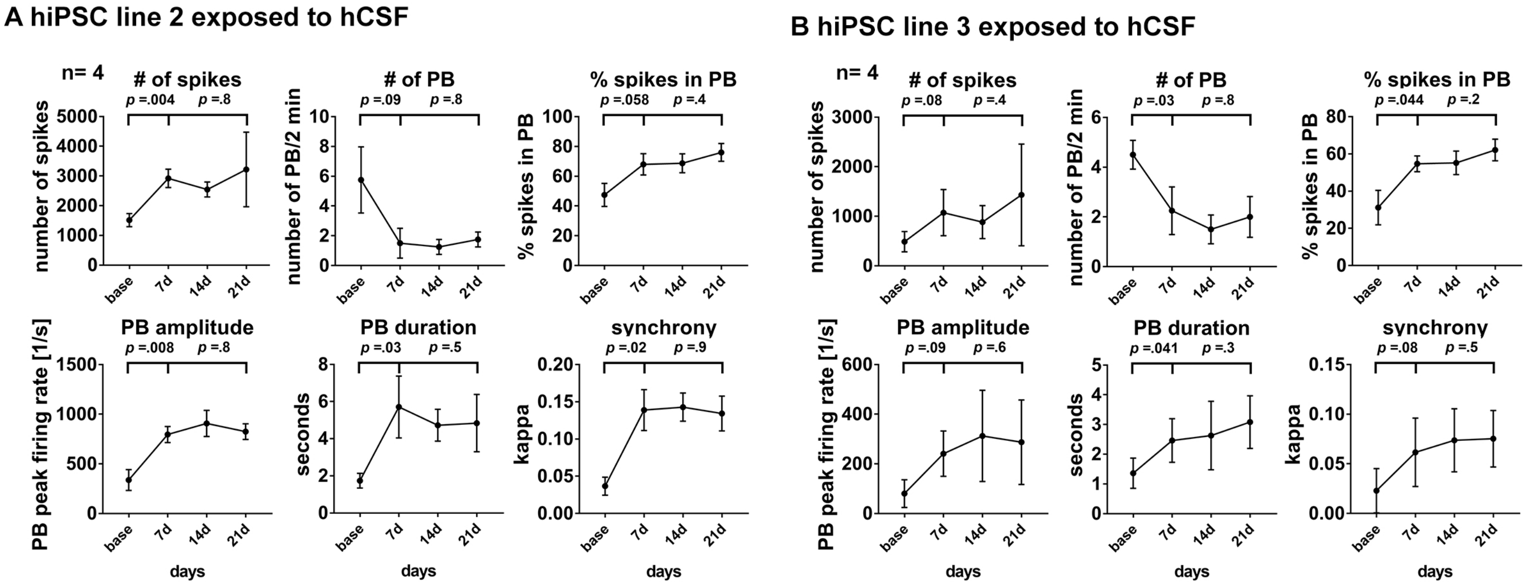
A hiPSC line 2 exposed to hCSF
B hiPSC line 3 exposed to hCSF
n= 4
# of spikes
# of PB
% spikes in PB
PB amplitude
PB duration
synchrony
number of spikes
number of PB/2 min
% spikes in PB
PB peak firing rate [1/s]
seconds
kappa
p =.004
p =.8
p =.09
p =.8
p =.058
p =.4
p =.008
p =.8
p =.03
p =.5
p =.02
p =.9
p =.08
p =.4
p =.03
p =.8
p =.044
p =.2
p =.09
p =.6
p =.041
p =.3
p =.08
p =.5
base
7d
14d
21d
days

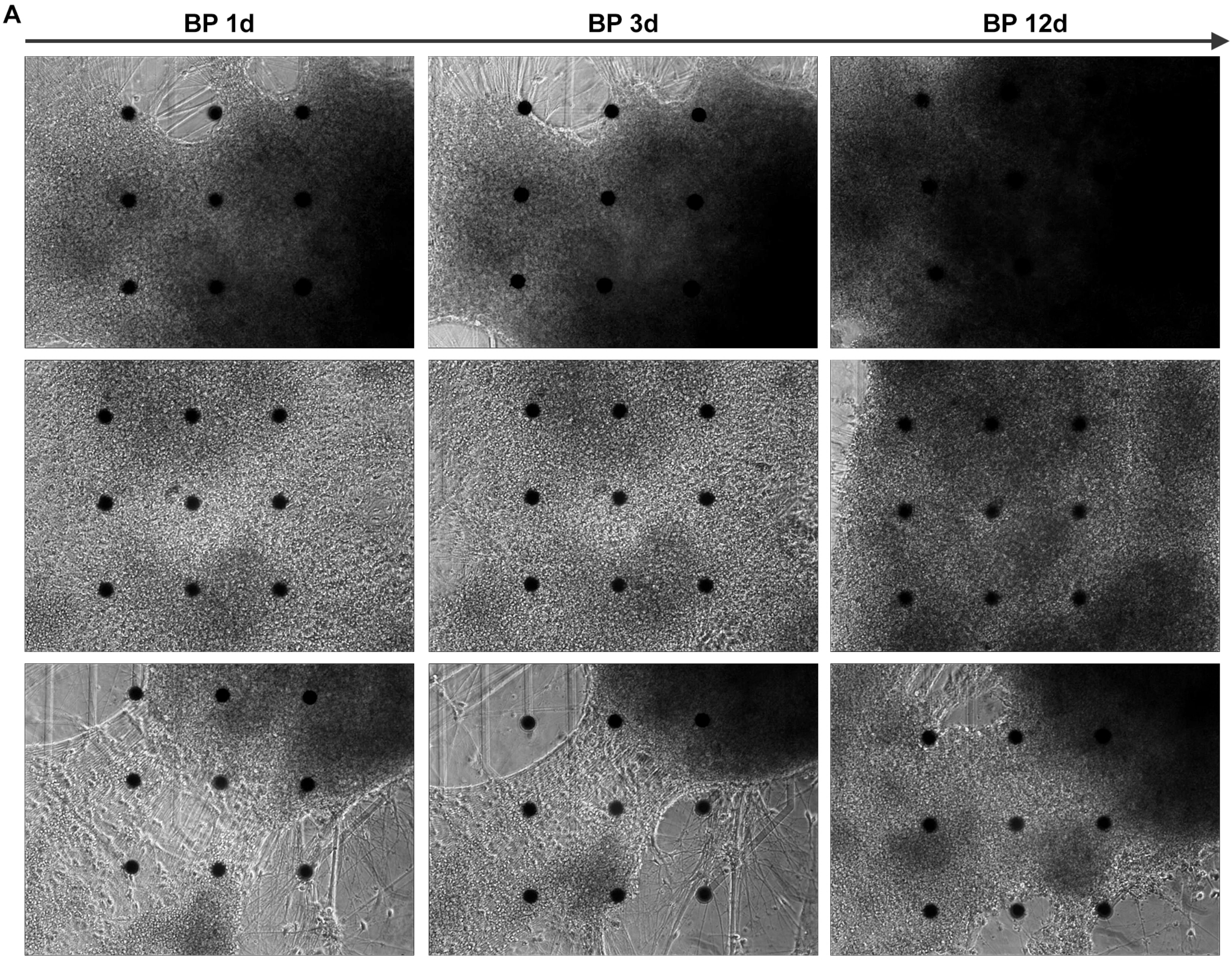
A
BP 1d
BP 3d
BP 12d

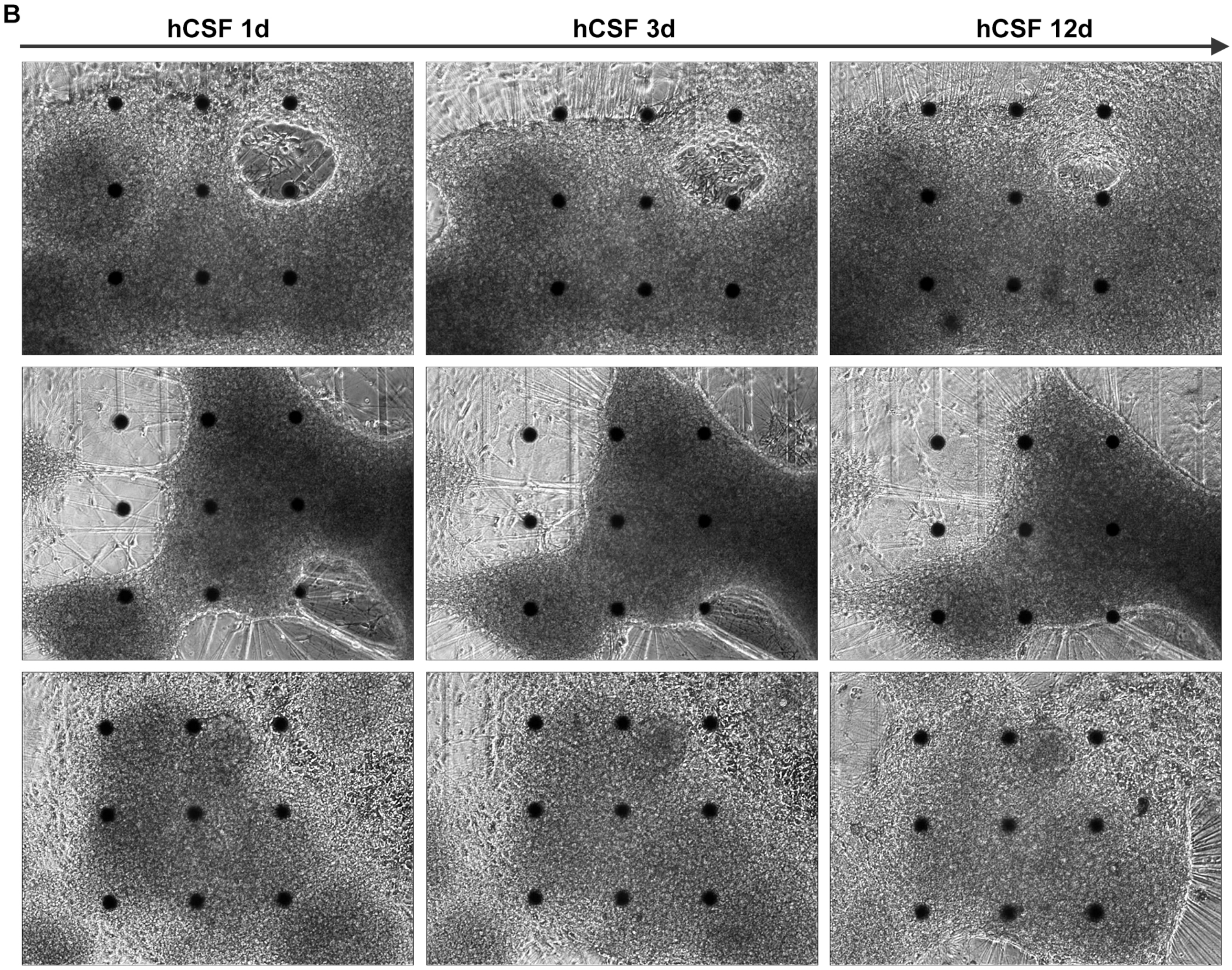
B
hCSF 1d
hCSF 3d
hCSF 12d

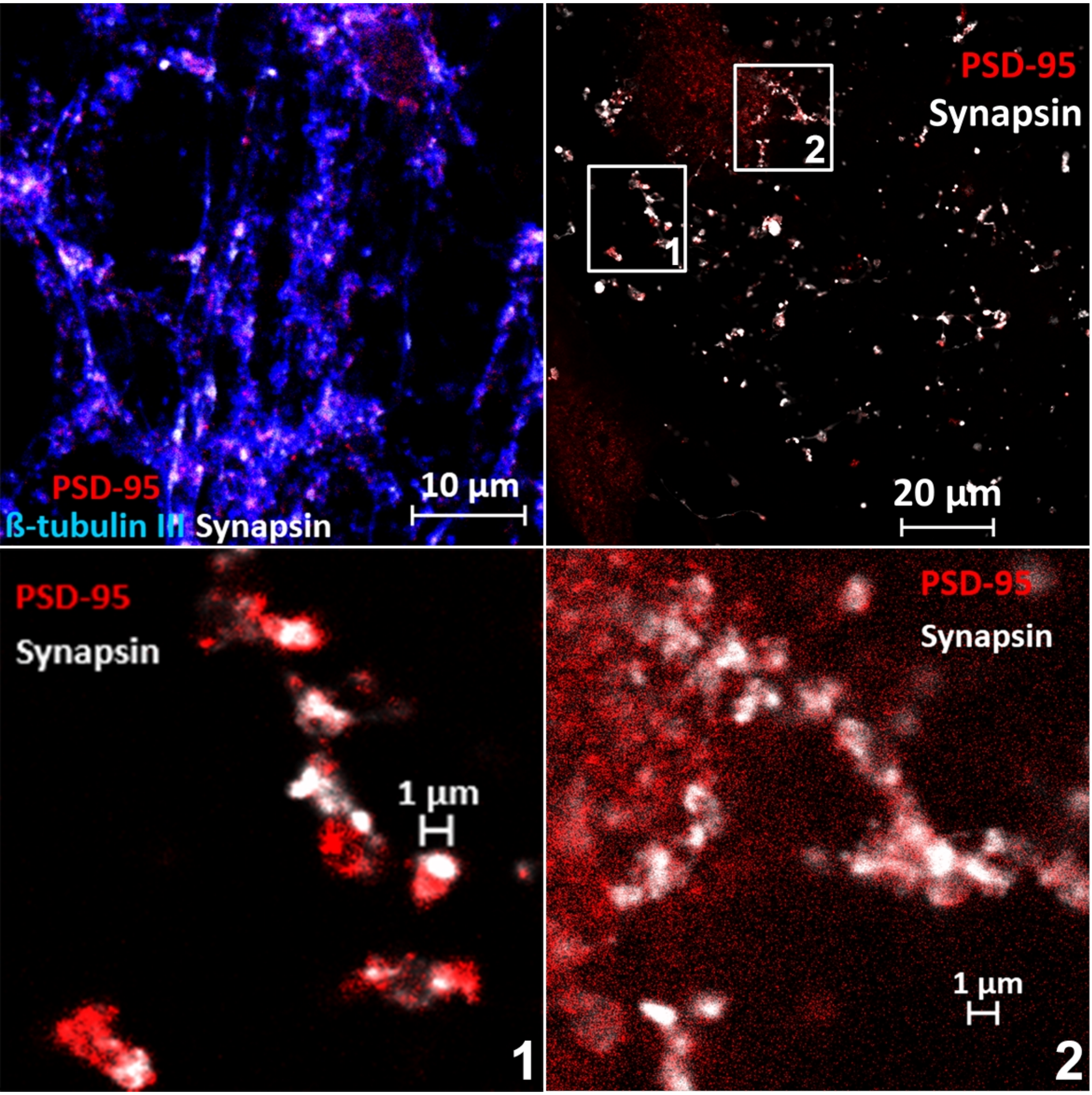
PSD-95
ß-tubulin III Synapsin
10 µm
PSD-95
Synapsin
2
1
20 µm
PSD-95
Synapsin
1 µm
1
PSD-95
Synapsin
1 µm
2

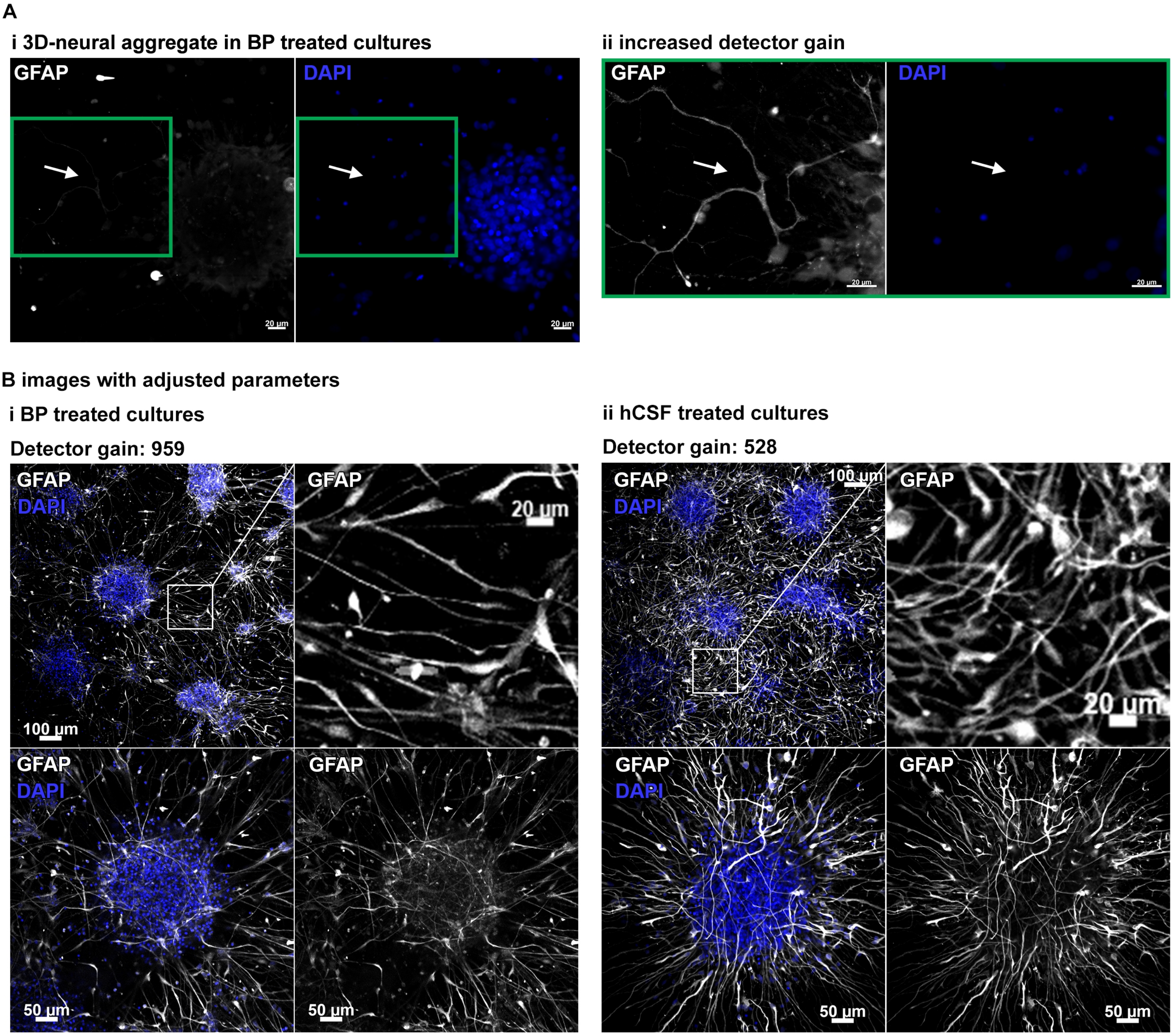
A
i 3D-neural aggregate in BP treated cultures
GFAP
DAPI
20 µm
20 µm
ii increased detector gain
GFAP
DAPI
20 µm
20 µm
B images with adjusted parameters
i BP treated cultures
Detector gain: 959
GFAP
DAPI
100 µm
GFAP
20 µm
GFAP
DAPI
50 µm
GFAP
50 µm
ii hCSF treated cultures
Detector gain: 528
GFAP
DAPI
100 µm
GFAP
20 µm
GFAP
DAPI
50 µm
GFAP
50 µm

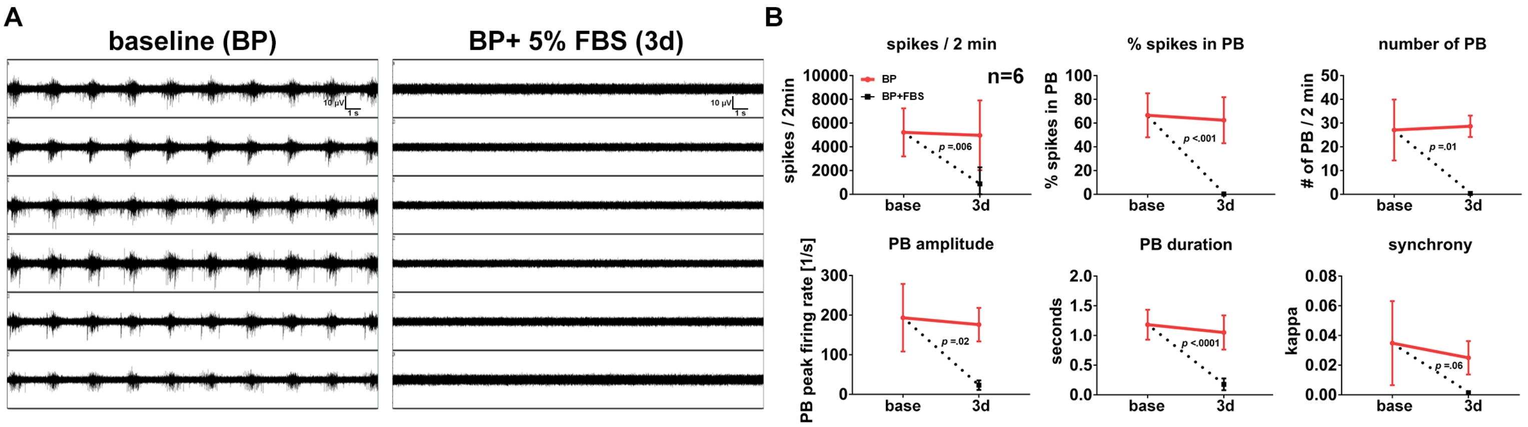
A
baseline (BP)
BP+ 5% FBS (3d)
10 µV
1 s
B
spikes / 2 min
n=6
BP
BP+FBS
spikes / 2min
p =.006
base
3d
% spikes in PB
p <.001
number of PB
# of PB / 2 min
p =.01
PB amplitude
PB peak firing rate [1/s]
p =.02
PB duration
seconds
p <.0001
synchrony
kappa
p =.06

Supplementary material

Title:

Human cerebrospinal fluid promotes neuronal circuit maturation of human induced pluripotent stem cell-derived 3D neural aggregates

Author list:

Julia Izsak[1], Henrik Seth[1], Stephan Theiss[2,3], Eric Hanse[1], Sebastian Illes[1]*

Affiliations:

[1] Institute of Neuroscience and Physiology, Sahlgrenska Academy at University of Gothenburg, Sweden

[2] Institute of Clinical Neuroscience and Medical Psychology, Medical Faculty, Heinrich Heine University, Düsseldorf, Germany

[3] Result Medical GmbH, Düsseldorf, Germany

Contact information:

*Corresponding author: sebastian.illes@neuro.gu.se

Supplemental figure legends

Suppl. Figure 1| Cortical identity of neurons in 3D-neural aggregates

(A) Confocal images show the presence of early-born CTIP2$^+$/MAP2AB$^+$ cortical neurons (up) and late-born SATB2$^+$/bIII-tubulin$^+$-cortical neurons (down) in three different 3D-neural aggregates cultured for 14 days in BrainPhys-based culture media. Boxes mark the ROIs shown in higher magnification. Related to figure 1.

Suppl. figure 2| Control experiments show that full media exchange with BrainPhys-based media does not increase neuronal network activity.

(A) (i) Schematic drawing illustrates the experimental design. Diagrams illustrate the change of neuronal network parameters after full media exchange with BrainPhys-based media and human CSF in (**ii**) asynchronous (n=6) and (**ii**) partially synchronously active neuronal networks (n= 6). Data presented as average values ± standard deviation. **(B) (i)** Schematic drawing shows the experimental design. **(ii)** Phase-contrast images show the morphology of cultures on a nine-electrode array of a six-well MEA, cultured in BrainPhys media (left) and exposed for 72 h to fresh hCSF (right); yellow traces show MEA recordings from the culture. **(iii)** Diagrams illustrate the increase of the network activity after the application of fresh hCSF (n=6). Data presented as average values ± standard deviation. Matched one-way ANOVA with Dunnet correction (baseline compared to indicated group) and Tukey correction (comparison between groups) were applied to calculate indicated *p*-values.Related to figure 2.

Suppl. figure 3| Activity of hiPSC lines 2 and 3 -derived neural networks cultured for 21 days in hCSF.

Diagrams illustrate the increase of network parameters after the application of hCSF and the stable network activity over time in hiPSC line 2 (n=4) **(A)** and hiPSC line 3 (n=4) **(B).** Data presented as average values ± standard deviation. Matched one-way ANOVA with Dunnett correction (baseline compared to indicated group) and Tukey correction (comparison between groups) were applied to calculate indicated p-values. Related to figure 3.

Suppl. figure 4| Morphology of hiPSC 3D neural aggregate cultures exposed to BrainPhys-based media and hCSF.

Phase-contrast images show morphological changes over time of three hiPSC 3D neural aggregate cultures either exposed to **(A)** BrainPhys media or to **(B)** hCSF. Related to figure 3.

Suppl. figure 5| Synapses of hiPSC-derived neurons contain the post-synaptic protein PSD-95 and the pre-synaptic protein Synapsin.

Overview image show the localization of the post-synaptic protein PSD-95 and the pre-synaptic protein Synapsin along ßIII-Tubulin^{+}-neurites and somata from hiPSC-neurons after 21 days cultivation in BrainPhys-based culture media. Detailed confocal imaging demonstrate that PSD-95 and Synapsin puncta appear next to each other. Related to figure 6.

Suppl. figure 6| Visualization of GFAP^{+} astrocytes requires high detector gain in BrainPhys treated cultures

(A) (i) Confocal images shows that the visualization of GFAP^{+}-astroglial cells in 3D-neural aggregate cultures under BrainPhys cannot be achieved by using the same detector gain level as used for hCSF-treated cultures. The inset marks an area which contains a GFAP^{+}-astroglial cell (arrow) that can be visualized by increasing the detector gain (**ii**). **(B)** Confocal images visualize the GFAP^{+}-astroglial cell in 3D-neural aggregate cultures under **(i)** BrainPhys and **(ii)** hCSF, with detailed images showing the outside and inside regions. Note the different applied detector gains to visualize GFAP^{+}-astroglial cell under BrainPhys and hCSF. Related to figure 7.

Suppl. figure 7| Fetal bovine serum suppresses neuronal network activity in hiPSC-derived neural networks

(A) Representative examples of MEA recordings in BrainPhys media left) and after three days exposure to 5% fetal bovine serum (right) **(B)** diagrams illustrate the decrease of network activity after the application of fetal bovine serum. Synchronous networks turn into nearly inactive cultures when exposed to fetal bovine serum. (control: n=6, fetal bovine serum: n=6). Data presented as average values ± standard deviation. *p*-values were calculated by paired student's *t*-test. Related to Figure 2.

Supplementary videos

Note, all suppl. videos contain description about video content.

Suppl. video 1| Z-stack series and 3D projection of confocal images shows bIII-tubulin^{+}-neurons and DAPI-nuclei in 3D-neural aggregates cultured in BrainPhys-based media or hCSF.

Related to figure 6.

Suppl. video 2| Z-stack series and 3D projection of confocal images shows MAP2AB^{+}-neurons and DAPI-nuclei in 3D-neural aggregates cultured in BrainPhys-based media or hCSF.

Related to figure 6.

Suppl. video 3| 3D projection of confocal images shows S100beta^{+}-astrocytes and DAPI-nuclei in 3D-neural aggregates cultured in BrainPhys-based media or hCSF.

Related to figure 7.

Suppl. video 4| Z-stack series of confocal images shows S100beta^{+}-astrocytes and DAPI-nuclei in 3D-neural aggregates cultured in BrainPhys-based media or hCSF.

Related to figure 7.

Suppl. video 5| Color-coded depth visualisation shows the different height of 3D-neural aggregates cultured in BrainPhys-based media or hCSF.

Related to figure 7.

Supplementary table

Suppl. table 1: List of primary and secondary antibodies used for the study

Primary antibody	Supplier (cat. No)	Host	Dilution
GFAP	Pharmingen (556327)	mouse	1:500
S1oo beta	Dako Agilent (Z0311)	rabbit	1:500
Aquaporin 4	Atlas antibodies (HPA014784)	rabbit	1:500
Glutamine synthetase	Chemicon (MAB302)	mouse	1:500
Ki-67	Santa Cruz (sc-15402)	rabbit	1:500
Sox2	R&D (AF2018)	goat	1:500
Map2ab	Abcam (ab11267)	mouse	1:1000
bIII-tubulin	R&D (MAB1195)	mouse	1:2000
PSD-95	Abcam (ab18258)	rabbit	1:1000
Synapsin	Santa Cruz (sc-8295)	goat	1:1000
vGLUT1	Synaptic systems (135303)	rabbit	1:1000
CTIP2	Abcam (ab18465)	rat	1:500
SATB2	Abcam (ab51502)	mouse	1:500
caspase-3	Abcam (ab13847)	rabbit	1:500
Nestin	Merck (MAB353)	mouse	1:500

Secondary antibody	Supplier (cat. No)	Host	Anti-	Dilution
Alexa Fluor 488	Thermo Fisher (A11029)	goat	mouse	1:500
Alexa Fluor 488	Thermo Fisher (A21208)	donkey	rat	1:500
Alexa Fluor 555	Thermo Fisher (A31572)	donkey	rabbit	1:500
Alexa Fluor 488	Thermo Fisher (A11055)	donkey	goat	1:500
Alexa Fluor 405	Thermo Fisher (A31553)	goat	mouse	1:500
Alexa Fluor 555	Thermo Fisher (A21432)	donkey	goat	1:500

Supplementary experimental procedures

Ethical statement

We confirm that all methods were carried out in accordance with relevant guidelines and regulations. We confirm that all experimental protocols were approved by the named institutions. Informed consent was obtained from all subjects. Work with human iPSC lines were approved by a local ethics committee (Regionala etikprövningsnämnden i Göteborg, with the ethical approval number: DNR 172-08. The procedure of CSF sampling and CSF samples application for research purposes was approved by a local ethics committee (Regionala etikprövningsnämnden i Göteborg, with the ethical approval number: DNR 942-12).

Generation of human iPSC-3D neural aggregates

Human iPSC cell lines (C1, C2, C3) were cultured and differentiated into cortical neural stem cells as described elsewhere (Hayashi et al., 2015; Vizlin-Hodzic et al., 2017). Shortly, hiPSCs were cultured under feeder-free conditions in Cellartis DEF-CS™ (Takara Bio Europe AB) or mTesR at 37°C in a humidified atmosphere of 5% CO_2. For neural induction of hiPSCs, the DUAL-SMAD inhibition protocol was applied, as previously established in (Shi et al., 2012). 20-30 days post neural induction, human iPSC-NSC cultures were passaged with Accutase and were frozen in 10%-DMSO solution (solved in DMEM/F12). The cryostocks were preserved at -152°C. For 3D-cortical aggregate formation, frozen cryostocks of hiPSC-NSC were thawed and 1.0×10^6 cells were cultured in neural culture media on laminin [20 µg/ml]-coated 3.5 cm culture plates. Neural culture media consisting of DMEM/F12 GlutaMAX, Neurobasal, 1x N2 supplement, 1x B27 supplement, 5µg ml-1 insulin, 1 mM Ultra glutamine, 100 µM non-essential amino acids, 100 µM 2-mercaptoethanol, 50 U ml-1 penicillin and streptomycin/gentamicin. Within 10-14 days, hiPSC-NSC formed 3D-neural aggregates (Edri et al., 2015; Izsak et al., 2019) and 3D-neural aggregates with diameters ≤150 µM were manually transferred on PDL/laminin-coated coverslips or MEAs. For neuronal differentiation, BrainPhys-media supplemented with N2 supplement, B27 with vitamin A, 2 mM Ultra glutamine, 50 U ml^{-1} Pen/Strep, and 200 µM ascorbic acid were used. Half media exchanges were performed twice a week. To promote neuronal differentiation, DAPT [10µM] and human BNDF, GDNF, TGF-β, were added [20 ng/ml] to the cultivation media.

Multi-electrode array recordings

Two to five hiPSC 3D-neural aggregates were seeded as a 5 µl drop directly on PDL/laminin coated electrode arrays of 6-well PEDOT-CNT-MEAs. After 1 h, BrainPhys media with supplements (described above) was added. Half media exchanges were performed twice a week.

MEAs had a square grid of 9 planar Ti/TiAu electrodes with PEDOT-CNT (carbon nanotube poly-3,4-ethylene-dioxythiophene) of 30 µm diameter and 200 µm spacing. The 9 planar electrodes can record spontaneous activity of neurons in a 50-100 µm radius. Baseline recordings have been performed in BrainPhys media with supplements prior to the application of either HEPES buffered hCSF or fresh culture media. The signals from the 9 electrodes were simultaneously sampled at 25 kHz, and stored using the MC_Rack software provided by Multi Channel Systems. MEA electrodes had an input impedance of 30–50 kΩ according to the specifications of the manufacturer (Multi Channel Systems). Offline-spike detection was performed by the SPANNER software suite (RESULT Medical; see also (Illes et al., 2014)). Synchronous network activity was analyzed by population burst (PB) detection using custom-built Matlab software (Hedrich et al., 2014; Izsak et al., 2019). As described in Hedrich et al, (2014), for the quantification of firing synchrony across pairs of electrodes, spikes were collected in 10-ms-wide bins and subsequently dichotomized to either zero spikes or at least one spike. Consequently, either both electrodes were active during a time bin (“coincident bin”), only one electrode was active, or both were silent. Cohen's κ statistic then captures the proportion of observed coincident bins exceeding the chance expected proportion of coincidences. Similar to Pearson's correlation coefficient, κ values lie in the range −1 to +1. The average κ value of all electrode pairs with a firing rate of at least 30 spikes/min was calculated as a measure of the overall synchrony of a recording.

Whole-cell patch clamp recordings and data analysis

Five to ten hiPSC 3D-neural aggregates were seeded on PDL/laminin coated coverslips and cultured with BrainPhys media with supplements. Half media exchanges were performed twice a week. 14-20 days after differentiation a small plastic cylinder was placed on top of each position and filled with either BrainPhys or hCSF. Subsequently, each coverslip contained two population of cells, one exposed to BrainPhys and one exposed to hCSF.Three days after treatment, patch-clamp recordings were performend. The coverslips were mounted under a differential interference microscope (Nikon E600FN) together with a CCD camera (Sony XC-73CE) to visually identify the cells and to visualize the recording electrode connected to the neuron via a borosilicate glass micropipette (resistance 3-6 MΩ). Cells were perfused (2-3 ml/min) with artificial CSF (aSCF) containing: 1 mM NaH_2PO_4, 123 mM NaCl, 26 mM $NaHCO_3$, 3 mM KCl, 1 mM $MgCl_2$, 2 mM $CaCl_2$, and 10 mM D-glucose. The micropipette was filled with an intracellular solution containing; 127 mM K-gluconate, 8 mM KCl, 10 mM HEPES, 15 mM phosphocreatine, 4 mM Mg-ATP, 0.3 mM Na-GTP (pH ~7.3 and osmolality

280–300 mOsm). Patch-clamp recordings were performed on cells at the edge of 3D-neural aggregates visually identified using infrared differential interference contrast video microscopy. The data was collected with a sampling frequency of 10 kHz and filtered at 3 kHz by an EPC-9 amplifier (HEKA Elektronik, D-67466 Lambrecht/Pfalz, Germany). After opening, the cell was allowed to rest for 5 minutes before recordings started. Series resistance was monitored using a 20 ms 10 mV hyperpolarizing pulse. The series resistance was not allowed to exceed 20 MΩ in whole-cell recordings, or to change more than 20% during an experiment, otherwise the experiment was discarded. Whole-cell recordings were carried out at 32 °C.

The firing response to step-wise current injections (800 ms) was recorded in whole-cell current-clamp and spontaneous synaptic activity (i.e. EPSCs and IPSCs) was recorded in whole-cell voltage-clamp. For spontaneous synaptic activity cells were clamped at -70 mV for recordings of α-amino-3-hydroxy-5-methyl-4-Isoxazolepropionic acid receptor (AMPAR) mediated excitatory postsynaptic currents (EPSCs) and at 0 mV for recordings of γ-aminobutyric acid receptor (GABAR) mediated inhibitory postsynaptic currents (IPSCs). All recordings were performed between second (14 days) and third week (21 days) in vitro.

Spontaneous synaptic activity, i.e. frequency and amplitude, was analyzed in Minianalysis 6.0.3 (Synaptosoft, Fort Lee, NJ, USA). Input resistance was calculated using Ohm's law (U = R x I) after injecting a 50 pA depolarizing current in current-clamp. To calculate the action potential threshold, we constructed phase-plane plots by plotting the first derivative (i.e. the rate of change) of the membrane potential during the first action potential, at current injection, against the membrane potential (Fig. 5E). These plots visualize some aspects of the action potential very clearly (i.e. threshold de- and repolarization as well as amplitude). Threshold was then estimated at a derivative (dV/dt) of 10 mV/ms. Calculations and data analysis were performed in custom-made IGOR Pro 8 (WaveMetrics, Lake Oswego, OR, USA) software.

Human CSF sample collection

Human CSF was collected by lumbar puncture from healthy volunteers (two women and four men, 20-31 years) (Forsberg et al., 2019). The volunteers were recruited via social media and they provided written informed consent. No anesthesia was used. Samples were immediately centrifuged to separate cells from CSF, and 1.5-2 ml aliquots were frozen to -80°C. For further validation of the presented approach, additional de-identified, left-over control hCSF samples (n= six women and three men, 67-89 years old) provided by Sahlgrenska University Hospital, Sweden, were used. The hCSF sampling procedure and classification as control hCSF samples

is according an international consensus protocol (Teunissen et al., 2009). For sampling of fresh hCSF, 12 ml human CSF were obtained via lumbar puncture from a 38-year old male without any neurological symptoms. Additional blood samples were collected at the same time of hCSF sampling. Since IgG level within the blood sample was in the normal range, we exclude acute infection or any other diseases occurring in this human individual. Immediately after lumbar puncture, CSF samples were centrifuged, 11 ml supernatant was collected and stored on ice for 40 min prior direct application to the experiment.

Immunocytochemistry, image acquisition and analyses

For immunocytochemical investigations, cultured hiPSC-3D neural aggregates (control and hCSF treated) were washed in phosphate-buffered saline (PBS), pH 7.2 and fixed for 20 min in 4% paraformaldehyde at room temperature. After fixation, the cells were incubated with 1% BSA for 30 min. Primary antibodies diluted in blocking solution with 0,025 Triton-X were applied at 4°C overnight. After washing in PBS, appropriate secondary antibodies coupled were applied for 2 h at room temperature. Images were collected with a confocal-laser scanning microscope (LSM 700 META Zeiss). All the images were taken with the same image settings per marker. For the overview images and quantification of GFAP+ area coverage, one image per coverslip was taken with an 10x objective. For the rest of the image quantification, five images with a 2 µm optical slice were randomly taken from each coverslip with an 40x water-objective. The raw images were exported as TIFF files by using Zen Blue software. Mean signal intensity values for the different channels were obtained from image acquisition raw data, and further quantification was performed by using the Fiji-plug-in as part of the ImageJ software (Schindelin et al., 2012). The neuronal processes (bIII-tubulin) and coverage by $GFAP^+$ astrocytes were quantified by measuring the percentage of area covered across the image. The number of $MAP2AB^+$ neurons and $S100ß^+$ astrocytes were manually quantified using Cell Counter in ImageJ and normalized per number of total DAPI nuclei. The $DAPI^+$ cell nuclei, the $SOX\text{-}2^+$ and $Ki\text{-}67^+$ nuclei, were quantified by the Nucleus counter plugin (Schneider et al., 2012). For the quantification of $PSD\text{-}95^+$ synapses, 50 µm x 50 µm square ROIs were selected from each image and exported. The PSD-95 particles in the ROIs were counted using the Particle analysis tool implemented in ImageJ (Schneider et al., 2012). For the $vGLUT1^+$ synapses, in order to remove the cytoplasmatic signal, a reduced detector gain was used at image acquisition to visualize only the intense synaptic puncta. The synaptic $vGLUT1^+$ puncta were quantified by the Particle analysis tool and normalized per number of $MAP2ab^+$ neurons. To achieve a reliable quantitative assessment of the PSD-95 and vGlut1 signals in different sized

3D neural aggregates, first measured the total height of 3D neural aggregates by using the DAPI-visualized nuclei as references. Here, we defined that the top of 3D neural aggregates is 100% and the bottom of 3D neural aggregates is 0%. Then we imaged 2 µm thick optical slices at a z-level position that represented 80% of the total height of each individual 3D neural aggregate. In these optical slices, we compared the percentage of area covered by PSD-95$^+$ post-synapses and vGlut1$^+$ pre-synapses as well as the number of PSD-95$^+$ post-synaptic and vGlut1$^+$ pre-synaptic dots in BP-based media and three days hCSF treated 3D neural aggregates. For vGlut1, we normalized these values to the number of MAP2AB$^+$ neurons counted in the same image field.

All the image quantification was manually revised to exclude false signal detection. The measurement of 3D-NA cluster height was done live by using the z-stack method in the Zen Black image acquisition software. For the z-stack videos of the 3D-neural aggregates 2,5 µm optical slices were taken with an interval of 2,2 µm (15-30 slices per aggregate). The raw and depth-coded z-stack projections were exported with the use of Zen Blue software. The used primary and secondary antibodies are summarized in suppl. table 1.

Statistical analysis

For statistical analysis either matched one-way ANOVA with Dunnet correction (baseline compared to indicated group) and Tukey correction (comparison between groups) were applied or two-way, unpaired *t*-test were applied to calculate indicated *p*-values. All presented data show mean value ± standard deviation (SD), *n* refers to the number of individual cultures treated with hCSF or BrainPhys media and N refers to the number of individual experiments. For statistical analysis, GraphPad Prism 8.0 software was used.
